# Supplementary material for: Systematic review and meta-analysis of the prevalence of common respiratory viruses in children < 2 years with bronchiolitis in the pre-COVID-19 pandemic era
Source: PLoS One. 2020 Nov 12;15(11):e0242302. doi: 10.1371/journal.pone.0242302 (PMC7660462; doi:10.1371/journal.pone.0242302)

S1 Table. Preferred reporting items for systematic reviews and meta-analyses checklist

Section/topic	#	Checklist item	Reported on page #
**TITLE**			
Title	1	Identify the report as a systematic review, meta-analysis, or both.	1
**ABSTRACT**			
Structured summary	2	Provide a structured summary including, as applicable: background; objectives; data sources; study eligibility criteria, participants, and interventions; study appraisal and synthesis methods; results; limitations; conclusions and implications of key findings; systematic review registration number.	2-3
**INTRODUCTION**			
Rationale	3	Describe the rationale for the review in the context of what is already known.	4-5
Objectives	4	Provide an explicit statement of questions being addressed with reference to participants, interventions, comparisons, outcomes, and study design (PICOS).	5
**METHODS**			
Protocol and registration	5	Indicate if a review protocol exists, if and where it can be accessed (e.g., Web address), and, if available, provide registration information including registration number.	6
Eligibility criteria	6	Specify study characteristics (e.g., PICOS, length of follow-up) and report characteristics (e.g., years considered, language, publication status) used as criteria for eligibility, giving rationale.	7
Information sources	7	Describe all information sources (e.g., databases with dates of coverage, contact with study authors to identify additional studies) in the search and date last searched.	6
Search	8	Present full electronic search strategy for at least one database, including any limits used, such that it could be repeated.	6
Study selection	9	State the process for selecting studies (i.e., screening, eligibility, included in systematic review, and, if applicable, included in the meta-analysis).	7
Data collection process	10	Describe method of data extraction from reports (e.g., piloted forms, independently, in duplicate) and any processes for obtaining and confirming data from investigators.	7
Data items	11	List and define all variables for which data were sought (e.g., PICOS, funding sources) and any assumptions and simplifications made.	7

Risk of bias in individual studies	12	Describe methods used for assessing risk of bias of individual studies (including specification of whether this was done at the study or outcome level), and how this information is to be used in any data synthesis.	8
Summary measures	13	State the principal summary measures (e.g., risk ratio, difference in means).	8
Synthesis of results	14	Describe the methods of handling data and combining results of studies, if done, including measures of consistency (e.g., I^2) for each meta-analysis.	8

**Section/topic**	**#**	**Checklist item**	**Reported on page #**
Risk of bias across studies	15	Specify any assessment of risk of bias that may affect the cumulative evidence (e.g., publication bias, selective reporting within studies).	8
Additional analyses	16	Describe methods of additional analyses (e.g., sensitivity or subgroup analyses, meta-regression), if done, indicating which were pre-specified.	8
**RESULTS**			
Study selection	17	Give numbers of studies screened, assessed for eligibility, and included in the review, with reasons for exclusions at each stage, ideally with a flow diagram.	9 and Fig. 1
Study characteristics	18	For each study, present characteristics for which data were extracted (e.g., study size, PICOS, follow-up period) and provide the citations.	9
Risk of bias within studies	19	Present data on risk of bias of each study and, if available, any outcome level assessment (see item 12).	9
Results of individual studies	20	For all outcomes considered (benefits or harms), present, for each study: (a) simple summary data for each intervention group (b) effect estimates and confidence intervals, ideally with a forest plot.	9
Synthesis of results	21	Present results of each meta-analysis done, including confidence intervals and measures of consistency.	12
Risk of bias across studies	22	Present results of any assessment of risk of bias across studies (see Item 15).	12
Additional analysis	23	Give results of additional analyses, if done (e.g., sensitivity or subgroup analyses, meta-regression [see Item 16]).	13
**DISCUSSION**			
Summary of evidence	24	Summarize the main findings including the strength of evidence for each main outcome; consider their relevance to key groups (e.g., healthcare providers, users, and policy makers).	13
Limitations	25	Discuss limitations at study and outcome level (e.g., risk of bias), and at review-level (e.g., incomplete retrieval of identified research, reporting bias).	16

Conclusions	26	Provide a general interpretation of the results in the context of other evidence, and implications for future research.	17
**FUNDING**			
Funding	27	Describe sources of funding for the systematic review and other support (e.g., supply of data); role of funders for the systematic review.	17

From: Moher D, Liberati A, Tetzlaff J, Altman DG, The PRISMA Group (2009). Preferred Reporting Items for Systematic Reviews and Meta-Analyses: The PRISMA Statement. PLoS Med 6(7): e1000097. doi:10.1371/journal.pmed1000097

S2 Table. Search strategy in Medline (Pubmed)

**Search**	**Virus**
#1	“HRSV” OR “RSV” OR “human respiratory syncytial virus”OR “respiratory syncytial virus” OR “HRSV-A” OR “HRSV-B” OR “HMPV” OR “MPV” OR “human metapneumovirus”OR “metapneumovirus” OR “HMPV-A” OR “HMPV-B” OR “HAdV” OR “AdV” OR “Adenovirus” OR “Adenovirus Infections, Human” OR “Human adenovirus” OR “HADV-A” OR “HADV-B” OR “HADV-C” OR “HADV-D” OR “HADV-E” OR “HADV-F” OR “HADV-G” OR “HBoV” OR “BoV” OR “Bocavirus” OR “Bocavirus Infections, Human” OR “Human Bocavirus” OR “HCoV” OR “CoV” OR “Coronavirus” OR “Coronavirus Infections, Human” OR “Human Coronavirus” OR “229E” OR “OC43” OR “NL63” OR “HKU1” OR “HCoV-229E” OR “HCoV-OC43” OR “HCoV-NL63” OR “HCoV-HKU1” OR “HPIV” OR “PIV” OR “Parainfluenzavirus” OR “Parainfluenzavirus Infections, Human” OR “Human Parainfluenzavirus” OR “PIV-1” OR “PIV-2” OR “PIV-3” OR “PIV-4” OR “HPIV-1” OR “HPIV-2” OR “HPIV-3” OR “HPIV-4” OR “HEV” OR “EV” OR “Enterovirus” OR “Enterovirus Infections, Human” OR “Human Enterovirus” OR “HRV” OR “RV” OR “Rhinovirus” OR “Rhinoviruses” OR “Rhinovirus Infections, Human” OR “Human Rhinovirus” OR “RV-A” OR “RV-B” OR “RV-C” OR “Influenza” OR “Inf” OR “Influenza virus” OR “Influenza, Human” OR “Influenza-A virus” OR “Influenza-B virus” OR “Influenza-C virus”
#2	bronchiolitis
#3	#1 AND #2

S3 Table. Items for risk of bias assessment

**Hoy et al. tool for cross sectional studies**	**Yes (1)/No (0)**
**External validity**	
1. Was the study's target population a close representation of the national population in relation to relevant variables?	**1**
2. Was the sampling frame a true or close representation of the bronchiolitis population?	**1**
3. Was some form of random selection used to select the sample, OR was a census undertaken?	**1**
4. Was the likelihood of non response bias minimal?	**1**
**Internal validity**	
5. Were data collected directly from the subjects (as opposed to a proxy)?	**1**
6. Was an acceptable bronchiolitis case definition used in the study?	**1**
7. Was the study viral detection assay shown to have validity and reliability?	**1**
8. Was the same mode type of sample collected for all subjects?	**1**
9. Was the length of the shortest prevalence period for the parameter of interest appropriate?	**1**
10. Were the numerator(s) and denominator(s) for the parameter of interest appropriate?	**1**
Total score	**10**
**Interpretation of the risk of bias tool**<br><br>• 7-10: Low risk of bias<br>• 4-6: Moderate risk of bias<br>• 0-3: High risk of bias	

S4 Table. Main reasons of exclusion of eligible studies

N°	Name, Year	Title	Reason of exclusion
1	Akhras, 2010	Human metapneumovirus and respiratory syncytial virus: subtle differences but comparable severity	No molecular assays used
2	Alonso, 2007	Bronchiolitis due to respiratory syncytial virus in hospitalized children: a study of seasonal rhythm.	Only positive samples included
3	Al-Shawwa, 2007	Clinical and therapeutic variables influencing hospitalisation for bronchiolitis in a community-based paediatric group practice	No molecular assays used
4	Al-Shehri, 2005	Bronchiolitis in Abha, Southwest Saudi Arabia: viral etiology and predictors for hospital admission.	No molecular assays used
5	Alvarez, 2013	Epidemiological and genetic characteristics associated with the severity of acute viral bronchiolitis by respiratory syncytial virus.	Systematic review
6	Amér, 2009	[Not only RSV can cause bronchiolitis in small children	Full text not found
7	Bakalovic, 2015	Epidemiological Features of Bronchiolitis in the Pediatric Clinic of Clinical center of Sarajevo University.	Data on detection assay not reported
8	Barr, 2018	Change in viral bronchiolitis management in hospitals in the UK after the publication of NICE guideline.	No data on viral etiology searched
9	Bashir, 2017	Respiratory syncytial virus and influenza are the key viral pathogens in children <2 years hospitalized with bronchiolitis and pneumonia in Islamabad Pakistan.	Not possible to extract data on viral etiology searched
10	Bauert, 2019	Rhinovirus Species in Children With Severe Bronchiolitis: Multicenter Cohort Studies in the United States and Finland	Duplicates
11	Bennett, 2018	Population-based trends and underlying risk factors for infant respiratory syncytial virus and bronchiolitis hospitalizations.	Inappropriate study population
12	Berezin, 2006	Rhinovirus and bronchiolitis.	Comments
13	Bilavsky, 2010	Respiratory syncytial virus-positive bronchiolitis in hospitalized infants is associated with thrombocytosis.	No molecular assays used
14	Bochkov, 2020	A 14-year Prospective Study of Human Coronavirus Infections in Hospitalized Children: Comparison With Other Respiratory Viruses.	Inappropriate study population
15	Bouscambert-Duchamp, 2005	Detection of human metapneumovirus RNA sequences in nasopharyngeal aspirates of young French children with acute bronchiolitis by real-time reverse transcriptase PCR and phylogenetic analysis.	Duplicate of Janahi, 2017
16	Boyce, 2004	Incidence of bronchiolitis-associated hospitalization among children in Olmsted County, Minnesota.	Comments
17	Boyce, 2016	More on Viral Bronchiolitis in Children.	Comments

18	Bradley, 2005	Severity of respiratory syncytial virus bronchiolitis is affected by cigarette smoke exposure and atopy.	Only positive samples included
19	Brini, 2018	Temporal and climate characteristics of respiratory syncytial virus bronchiolitis in neonates and children in Sousse, Tunisia, during a 13-year surveillance	No molecular assays used
20	Brini, 2020	Temporal and climate characteristics of respiratory syncytial virus bronchiolitis in neonates and children in Sousse, Tunisia, during a 13-year surveillance.	Inappropriate detection assay
21	Calvo, 2015	Respiratory Syncytial Virus Coinfections With Rhinovirus and Human Bocavirus in Hospitalized Children	> 2 years
22	Cantani, 1999	Bronchiolitis in infants.	No data on viral etiology searched
23	Causse, 2007	Preliminary evaluation of a multiplex reverse transcription-PCR assay combined with a new DNA chip hybridization assay for detecting respiratory syncytial virus	> 2 years
24	Cavallin, 2013	Infection with multiple viruses is not associated with increased disease severity in children with bronchiolitis.	Comments
25	Centers for Disease Control and Prevention (CDC), 2003	Bronchiolitis-associated outpatient visits and hospitalizations among American Indian and Alaska Native children--United States, 1990-2000.	Report
26	Chan, 2002	Risk factors for hypoxemia and respiratory failure in respiratory syncytial virus bronchiolitis.	Only positive samples included
27	Chee, 2010	Emergency Department Septic Screening in Respiratory Syncytial Virus (RSV) and Non-RSV Bronchiolitis.	No molecular assays used
28	Chen, 2004	[Clinical characteristics of bronchiolitis caused by human metapneumovirus in infants]	Full text not found
29	Chen, 2014	Viral etiology of bronchiolitis among pediatric inpatients in northern Taiwan with emphasis on newly identified respiratory viruses.	Combination of multiple asays for detection
30	Ciarlitto, 2019	Respiratory Syncityal Virus A and B: three bronchiolitis seasons in a third level hospital in Italy.	Inappropriate study population
31	Connors, 2016	Viral Bronchiolitis in Children.	Comments
32	Coskun, 2017	Risk factors for intensive care need in children with bronchiolitis: A case-control study.	Data on detection assay not reported
33	Côté, 2003	Comparative evaluation of real-time PCR assays for detection of the human metapneumovirus	No data on viral etiology searched
34	CRONE, 1964	SEROLOGICAL EVIDENCE OF INFECTION BY RESPIRATORY SYNCYTIAL VIRUS IN OUTBREAK OF ACUTE BRONCHIOLITIS	No molecular assays used
35	Cubie, 1992	Detection of respiratory syncytial virus in acute bronchiolitis in infants	Not possible to extract data on viral etiology searched

36	Dagan, 1993	Hospitalization of Jewish and Bedouin infants in southern Israel for bronchiolitis caused by respiratory syncytial virus	No molecular assays used
37	Davies, 2017	A systematic review of the psychometric properties of bronchiolitis assessment tools.	Systematic review
38	DeVincenzo, 2016	Viral Bronchiolitis in Children.	Comments
39	Dotan, 2013	Hospitalization for respiratory syncytial virus bronchiolitis and disease severity in twins.	Only positive samples included
40	Duttweiler, 2004	Pulmonary and systemic bacterial co-infections in severe RSV bronchiolitis.	Only positive samples included
41	Eidelman, 2009	The burden of respiratory syncytial virus bronchiolitis on a pediatric inpatient service.	No molecular assays used
42	Erculj, 2018	PM10 exposure is associated with increased hospitalizations for respiratory syncytial virus bronchiolitis among infants in Lombardy, Italy.	Only positive samples included
43	Erez, 2012	[Prevalence of H1N1 A influenza virus infection among hospitalized patients with bronchiolitis twelve months old and younger]	Full text not found
44	Eugene-Ruellan, 1998	Detection of respiratory syncytial virus A and B and parainfluenzavirus 3 sequences in respiratory tracts of infants by a single PCR with primers targeted to the L-polymerase gene and differential hybridization	Age range not reported
45	FANDRE, 1964	[EPIDEMIC OF ACUTE BRONCHIOLITIS IN INFANTS. ISOLATION OF A RESPIRATORY SYNEYTIAL VIRUS]	Full text not found
46	Fauroux, 2017	The Burden and Long-term Respiratory Morbidity Associated with Respiratory Syncytial Virus Infection in Early Childhood.	Systematic review
47	Fedele, 2018	Analysis of the immune response in infants hospitalized with viral bronchiolitis shows different Th1/Th2 profiles associated with respiratory syncytial virus and human rhinovirus.	Inappropriate study population
48	Ferronato, 2012	Etiological diagnosis reduces the use of antibiotics in infants with bronchiolitis.	No molecular assays used
49	Fjaerli, 2004	Hospitalisations for respiratory syncytial virus bronchiolitis in Akershus, Norway, 1993-2000: a population-based retrospective study.	No molecular assays used
50	Flaherman, 2010	Respiratory syncytial virus testing during bronchiolitis episodes of care in an integrated health care delivery system: a retrospective cohort study.	No molecular assays used
51	Flamant, 2005	Severe respiratory syncytial virus bronchiolitis in children: from short mechanical ventilation to extracorporeal membrane oxygenation.	No molecular assays used
52	Flores, 2004	Bronchiolitis caused by respiratory syncytial virus in an area of portugal: epidemiology, clinical features, and risk factors.	> 2 years
53	Fodha, 2007	Respiratory syncytial virus infections in hospitalized infants: association between viral load, virus subgroup, and disease severity	Only positive samples included

54	Foley, 2018	Respiratory health inequality starts early: The impact of social determinants on the aetiology and severity of bronchiolitis in infancy.	No molecular assays used
55	Foo, 1991	Severe bronchiolitis in children	Full text not found
56	FORBES, 1961	Epidemic bronchiolitis caused by a respiratory syncytial virus: clinical aspects	Conference abstract
57	Fretzayas, 2017	Etiology and clinical features of viral bronchiolitis in infancy.	Review
58	Freymouth, 2003	Presence of the new human metapneumovirus in French children with bronchiolitis.	Age range not reported
59	Fujiogi, 2020	Association of rhinovirus species with nasopharyngeal metabolome in bronchiolitis infants: A multicenter study.	Duplicates
60	Ganu, 2012	Increase in use of non-invasive ventilation for infants with severe bronchiolitis is associated with decline in intubation rates over a decade	Data on detection assay not reported
61	Garcia, 2010	Risk factors in children hospitalized with RSV bronchiolitis versus non-RSV bronchiolitis.	No molecular assays used
62	Garcia-Marcos, 2014	Pediatricians' attitudes and costs of bronchiolitis in the emergency department: a prospective multicentre study.	No data on viral etiology searched
63	Ghazaly, 2018	Characteristics of children admitted to intensive care with acute bronchiolitis.	Combination of multiple asays for detection
64	Ghazaly, 2018	Characteristics of children admitted to intensive care with acute bronchiolitis.	Inappropriate detection assay
65	Giordano, 2018	Respiratory syncytial virus bronchiolitis and hypertransaminasemia.	Case report
66	Gold, 2006	[Respiratory syncytial virus bronchiolitis: severe respiratory forms in hospitalized infants]	Review
67	González Martínez, 2013	[Clinical impact of introducing ventilation with high flow oxygen in the treatment of bronchiolitis in a paediatric ward]	No molecular assays used
68	Goto-Sugai, 2010	Genotyping and phylogenetic analysis of the major genes in respiratory syncytial virus isolated from infants with bronchiolitis.	Only positive samples included
69	Greensill, 2003	Human metapneumovirus in severe respiratory syncytial virus bronchiolitis.	Age range not reported
70	Grimaldi, 2002	[Prospective regional study of an epidemic of respiratory syncytial virus	Only positive samples included
71	Grimwood, 2008	Risk factors for respiratory syncytial virus bronchiolitis hospital admission in New Zealand.	Combination of multiple asays for detection
72	Grubbauer, 1989	[Respiratory insufficiency in acute bronchiolitis in infancy]	Full text not found
73	Haque, 2012	Bronchiolitis outbreak caused by respiratory syncytial virus in southwest Bangladesh, 2010.	Outbreak
74	Hasegawa, 2014	Multicenter study of viral etiology and relapse in hospitalized children with bronchiolitis.	Duplicate of Dumas, 2016
75	Hasegawa, 2014	Infectious pathogens and bronchiolitis outcomes.	Review
76	Hasegawa, 2015	Risk factors for requiring intensive care among children admitted to ward with bronchiolitis.	Duplicate of Dumas, 2016

77	Hasegawa, 2015	Respiratory syncytial virus genomic load and disease severity among children hospitalized with bronchiolitis: multicenter cohort studies in the United States and Finland.	Duplicate of Dumas, 2016
78	Hasegawa, 2018	Rhinovirus Species in Children with Severe Bronchiolitis: Multicenter Cohort Studies in the US and Finland.	Duplicate of Dumas, 2016
79	Hasegawa, 2019	Respiratory Virus Epidemiology Among US Infants With Severe Bronchiolitis: Analysis of 2 Multicenter, Multiyear Cohort Studies.	Duplicates
80	Hasegawa, 2019	Association of Rhinovirus C Bronchiolitis and Immunoglobulin E Sensitization During Infancy With Development of Recurrent Wheeze.	Duplicates
81	Heinonen, 2018	Transient Tachypnea of the Newborn is Associated with an Increased Risk of Hospitalization Due to RSV Bronchiolitis.	Data on detection assay not reported
82	Hendaus, 2014	Does cesarean section pose a risk of respiratory syncytial virus bronchiolitis in infants and children?	> 2 years
83	Henderson, 1979	The etiologic and epidemiologic spectrum of bronchiolitis in pediatric practice	No molecular assays used
84	Hervas, 2012	Epidemiology of hospitalization for acute bronchiolitis in children: differences between RSV and non-RSV bronchiolitis.	No molecular assays used
85	Houben, 2011	Clinical prediction rule for RSV bronchiolitis in healthy newborns: prognostic birth cohort study	Not bronchiolitis
86	Howidi, 2007	The severity of respiratory syncytial virus bronchiolitis in young infants in the United Arab Emirates.	No molecular assays used
87	Huguenin, 2012	Broad respiratory virus detection in infants hospitalized for bronchiolitis by use of a multiplex RT-PCR DNA microarray system.	Age range not reported
88	Hyvarinen, 2011	Outcome after bronchiolitis depends on disease definition.	Only positive samples included
89	Jacques, 2006	Association of respiratory picornaviruses with acute bronchiolitis in French infants.	Duplicate of Janahi, 2017
90	Jacques, 2008	Human Bocavirus quantitative DNA detection in French children hospitalized for acute bronchiolitis.	> 2 years
91	Jartti, 2015	Rhinovirus-induced bronchiolitis: Lack of association between virus genomic load and short-term outcomes	Duplicate of Dumas, 2016
92	Jelić, 1990	[A bronchiolitis epidemic caused by respiratory syncytial viruses]	Full text not found
93	Jevsnik, 2016	The Role of Human Coronaviruses in Children Hospitalized for Acute Bronchiolitis, Acute Gastroenteritis, and Febrile Seizures: A 2-Year Prospective Study.	> 2 years
94	Jhawar, 2003	Severe bronchiolitis in children.	Review
95	Kabir, 2003	Evaluation of hospitalized infants and young children with bronchiolitis-a multi centre study.	Full text not found
96	Karr, 2009	Infant exposure to fine particulate matter and traffic and risk of hospitalization for RSV	No data on viral etiology searched

		bronchiolitis in a region with lower ambient air pollution.	
97	Kassis, 2009	[The burden and outcomes of acute bronchiolitis among young children hospitalized in Israel]	Full text not found
98	Kemper, 2005	Hospital readmission for bronchiolitis.	No data on viral etiology searched
99	Korppi, 2012	Upper age limit for bronchiolitis: 12 months or 6 months?	Comments
100	Korppi, 2015	Bronchiolitis: the disease of <6-month-old, <12-month-old or <24-month-old infants.	Comments
101	Kua, 2017	Systematic Review and Meta-Analysis of the Efficacy and Safety of Combined Epinephrine and Corticosteroid Therapy for Acute Bronchiolitis in Infants.	Systematic review
102	Laham, 2017	Clinical Profiles of Respiratory Syncytial Virus Subtypes A AND B Among Children Hospitalized with Bronchiolitis.	Duplicate of Dumas, 2016
103	Lanari, 2015	Prenatal tobacco smoke exposure increases hospitalizations for bronchiolitis in infants.	No data on viral etiology searched
104	Lanari, 2015	Risk factors for bronchiolitis hospitalization during the first year of life in a multicenter Italian birth cohort.	No data on viral etiology searched
105	Lanari, 2016	Exposure to vehicular traffic is associated to a higher risk of hospitalization for bronchiolitis during the first year of life.	Full text not found
106	Legg, 2005	Frequency of detection of picornaviruses and seven other respiratory pathogens in infants	Not bronchiolitis
107	Lin, 2009	[Detection of human metapneumovirus and human bocavirus in children with bronchiolitis in east Guangdong area]	Full text not found
108	López-Huertas, 2005	Two RT-PCR based assays to detect human metapneumovirus in nasopharyngeal aspirates	Not bronchiolitis
109	Lowther, 2000	Bronchiolitis-associated hospitalizations among American Indian and Alaska Native children.	No data on viral etiology searched
110	Luo, 2014	A systematic review of predictive modeling for bronchiolitis.	Systematic review
111	Mação, 2011	[Acute bronchiolitis: a prospective study]	Data on detection assay not reported
112	Macfarlane, 2005	RSV testing in bronchiolitis: which nasal sampling method is best?	No molecular assays used
113	Mandal, 2017	Bronchiolitis: Comparative Study between Respiratory Synctial Virus	Comments
114	Mandal, 2017	Bronchiolitis: Comparative Study between Respiratory Synctial Virus (RSV) and Non RSV Aetiology.	Comments
115	Mansbach, 2008	Prospective multicenter study of the viral etiology of bronchiolitis in the emergency department	Not possible to extract data on viral etiology searched
116	Mansbach, 2012	Prospective multicenter study of viral etiology and hospital length of stay in children with severe bronchiolitis	Duplicate of Dumas, 2016
117	Mansbach, 2016	Children Hospitalized with Rhinovirus Bronchiolitis Have Asthma-Like Characteristics.	Duplicate of Dumas, 2016

118	Mansbach, 2016	Respiratory syncytial virus and rhinovirus severe bronchiolitis are associated with distinct nasopharyngeal microbiota.	Comments
119	Mansbach, 2018	Haemophilus-dominant nasopharyngeal microbiota is associated with delayed clearance of respiratory syncytial virus in infants hospitalized for bronchiolitis	Duplicate of Mansbach, 2016
120	Mansbach, 2019	Association between rhinovirus species and nasopharyngeal microbiota in infants with severe bronchiolitis.	Duplicates
121	Mansbach, 2020	Detection of respiratory syncytial virus or rhinovirus weeks after hospitalization for bronchiolitis and the risk of recurrent wheezing.	Duplicates
122	Marguet, 2009	In very young infants severity of acute bronchiolitis depends on carried viruses.	Combination of multiple asays for detection
123	McCuskee, 2014	Bronchiolitis and pneumonia requiring hospitalization in young first nations children in Northern Ontario, Canada.	Not possible to extract data on viral etiology searched
124	McErlean, 2007	Characterisation of a newly identified human rhinovirus, HRV-QPM, discovered in infants with bronchiolitis.	> 2 years
125	McNally, 2014	Vitamin D receptor (VDR) polymorphisms and severe RSV bronchiolitis: a systematic review and meta-analysis.	Systematic review
126	McNamara, 2007	Impact of human metapneumovirus and respiratory syncytial virus co-infection in severe bronchiolitis.	Age range not reported
127	Meissner, 2016	Viral Bronchiolitis in Children.	Comments
128	Meissner, 2016	More on Viral Bronchiolitis in Children.	Comments
129	Meissner, 2016	Viral Bronchiolitis in Children.	Review
130	Mikalsen, 2012	The outcome after severe bronchiolitis is related to gender and virus.	Only positive samples included
131	Miron, 2010	Sole pathogen in acute bronchiolitis: is there a role for other organisms apart from respiratory syncytial virus?	Combination of multiple asays for detection
132	Molinari Such, 2005	Respiratory syncytial virus-related bronchiolitis in Puerto Rico.	Only positive samples included
133	Munoz-Quiles, 2016	Population-based Analysis of Bronchiolitis Epidemiology in Valencia, Spain.	No data on viral etiology searched
134	Murray, 2014	Risk factors for hospital admission with RSV bronchiolitis in England: a population-based birth cohort study.	No data on viral etiology searched
135	Naja, 2019	Bronchiolitis Admissions in a Lebanese Tertiary Medical Center: A 10 Years' Experience.	Inappropriate study population
136	Najioullah, 2020	Seasonality and coinfection of bronchiolitis: epidemiological specificity and consequences in terms of prophylaxis in tropical climate.	Inappropriate detection assay
137	Narbona-Lopez, 2018	Prevention of syncytial respiratory virus infection with palivizumab: descriptive and comparative analysis after 12 years of use.	Full text not found

138	Nenna, 2017	Modifiable risk factors associated with bronchiolitis.	No data on viral etiology searched
139	Neves Barreira, 2001	[Relationship between respiratory syncytial virus subtype and clinical severity in bronchiolitis]	Full text not found
140	Nicolai, 2013	Viral bronchiolitis in children: a common condition with few therapeutic options.	Review
141	O'Connor, 2013	The changing epidemiology of the bronchiolitis epidemic in Tallaght Hospital.	Full text not found
142	Oliveira-Santos, 2016	Influence of meteorological conditions on RSV infection in Portugal	No molecular assays used
143	Ong, 2001	A comparison of nested polymerase chain reaction and immunofluorescence for the diagnosis of respiratory infections in children with bronchiolitis, and the implications for a cohorting strategy	No molecular assays used
144	Ozkaya-Parlakay, 2019	Viral Etiology of Bronchiolitis Among Pediatric Patients.	Inappropriate study design
145	Papadopoulos, 2004	Does respiratory syncytial virus subtype influences the severity of acute bronchiolitis in hospitalized infants?	Only positive samples included
146	Paranhos-Baccalà, 2008	Mixed respiratory virus infections	Review
147	Paul, 2017	Respiratory-syncytial-virus- and rhinovirus-related bronchiolitis in children aged <2 years in an English district general hospital.	Not possible to extract data on viral etiology searched
148	Perrin, 1986	[Predictive indicators of the severity of bronchiolitis caused by respiratory syncytial virus in infants]	Full text not found
149	Pichler, 2000	Severe adenovirus bronchiolitis in children.	Case report
150	Pickles, 2015	Respiratory syncytial virus (RSV) and its propensity for causing bronchiolitis.	Review
151	Piedimonte, 2014	Respiratory syncytial virus infection and bronchiolitis.	Review
152	Prais, 2003	Admission to the intensive care unit for respiratory syncytial virus bronchiolitis: a national survey before palivizumab use.	No molecular assays used
153	Pruikkonen, 2014	Infants under 6 months with bronchiolitis are most likely to need major medical interventions in the 5 days after onset.	No molecular assays used
154	Rahbarimanesh, 2018	Viral Aetiology of Bronchiolitis in Hospitalised Children in a Tertiary Center in Tehran.	No molecular assays used
155	Rahbarimanesh, 2018	Viral Aetiology of Bronchiolitis in Hospitalised Children in a Tertiary Center in Tehran.	Inappropriate detection assay
156	Ralston, 2009	Incidence of apnea in infants hospitalized with respiratory syncytial virus bronchiolitis: a systematic review.	Systematic review
157	Ramagopal, 2016	Demographic, Clinical and Hematological Profile of Children with Bronchiolitis: A Comparative Study between Respiratory Synctial Virus [RSV] and [Non RSV] Groups.	> 2 years
158	Rivera-Sepulveda, 2017	Epidemiology of bronchiolitis: a description of emergency department visits and hospitalizations in Puerto Rico, 2010-2014.	No data on viral etiology searched

159	Rodl, 2012	Prospective evaluation of clinical scoring systems in infants with bronchiolitis admitted to the intensive care unit.	No data on viral etiology searched
160	Rodriguez-Fernandez, 2017	Respiratory Syncytial Virus Genotypes, Host Immune Profiles, and Disease Severity in Young Children Hospitalized With Bronchiolitis.	Only positive samples included
161	Rodriguez-Martinez, 2018	Predictors of prolonged length of hospital stay for infants with bronchiolitis.	No molecular assays used
162	Rossi, 2016	Viral Bronchiolitis in Children.	Comments
163	Ryu, 2015	Etiology and Outcome of Diffuse Acute Infectious Bronchiolitis in Adults.	Combination of multiple asays for detection
164	Saijo, 1994	The role of respiratory syncytial virus in acute bronchiolitis in small children in northern Japan	No molecular assays used
165	Sala, 2015	Factors associated with disease severity in children with bronchiolitis.	No data on viral etiology searched
166	Sanchez-Luna, 2016	Trends in respiratory syncytial virus bronchiolitis hospitalizations in children less than 1 year: 2004-2012.	No data on viral etiology searched
167	Sarkar, 2018	Comparative Study between Noninvasive Continuous Positive Airway Pressure and Hot Humidified High-flow Nasal Cannulae as a Mode of Respiratory Support in Infants with Acute Bronchiolitis in Pediatric Intensive Care Unit of a Tertiary Care Hospital.	No data on viral etiology searched
168	Scagnolari, 2012	Evaluation of viral load in infants hospitalized with bronchiolitis caused by respiratory syncytial virus.	Only positive samples included
169	Schaller, 2017	Bronchiolitis in Infants and Children.	Review
170	Semple, 2005	Dual infection of infants by human metapneumovirus and human respiratory syncytial virus is strongly associated with severe bronchiolitis.	Age range not reported
171	Semple, 2011	Household tobacco smoke and admission weight predict severe bronchiolitis in infants independent of deprivation: prospective cohort study.	No molecular assays used
172	Shadman, 2012	50 years ago in The Journal of Pediatrics: Observations on the etiology of acute bronchiolitis in infants.	Review
173	Shang, 2014	Elective cesarean delivery as a predisposing factor of respiratory syncytial virus bronchiolitis in children	> 2 years
174	Shay, 2001	Bronchiolitis-associated mortality and estimates of respiratory syncytial virus-associated deaths among US children, 1979-1997.	No data on viral etiology searched
175	Shmueli, 2017	Real-life comparison of three general paediatric wards showed similar outcomes for children with bronchiolitis despite different treatment regimens.	No molecular assays used
176	Simon, 2007	Detection of bocavirus DNA in nasopharyngeal aspirates of a child with bronchiolitis.	Case report
177	Sloan, 2013	Spatiotemporal patterns of infant bronchiolitis in a Tennessee Medicaid population.	No data on viral etiology searched
178	Smyth, 2002	Respiratory syncytial virus bronchiolitis: disease severity, interleukin-8, and virus genotype.	Only positive samples included

179	Soo, 2017	Pulmonary hemorrhage as a complication of Respiratory Syncyntial Virus (RSV) bronchiolitis.	Case report
180	Stempel, 2009	Multiple viral respiratory pathogens in children with bronchiolitis.	No data on viral etiology searched
181	Stevenson, 2016	Prenatal Versus Postnatal Tobacco Smoke Exposure and Intensive Care Use in Children Hospitalized With Bronchiolitis.	Duplicate of Dumas, 2016
182	Stewart, 2019	Association of respiratory viruses with serum metabolome in infants with severe bronchiolitis.	Duplicates
183	Stollar, 2014	Virologic testing in bronchiolitis: does it change management decisions and predict outcomes?	Combination of multiple assays for detection
184	Tecu, 2006	The viral bronchiolites diagnosis in children by PCR multiplex	Full text not found
185	Tecu, 2012	The adenoviral infections in children admitted to hospital with pneumonia, acute bronchiolitis or respiratory viral infections.	Full text not found
186	Toivonen, 2019	Association between rhinovirus species and nasopharyngeal microbiota in infants with severe bronchiolitis	Duplicate of Mansbach, 2016
187	Tortora, 2015	Adenovirus species C detection in children under four years of age with acute bronchiolitis or recurrent wheezing.	> 2 years
188	Toyoshima, 2011	Bronchiolitis caused by pandemic influenza A (H1N1) 2009.	Case report
189	Tsolia, 2003	Epidemiology of respiratory syncytial virus bronchiolitis in hospitalized infants in Greece.	No molecular assays used
190	Tumba, 2020	Temporal trend of hospitalizations for acute bronchiolitis in infants under one year of age in brazil between 2008 and 2015.	No data on respiratory viruses prevalence
191	Valdivia, 1997	Analysis of respiratory syncytial virus in clinical samples by reverse transcriptase-polymerase chain reaction restriction mapping	Age range not reported
192	Van Rostenberghe, 2006	RSV and bronchiolitis.	Comments
193	van Woensel, 2002	Bronchiolitis hospitalisations in the Netherlands from 1991 to 1999.	No data on viral etiology searched
194	Vicente, 1978	[Etiologic importance of the respiratory syncytial virus in bronchiolitis]	Full text not found
195	Wall, 2016	Viral Bronchiolitis in Children.	Comments
196	Werno, 2004	Human metapneumovirus in children with bronchiolitis or pneumonia in New Zealand.	> 2 years
197	Willson, 2003	Complications in infants hospitalized for bronchiolitis or respiratory syncytial virus pneumonia.	Data on detection assay not reported
198	Wolfler, 2018	The infant with severe bronchiolitis: from HFNC to CPAP and mechanical ventilation.	Review
199	Wright, 2002	Illness severity, viral shedding, and antibody responses in infants hospitalized with bronchiolitis caused by respiratory syncytial virus.	Only positive samples included
200	Yitshak-Sade, 2017	Air Pollution and Hospitalization for Bronchiolitis among Young Children.	No data on viral etiology searched

201	Yi-Wei, 2018	A molecular epidemiological study of respiratory syncytial virus circulating in southern Zhejiang Province, China, from 2009 to 2014.	Inappropriate detection assay
202	Yorita, 2007	Severe bronchiolitis and respiratory syncytial virus among young children in Hawaii.	Data on detection assay not reported
203	Zhang, 2017	[Molecular biological and clinical characteristics of respiratory syncytial virus in children with bronchiolitis]	Full text not found

S5 Table. Individual characteristics of included studies

Name, Year	Male (%)	Age	Age_Range	Period	Design	Sampling	Timing	Country	Language	Bias	Setting	Case definition	Sample_type	Virus_Searched	Viral_diagnostic_method
Amat, 2014	59	4.05 months ± 2.79	< 1 Year	Oct/2011-May/2012	Cross sectional	Consecutive	Prospective	France	English	Moderate risk	Outpatients	Bronchiolitis was diagnosed by the presence of a recent history of an upper respiratory tract infection followed by onset of respiratory distress with cough, tachypnea, retraction and diffuse crackles, or wheezing on auscultation, in accordance with the international recommendations on the diagnosis and management of acute bronchiolitis [Subcommittee on diagnosis and management of bronchiolitis, 2006].	Nasopharyngeal secretions	HRSV; HMPV; RV; HAdV; HBoV; HPIV; HCoV; EV	Real-time PCR
Antunes, 2010	Not reported	Not reported	< 2 Years	Nov/2007-Apr/2008	Cross sectional	Consecutive	Prospective	Portugal	English	Moderate risk	Hospitalized	The same AAP definition was adopted by all hospitals.	Nasopharyngeal secretions	HRSV; HMPV; Influenza; RV; HAdV; HBoV; HPIV; HCoV; EV	Real-time PCR
Arabzadeh, 2008	Not reported	Not reported	< 2 Years	2006	Cross sectional	Consecutive	Prospective	Iran	Non english	Moderate risk	Not reported	Not reported	Nasopharyngeal secretions	HRSV; HAdV; HPIV	PCR
Atay, 2020	59.4	9±2.4 months	< 2 Years	Sep/2011-Apr/2012	Cross sectional	Consecutive sampling	Prospectively	Turkey	English	Moderate risk of bias	Hospitalized/Outpatients	The diagnosis of bronchiolitis was based primarily on each patient's history of preceding viral upper respiratory tract infections, restlessness, cough and/or rhinorrhea with signs of a respiratory illness including tachypnoea, intercostal and/or subcostal retractions, accessory muscle use, nasal flaring, grunting, color changes or apnea, wheezing or crackles, and lower oxygen saturations [7].	Nasopharyngeal secretions	HAdV; HCoV; HMPV; HPIV; HRSV; Influenza; RV	Multiplex PCR
Azkur, 2014	63.6	6.98 ± 5.53 months	< 2 Years	Nov/2011-May/2012	Cross sectional	Consecutive	Prospective	Turkey	English	Low risk	Hospitalized	Bronchiolitis is defined as a constellation of clinical symptoms and signs including a viral upper respiratory prodrome followed by increased respiratory effort and wheezing in children less than 2 years of age4.	Nasopharyngeal secretions	HRSV; HMPV; Influenza; RV; HAdV; HBoV; HPIV; HCoV; EV	Real-time multiplex PCR
Bamberger, 2012	62	17.5 ± 16.6 weeks	< 2 Years	Dec/2005-Mar/2006	Cross sectional	Consecutive	Prospective	Israel	English	Low risk	Hospitalized	Acute AB was diagnosed clinically in those subjects with upper respiratory tract infections (URTIs) with cough and by at least two of the following signs: chest retractions, tachypnea, wheezing, or rales on auscultation.	Nasopharyngeal secretions	HRSV; HMPV; Influenza; RV; HAdV; HBoV; HPIV	PCR
Bekhof, 2013	54,2	3.2 months	< 2 Years	Dec/2011-Mar/2012	Cross sectional	Consecutive	Prospective	Netherlands	English	Low risk	Hospitalized	Bronchiolitis was defined as acute respiratory disease, accompanied by coryza, cough, inspiratory crackles and/or expiratory wheezing on auscultation.	Nasopharyngeal secretions	HRSV; HMPV; RV; HAdV; HCoV	PCR
Bekhof, 2019	59	4.9 (1.6–7.0) mo	< 2 Years	Dec-Mar; 2012-2016	Cohort (Baseline data)	Consecutive sampling	Prospectively	Netherlands	English	Moderate risk of bias	Hospitalized	Bronchiolitis was defined as acute respiratory disease accompanied by coryza, cough, and inspiratory crackles and/or expiratory wheezing on auscultation.	Nasopharyngeal secretions	EV; HAdV; HCoV; HMPV; HPIV; HRSV; Influenza; RV	Real-time PCR
Brand, 2012	61	4.5 ± 0.39 (months)	< 2 Years	2006-2009 (Nov-Apr)	Cross sectional	Consecutive	Prospective	Pays-Bas	English	Low risk	Hospitalized/Outpatients	Bronchiolitis was defined as an acute infection of the lower airways, characterized by increased respiratory effort (tachypnea and/or use of accessory respiratory muscles) and expiratory wheezing and/or crackles and/or apnea.	Nasopharyngeal secretions	HRSV; HMPV; Influenza; RV; HAdV; HBoV; HPIV; HCoV; EV	PCR
Calvo, 2010	55	5.6 ± 5.7 months	< 2 Years	Sept/2005-Aug/2008	Cross sectional	Consecutive	Prospective	Spain	English	Low risk	Hospitalized	Acute bronchiolitis, was diagnosed as the first episode of acute onset expiratory dyspnea with previous signs of viral respiratory infection – whether or not this was associated to respiratory distress or pneumonia in children under 2 years old.	Nasopharyngeal secretions	HRSV; HMPV; Influenza; RV; HAdV; HBoV; HPIV; HCoV; EV	PCR
Cangiano, 2016	54.6	Median age 2.13 months	< 1 Year	2004-2014 (Oct-May)	Cross sectional	Consecutive	Prospective	Italy	English	Low risk	Hospitalized	Acute bronchiolitis was clinically defined as the first episode of acute lower respiratory tract infection in infants less than 12 months, characterized by the acute onset of cough, tachypnoea, retraction, and diffuse crackles on chest auscultation.9	Nasopharyngeal secretions	HRSV; HMPV; Influenza; RV; HBoV; HPIV; HCoV	PCR
Chen, 2014	67.3	Mean 255.6 days; 95% CI 240.4-270.9 days	< 2 Years	Jan/2009-Dec/2010	Cross sectional	Consecutive	Prospective	China	English	Low risk	Hospitalized	Acute bronchiolitis was defined as the first episode of cough, rhinorrhea, wheezing, or rales, with chest radiographic findings of over-aeration and peribronchial infiltration with or without atelectasis in children younger than 2 years.	Nasopharyngeal secretions	HMPV; HBoV	PCR
Dong-Keon, 2018	Unclear/Not repor	2.8 ± 2.4 months	< 1 Year	Nov/2016-Feb/2017	Cross sectional	Consecutive sampling	Retrospectively	North Korea	Non english	Moderate risk of bias	Hospitalized	Unclear/Not reported	Nasopharyngeal secretions	HRSV	Real-time PCR
Dumas, 2016	61	8.1 months (SD: 6.7)	< 2 Years	2007-2010 (Nov-Mar)	Cross sectional	Consecutive	Prospective	Finlande	English	Low risk	Hospitalized	Bronchiolitis (as defined by the American Academy of Pediatrics:28 acute respiratory illness with some combination of rhinitis, cough, tachypnoea, wheezing, crackles and retractions), and were younger than 2 years.	Nasopharyngeal secretions	HRSV; RV	PCR
Dumas, 2016	59	5.8 months (SD: 5.2)	< 2 Years	2007-2010 (Nov-Mar)	Cross sectional	Consecutive	Prospective	USA	English	Low risk	Hospitalized	Bronchiolitis (as defined by the American Academy of Pediatrics:28 acute respiratory illness with some combination of rhinitis, cough, tachypnoea, wheezing, crackles and retractions), and were younger than 2 years.	Nasopharyngeal secretions	HRSV; RV	PCR
Ebihara, 2005	Not reported	Not reported	< 2 Years	Oct/2002-Sep/2003	Cross sectional	Consecutive	Prospective	Japan	English	Moderate risk	Hospitalized	Not reported	Nasopharyngeal secretions	HCoV	PCR
Gokce, 2017	63.6	7 ± 6.5 months	< 2 Years	Oct/2013-Oct/2016	Cross sectional	Consecutive	Prospective	Turkey	English	Low risk	Hospitalized	The diagnosis of AB was based on at least 2 of the following signs: chest retractions, tachypnea, and wheezing or rales on auscultation following viral upper respiratory tract infection in children less than 24 months of age for the first time.11	Nasal secretions	HRSV; HMPV; Influenza; RV; HAdV; HBoV; HPIV; HCoV	RT-PCR
Hendaus, 2018	60.8	3.61 ± 3.56 months	< 2 Years	Jan/2010-Dec/2012	Cross sectional	Consecutive	Retrospective	Qatar	English	Moderate risk	Hospitalized	The diagnosis of bronchiolitis was based on the definition suggested by the American Academy of pediatrics, subcommittee on diagnosis and management of bronchiolitis.1	Nasopharyngeal secretions	HRSV; RV; HAdV; HBoV; HPIV; HCoV	Real-time PCR
Janahi, 2017	67	Not reported	< 2 Years	2010-2011	Cross sectional	Consecutive	Retrospective	Qatar	English	Low risk	Hospitalized	Acute bronchiolitis defined according to International Classification of Disease (ICD) code 466.1 in the 9th revision of the ICD. Based on recorded clinical diagnosis, an episode of acute bronchiolitis was determined by a constellation of clinical signs and symptoms including fever, rhinitis, tachypnoea, cough, wheezing, crackles, use of accessory muscles and possible chest X ray findings of hyperinflation of the lungs, peri-bronchial thickening, collapsed segment or a lobe of the lung and increase interstitial markings.	Nasopharyngeal secretions	HRSV; HMPV; Influenza; RV; HAdV; HBoV; HPIV; HCoV; EV	Real-time PCR
Jartti, 2014	61	8.1 months (SD: 6.7)	< 2 Years	2007-2010 (Nov-Mar)	Cross sectional	Consecutive	Prospective	Finlande	English	Low risk	Hospitalized	Bronchiolitis (as defined by the American Academy of Pediatrics:28 acute respiratory illness with some combination of rhinitis, cough, tachypnoea, wheezing, crackles and retractions), and were younger than 2 years.	Nasopharyngeal secretions	HMPV; Influenza; RV; HAdV; HPIV; HCoV; EV	PCR
Mansbach, 2008	61	Median age of 6.3 months (IQR 3.1 to 10.2)	< 2 Years	Dec/2005-Mar/2006	Cohort	Consecutive	Prospective	USA	English	Moderate risk	Outpatients	The diagnosis of bronchiolitis was considered for children younger than 2 years with acute tachypnea, retractions, and abnormal breath sounds who did not meet criteria for a diagnosis of pneumonia or another primary diagnosis. As defined by the AAP in its 2006 position statement, children with bronchiolitis typically have ''rhinitis, tachypnea, wheezing, cough, crackles, use of accessory muscles, and ? or nasal flaring.''12	Nasopharyngeal secretions	HRSV; RV	RT-PCR
Mansbach, 2016	Not reported	Not reported	< 1 Year	2011-2014 (Nov-Apr)	Cohort	Consecutive	Prospective	USA	English	Low risk	Hospitalized	Bronchiolitis defined by the Academy of Pediatrics: acute respiratory illness with some combination of rhinitis, cough, tachypnea, wheezing, crackles, and retractions;1	Nasopharyngeal secretions	HRSV; RV	PCR
Midulla, 2010	57	2.5 ± 2.1 months	< 1 Year	2004-2007 (Oct-May)	Cross sectional	Consecutive	Prospective	Italy	English	Low risk	Hospitalized	Bronchiolitis was diagnosed according to the presence of a history of upper respiratory tract infection followed by acute onset of respiratory distress with cough, tachypnea, retraction and diffuse crackles on auscultation (having wheezing alone was not considered sufficient for inclusion in the study).18	Nasal secretions	HRSV; HMPV; Influenza; RV; HBoV; HPIV; HCoV	PCR
Midulla, 2018	Not reported	Not reported	< 1 Year	2005-2017 (Sep-May)	Cross sectional	Consecutive	Prospective	Italy	English	Low risk	Hospitalized	Bronchiolitis was defined as the first episode of acute lower respiratory tract infection in infants less than 1 year old, characterized by the acute onset of cough, tachypnoea, retraction, and diffuse crackles (wheezing alone was insufficient for inclusion) [28].	Nasopharyngeal secretions	HRSV	PCR
Miller, 2013	Not reported	Not reported	< 1 Year	2004-2008 (Sept-May)	Cohort	Consecutive	Prospective	USA	English	Low risk	Hospitalized/Outpatients	Bronchiolitis was defined as lower respiratory infection in infants presenting with wheezing, rales, and respiratory distress.	Nasopharyngeal secretions	HRSV; HMPV; Influenza; RV; HPIV; HCoV	PCR
Nascimento, 2010	65	Median age was 6 ± 4 months	< 2 Years	Mar/2006-Jul/2007	Cross sectional	Consecutive	Prospective	Brazil	English	Low risk	Outpatients	Bronchiolitis was defined as the first episode of wheezing associated with signs of an upper respiratory tract infection.	Nasopharyngeal secretions	HRSV; HMPV; Influenza; RV; HAdV; HBoV; HPIV; HCoV; EV	PCR, RT-PCR, Real-time RT-PCR
Nenna, 2020	Unclear/Not repor	Unclear/Not reported	< 1 Year	Unclear/Not reported	Cross sectional	Consecutive sampling	Prospectively	Italy	English	Moderate risk of bias	Hospitalized	Bronchiolitis was defined as the first episode of acute lower airway infection, characterized by a history of upper respiratory tract infection followed by acute onset of respiratory distress with cough, tachypnoea, retraction, and diffuse crackles on auscultation.	Nasopharyngeal secretions	HBoV; HMPV; HRSV; RV	Classical PCR
Nenna, 2020	Unclear/Not repor	Unclear/Not reported	< 1 Year	Unclear/Not reported	Cross sectional	Consecutive sampling	Prospectively	Italy	English	Moderate risk of bias	Hospitalized	Bronchiolitis was defined as the first episode of acute lower airway infection, characterized by a history of upper respiratory tract infection followed by acute onset of respiratory distress with cough, tachypnoea, retraction, and diffuse crackles on auscultation.	Nasopharyngeal secretions	HBoV; HMPV; HRSV; RV	Classical RT-PCR
Papadopoulos, 2002	61.3	3.9 months	< 1.5 years	Oct/1999-Sep/2000	Cross sectional	Consecutive	Prospective	Greece	English	Low risk	Hospitalized	Bronchiolitis was defined as an acute infection of the lower airway characterized by increased respiratory effort (tachypnea [more than 50 respirations/minute] and/or use of accessory respiratory muscles) and expiratory wheezing and/or crackles.	Nasopharyngeal secretions	HRSV; Influenza; RV; HAdV; HPIV; HCoV	PCR
Papoff, 2011	50.5	Median (interquartile. I.Q.) were 61 days (I.Q. 33–99 days)	< 1 Year	2004-2009 (Oct-May)	Cross sectional	Consecutive	Prospective	Italy	English	Low risk	Hospitalized	Bronchiolitis was diagnosed clinically according to the presence of a history of upper respiratory tract infection followed by acute onset of respiratory distress with cough, tachypnea, retractions and bilateral crackles on auscultation having wheezing alone was not considered sufficient for inclusion in the study (11).	Nasal secretions	HRSV; HMPV; Influenza; RV; HBoV; HPIV; HCoV	PCR
Petrarca, 2018	54.1	Median age 2.03 months. range: 0.23-11.17	< 1 Year	2004-2016 (Oct-May)	Cross sectional	Consecutive	Retrospective	Italy	English	Low risk	Hospitalized	Bronchiolitiswas clinically defined as the first episode of acute lower respiratory tract infection, characterized by the acute onset of cough, tachypnea, retraction, and diffuse crackles on chest auscultation in infants younger than 12 months.13	Nasopharyngeal secretions	HRSV; RV	PCR
Piedra, 2017	60.8	6.7±6.3 months	< 2 Years	Oct/2010-Apr/2011	Cross sectional	Consecutive	Prospective	USA	English	Low risk	Outpatients	Bronchiolitis was defined as a physician diagnosis in children less than 24 months of age with wheezing and/or rales who had a history of preceding upper airway illness.	Nasopharyngeal secretions	HRSV	Real-time PCR
Pitrez, 2005	56	Median 2 (1.2-3.6) months	< 0.5 year	May/2012-Sep/2012	Cohort	Consecutive	Prospective	Brazil	Non english	Moderate risk	Hospitalized	BA was defined as the first episode of wheezing, associated with signs and symptoms of acute viral infection (coryza and cough).	Nasopharyngeal secretions	RV	RT-PCR
Praznik, 2018	61,5	Not reported	< 2 Years	May/2014-Oct/2008	Cross sectional	Consecutive	Retrospective	Slovenia	English	Moderate risk	Hospitalized/Outpatients	Patients were identified through a query of the electronic medical record based on a bronchiolitis diagnosis (ICD-10 code J21.0-9).	Nasopharyngeal secretions	HRSV; RV; HBoV	RT-PCR
Ricart, 2013	Not reported	Not reported	< 1 Year	Sep/2007-Oct/2008	Cross sectional	Consecutive	Prospective	Spain	English	Low risk	Hospitalized	Patients were admitted if one of the following criteria was present: pulse oximetry <92% on room air, moderate bronchiolitis (according to bronchiolitis clinical score),5 poor feeding with risk of dehydration, apnea, significant congenital heart disease or pulmonary bronchodysplasia, or infant aged <4 weeks.	Nasopharyngeal secretions	HRSV; HMPV; Influenza; RV; HAdV; HBoV; HPIV; HCoV; EV	PCR
Ricart, 2013	Not reported	Not reported	< 1 Year	Oct/2007-Oct/2008	Cross sectional	Consecutive	Prospective	Spain	English	Low risk	Hospitalized	Bronchiolitis was diagnosed according to the presence of the first acute respiratory tract infection characterized by respiratory distress (tachypnea, use of accessory muscles), cough, widespread crackles, wheezing or both, associated with signs of viral infection (coryza).2	Nasopharyngeal secretions	HRSV; HMPV; Influenza; RV; HAdV; HBoV; HPIV; HCoV; EV	RT-PCR, Real-time RT-PCR
Ricart, 2014	60.8	41.7 (13-70.3) (Mean (95% confidence interval) days)	< 1 Year	Jan/2006-Oct/2008	Cross sectional	Consecutive	Prospective	Spain	English	Low risk	Hospitalized	Bronchiolitis was diagnosed according to the presence of the first acute respiratory tract infection characterized by respiratory distress, cough, widespread crackles or wheezing or both, associated with signs of viral infection (coryza).	Nasopharyngeal secretions	HRSV; HMPV; RV; HAdV; HCoV	PCR
Richard, 2008	Not reported	Not reported	< 1 Year	2003-2005 (Sep-Apr)	Cross sectional	Consecutive	Retrospective	France	English	Moderate risk	Hospitalized	Not reported	Nasopharyngeal secretions	HRSV; Influenza; RV; HAdV; HPIV; EV	PCR ou NASBA
Robledo-Aceves, 2018	60.5	6.60 ± 5.7 months	< 2 Years	Sep/2012-Jan/2015	Case-control	Consecutive	Retrospective	Mexico	English	Low risk	Hospitalized	Severe bronchiolitis was defined as rhinorrhea, cough, tachypnea, wheezing, rales, and increased respiratory effort (e.g., grunting, nasal flaring, and intercostal and/or subcostal retraction), with symptoms of severity (e.g., increased respiratory rate, retractions, and oxygen saturation at 90% or lower).[25]	Nasopharyngeal secretions	HRSV; HMPV; Influenza; RV; HBoV; HPIV; HCoV	RT-PCR, PCR
Salvador García, 2012	53.6	3.4 (± 3.3) months	< 1.5 years	Dec/2008-Apr-2009	Cross sectional	Consecutive	Prospective	Spain	Non english	Low risk	Hospitalized	The diagnosis of bronchiolitis was established according to the McConnochie criteria7, defined as the first acute episode of respiratory distress with wheezing associated with catarrhal pathology in children under 2 years of age.	Nasopharyngeal secretions	HRSV; HMPV; Influenza; RV; HAdV; HBoV; HPIV; HCoV; EV	RT-PCR
Souza, 2016	Not reported	Not reported	< 1 Year	Sep/2010-Aug/2011	Cross sectional	Consecutive	Prospective	Brazil	English	Low risk	Hospitalized	Acute bronchiolitis (based on prodromal symptoms, with wheezing, crackles, and tachypnea); having been recruited within the first 48 h of hospitalization; and having a ? 72-h history of clinical manifestations of lower respiratory tract infection (wheeze or respiratory distress).	Nasopharyngeal secretions	HRSV; HMPV; RV	Real-time PCR
Stein, 2019	53.5	4 months	< 2 Years	2016–2018	Cross sectional	Consecutive sampling	Prospectively	Greece	English	Moderate risk of bias	Hospitalized	Unclear/Not reported	Nasopharyngeal secretions	HRSV	Classical RT-PCR
Su-Jin, 2018	62.6	Unclear/Not reported	< 2 Years	Nov/2007-May/2015	Cross sectional	Consecutive sampling	Prospectively	South Korea	Non english	Low risk of bias	Hospitalized	Bronchiolitis was defined as a clinical syndrome accompanied by symptoms of upper respiratory tract infection and lower respiratory tract infection such as wheezing.	Nasopharyngeal secretions	EV; HAdV; HBoV; HCoV; HMPV; HPIV; HRSV; Influenza; RV	Real-time PCR
Teeratakulpisarn, 2007	Not reported	Not reported	< 2 Years	Apr/2002-Aug/2004	Cross sectional	Consecutive	Prospective	Thailand	English	Low risk	Hospitalized	Bronchiolitis was defined as the first epi-sode of wheezing associated with tachypnea, in-creased respiratory effort and an upper respiratory tract infection.	Nasopharyngeal secretions	HRSV; HMPV	RT-PCR
Tsou, 2020	57.4	Unclear/Not reported	< 1 Year	Jan/2015-Dec/2017	Case control	Simple random sampling	Prospectively	United States of America	English	Low risk of bias	Hospitalized	The study sample was chosen based on the ICD code for bronchiolitis during the study period.	Nasopharyngeal secretions	HAdV; HCoV; HMPV; HPIV; HRSV	Real-time PCR
Uyar, 2014	72.5	7.18 ± 5.75 months	< 2 Years	Jan/2010-Jun/2010	Cross sectional	Consecutive	Prospective	Turkey	Non english	Low risk	Outpatients	Diagnosis of acute bronchiolitis; upper respiratory tract infect nasal discharge, cough, mild fever, agitation difficulty eating, wheezing, increased respiratory rate, chest weaning was performed according to the criteria of expiration time and wheezing.	Nasopharyngeal secretions	HRSV; HMPV; Influenza; RV; HAdV; HBoV; HPIV; EV	Multiplex PCR
Vieira, 2017	48	2.4 months (SD = 1.6)	< 0.5 year	2013-2015	Cross sectional	Consecutive	Prospective	Brazil	English	Moderate risk	Hospitalized	Diagnosis of bronchiolitis was defined as the first wheezing crisis, beginning no more than 3 days before hospital admission.	Nasal secretions	HRSV	Real-time PCR
Wang, 2015	68,4	Not reported	< 2 Years	Jan/2010-Dec/2013	Cross sectional	Consecutive	Prospective	China	English	Low risk	Hospitalized	The bronchiolitis was diagnosed based on the guidelines described by Zhu Futang Practical Pediatrics, 7th Edition (12).	Nasal secretions	HMPV	RT-PCR, Real-time RT-PCR
Wollmeister, 2018	Unclear/Not repor	Unclear/Not reported	< 1 Year	2004; 2014	Cross sectional	Consecutive sampling	Prospectively	Brazil	English	Moderate risk of bias	Hospitalized	Diagnosis was based on clinical data, which defines AVB as being the first episode of acute respiratory distress with wheezing, preceded by upper airway symptoms such as rhinorrhea and cough, with or without fever [1,5]. The criterion for severe bronchiolitis was oxygen saturation lower than 92%, which demanded oxygen therapy [1].	Nasopharyngeal secretions	HAdV; HBoV; HCoV; HMPV; HPIV; HRSV; Influenza; RV	Classical PCR
Xepapadaki, 2004	Not reported	Not reported	< 1.5 years	Oct/1999-Sep/2000	Cross sectional	Consecutive	Prospective	Greece	English	Low risk	Hospitalized	Bronchiolitis, which was defined as an acute infection of the lower airway, characterized by increased respiratory effort (tachypnea of >50 respirations/min and/or use of accessory respiratory muscles), and expiratory wheezing and/or crackles.	Nasopharyngeal secretions	HMPV	PCR
Xuan, 2018	Unclear/Not repor	Unclear/Not reported	Unclear/Not reported	Jan/2015-Dec/2016	Cross sectional	Consecutive sampling	Prospectively	China	Non english	Moderate risk of bias	Unclear/Not reported	Unclear/Not reported	Throat secretions	HBoV; HRSV	Classical PCR
Zhou, 2015	Not reported	Not reported	< 2 Years	2012-2014 (winter)	Cross sectional	Consecutive	Prospective	China	English	Low risk	Hospitalized	The diagnosis of bronchiolitis is according to the Zhu Futang textbook of Pediatrics [Zhang, 2002]. Bronchiolitis was diagnosed from the presence of a history of upper respiratory tract infection followed by acute onset of respiratory distress with cough, tachypnea, retraction, wheezing, and diffuse crackles on auscultation.	Nasopharyngeal secretions	HRSV	PCR

S6 Table. Global prevalence and sensitivity analyses of respiratory viral infections in children < 2 years with bronchiolitis

	Prevalence [95% CI]	95% Prediction interval	N Studies	N Cases	H [95% CI]	I2 [95% CI]	P-Value Heterogeneity	P-Value Egger test
**HRSV**								
Overall	59.2 [54.7-63.6]	[29.1-85.9]	45	15351	5.5 [5.1-6]	96.8 [96.2-97.2]	< 0.001	0.415
< 1 Year	62.3 [55.7-68.6]	[32.4-87.8]	18	6779	5.4 [4.7-6.1]	96.5 [95.5-97.3]	< 0.001	0.878
Hospitalized	60.6 [55.9-65.2]	[32.8-85.2]	34	12867	5.3 [4.8-5.8]	96.4 [95.7-97]	< 0.001	0.601
Bronchiolitis definition given	60.4 [56-64.8]	[32.4-85.2]	40	14456	5.3 [4.8-5.7]	96.4 [95.7-97]	< 0.001	0.473
Cross sectional design	57.2 [52.3-62]	[27.2-84.5]	39	12992	5.4 [5-5.9]	96.6 [96-97.2]	< 0.001	0.395
Low risk of bias	59.4 [54.1-64.5]	[30.4-85.2]	30	11024	5.4 [4.9-5.9]	96.5 [95.8-97.2]	< 0.001	0.325
**RV**								
Overall	19.3 [16.7-22]	[6.3-37]	36	12967	3.7 [3.3-4.2]	92.8 [90.9-94.2]	< 0.001	0.318
< 1 Year	16.9 [13.4-20.7]	[4.8-34]	15	5382	3.4 [2.8-4.1]	91.5 [87.6-94.1]	< 0.001	0.856
Hospitalized	18 [15.1-21.1]	[5-36.4]	28	11159	4 [3.5-4.5]	93.8 [92-95.1]	< 0.001	0.142
Bronchiolitis definition given	19.2 [16.6-22]	[6.2-37.2]	35	12787	3.8 [3.4-4.2]	93 [91.2-94.4]	< 0.001	0.315
Cross sectional design	19.9 [16.9-23.2]	[5.9-39.4]	30	10833	4 [3.5-4.5]	93.6 [91.9-95]	< 0.001	0.315
Low risk of bias	21 [18-24.2]	[8-37.9]	25	9414	3.5 [3-4]	91.6 [88.9-93.7]	< 0.001	0.304
**HBoV**								
Overall	8.2 [5.6-11.2]	[0-27.3]	24	8706	4.7 [4.1-5.3]	95.4 [94.1-96.4]	< 0.001	0.748
< 1 Year	8 [2.4-16.6]	[0-49]	8	3104	7.3 [6.2-8.6]	98.1 [97.4-98.7]	< 0.001	0.864

	Prevalence [95% CI]	95% Prediction interval	N Studies	N Cases	H [95% CI]	I2 [95% CI]	P-Value Heterogeneity	P-Value Egger test
Hospitalized	8 [5-11.6]	[0-28.6]	18	7335	5.2 [4.5-5.9]	96.2 [95.1-97.1]	< 0.001	0.853
Bronchiolitis definition given	8.2 [5.5-11.3]	[0-27.8]	23	8310	4.8 [4.2-5.4]	95.6 [94.4-96.6]	< 0.001	0.753
Cross sectional design	8.3 [5.6-11.4]	[0-27.6]	23	8572	4.8 [4.2-5.4]	95.6 [94.4-96.6]	< 0.001	0.777
Low risk of bias	9.5 [6.5-13.1]	[0.3-28.2]	17	5578	4.1 [3.5-4.8]	94 [91.8-95.6]	< 0.001	0.331
**HAdV**								
Overall	6.1 [4.4-8]	[0.2-18.1]	26	6734	3 [2.6-3.5]	88.9 [85-91.8]	< 0.001	0.085
< 1 Year	6.7 [3.6-10.6]	[0-23.1]	7	1938	3 [2.2-4]	88.6 [78.9-93.8]	< 0.001	0.538
Hospitalized	6.1 [4.2-8.4]	[0.1-18.9]	20	5963	3.3 [2.8-3.9]	90.6 [87-93.3]	< 0.001	0.121
Bronchiolitis definition given	6.2 [4.4-8.2]	[0.2-18.2]	24	6386	3 [2.6-3.5]	89 [84.9-92]	< 0.001	0.097
Cross sectional design	6.4 [4.5-8.6]	[0.2-19.2]	23	6112	3.1 [2.6-3.6]	89.5 [85.6-92.4]	< 0.001	0.149
Low risk of bias	6.4 [4.2-8.9]	[0.1-20]	18	4632	3.1 [2.6-3.7]	89.7 [85.2-92.8]	< 0.001	0.052
**HPIV**								
Overall	5.4 [3.8-7.3]	[0-17.9]	28	7933	3.3 [2.9-3.8]	91 [88.1-93.2]	< 0.001	0.932
< 1 Year	3.1 [1.9-4.6]	[0.1-9.3]	10	3185	2.1 [1.6-2.9]	78.1 [60.1-88]	< 0.001	0.531
Hospitalized	5 [3.3-6.9]	[0-16.7]	21	6707	3.4 [2.9-3.9]	91.1 [87.8-93.5]	< 0.001	0.238
Bronchiolitis definition given	5 [3.4-6.8]	[0-16.7]	26	7585	3.3 [2.8-3.8]	90.6 [87.4-92.9]	< 0.001	0.682
Cross sectional design	5.3 [3.6-7.2]	[0-17.2]	24	6856	3.2 [2.8-3.7]	90.3 [86.8-92.8]	< 0.001	0.528

	Prevalence [95% CI]	95% Prediction interval	N Studies	N Cases	H [95% CI]	I2 [95% CI]	P-Value Heterogeneity	P-Value Egger test
Low risk of bias	5 [3.1-7.2]	[0-18]	20	5831	3.4 [2.9-4]	91.5 [88.3-93.9]	< 0.001	0.983
**HMPV**								
Overall	5.4 [4.4-6.4]	[1.4-11.5]	32	9908	2.1 [1.8-2.5]	76.9 [67.7-83.5]	< 0.001	0.043
< 1 Year	3.4 [2.4-4.7]	[0.5-8.4]	12	3936	1.9 [1.4-2.5]	71.4 [48.6-84.1]	< 0.001	0.633
Hospitalized	5.1 [4.2-6.1]	[1.9-9.7]	26	8850	1.8 [1.5-2.2]	68.8 [53.2-79.1]	< 0.001	0.238
Bronchiolitis definition given	5.4 [4.4-6.4]	[1.4-11.5]	32	9908	2.1 [1.8-2.5]	76.9 [67.7-83.5]	< 0.001	0.043
Cross sectional design	5.6 [4.6-6.7]	[1.9-11.1]	28	8831	1.9 [1.6-2.3]	72.7 [60.3-81.2]	< 0.001	0.04
Low risk of bias	5.7 [4.5-7]	[1.3-12.8]	26	8034	2.2 [1.9-2.7]	79.6 [70.8-85.8]	< 0.001	0.07
**Influenza**								
Overall	3.2 [2.2-4.3]	[0-10]	24	6571	2.4 [2-2.8]	82.1 [74.3-87.5]	< 0.001	0.032
< 1 Year	1.6 [0.8-2.6]	[0-5.2]	8	2694	1.7 [1.2-2.5]	65.4 [26.3-83.7]	0.005	0.422
Hospitalized	2.6 [1.8-3.6]	[0.1-7.8]	19	5734	2.1 [1.7-2.6]	77.4 [65.1-85.4]	< 0.001	0.407
Bronchiolitis definition given	3.2 [2.1-4.4]	[0-10.2]	23	6391	2.4 [2-2.9]	82.8 [75.3-88.1]	< 0.001	0.037
Cross sectional design	3 [2-4.3]	[0-10.1]	21	5764	2.4 [2-2.9]	82.8 [74.8-88.3]	< 0.001	0.083
Low risk of bias	3.2 [2.1-4.6]	[0-10.6]	19	5561	2.5 [2.1-3.1]	84.2 [76.7-89.4]	< 0.001	0.08
**HCoV**								
Overall	2.9 [2-4]	[0-9.9]	27	7431	2.5 [2.1-2.9]	83.5 [76.9-88.1]	< 0.001	0.918
< 1 Year	2.6 [1-4.8]	[0-13.3]	10	3056	3.1 [2.4-4]	89.7 [83.2-93.7]	< 0.001	0.744

	Prevalence [95% CI]	95% Prediction interval	N Studies	N Cases	H [95% CI]	I2 [95% CI]	P-Value Heterogeneity	P-Value Egger test
Hospitalized	2.6 [1.6-3.8]	[0-9.4]	22	6435	2.5 [2.1-3]	84 [76.9-88.9]	< 0.001	0.556
Bronchiolitis definition given	2.9 [2-4.1]	[0-10]	26	7313	2.5 [2.1-3]	84.1 [77.7-88.6]	< 0.001	0.931
Cross sectional design	2.6 [1.7-3.7]	[0-8.8]	23	6354	2.3 [1.9-2.8]	81.1 [72.5-87]	< 0.001	0.744
Low risk of bias	2.8 [1.8-4]	[0-9.4]	20	5559	2.3 [1.9-2.8]	81.5 [72.3-87.6]	< 0.001	0.701
**EV**								
Overall	2.9 [1.6-4.5]	[0-11.4]	15	4202	2.7 [2.2-3.3]	86.1 [78.6-90.9]	< 0.001	0.886
< 1 Year	2.2 [1.4-3]	[0.7-4.3]	4	1303	1 [1-2.3]	0 [0-80.5]	0.502	0.292
Hospitalized	2.2 [1-3.6]	[0-9.1]	11	3700	2.6 [2-3.3]	84.7 [74.3-90.9]	< 0.001	0.115
Bronchiolitis definition given	2.8 [1.4-4.6]	[0-11.7]	14	4022	2.8 [2.2-3.5]	87 [79.9-91.6]	< 0.001	0.849
Cross sectional design	2.9 [1.5-4.7]	[0-12]	14	3984	2.8 [2.2-3.5]	87 [79.9-91.6]	< 0.001	0.911
Low risk of bias	3.4 [1.7-5.6]	[0-13.5]	11	3386	2.8 [2.2-3.6]	87.7 [79.9-92.5]	< 0.001	0.863

CI: confidence interval; RV: Rhinovirus; HCoV: Human Coronavirus; HPIV: Human Parainfluenzavirus; HMPV: Human Metapneumovirus; HRSV: Human Respiratory Syncytial Virus; HAdV: Human Adenovirus; HBoV: Human Bocavirus; EV: Enterovirus; NA: not applicable.

S7 Table. Subgroup prevalence of respiratory viral infections in children with acute bronchiolitis

	**Prevalence [95% CI]**	**95% Prediction interval**	**H [95% CI]**	**N Studies**	**N Cases**	**I2 [95% CI]**	**P-Value Heterogeneity**	**P-Value Egger test**	**P-value subgroup difference**
**HRSV**									
**Study period**									0.304
Continuous study period	57.3 [51.6-62.9]	[26.1-85.6]	4.9 [4.4-5.4]	30	7454	95.8 [94.9-96.6]	< 0.001	0.647	
Interrupted time series study	62.4 [54.3-70.2]	[28.7-90.5]	6.6 [5.8-7.5]	14	7073	97.7 [97-98.2]	< 0.001	0.622	
**WHO Region**									< 0.001
America	64 [58.2-69.6]	[42.8-82.7]	3.5 [2.8-4.4]	10	4731	92 [87.4-94.9]	< 0.001	0.104	
Eastern Mediterranean	46.9 [40.2-53.7]	[0-100]	2.2 [1.3-4]	3	1240	80 [36.8-93.7]	0.007	0.386	
Europe	59 [52.9-64.9]	[27.4-86.9]	5.3 [4.7-5.9]	26	7636	96.4 [95.6-97.1]	< 0.001	0.815	
Northern America	82.6 [77.8-86.9]	NA	NA	1	270	NA	1	NA	
South-East Asia	64.7 [57.3-71.7]	NA	NA	1	170	NA	1	NA	
Western Pacific	51.3 [32.1-70.3]	[0-100]	6.1 [4.6-8.1]	4	1304	97.3 [95.3-98.5]	< 0.001	0.705	
**Sample type**									< 0.001
Nasal secretions	50 [36.5-63.5]	[1.6-98.4]	4 [2.8-5.8]	4	902	93.9 [87.4-97]	< 0.001	0.116	
Nasopharyngeal secretions	61 [56.8-65.2]	[34-84.9]	5 [4.6-5.5]	40	14053	96 [95.2-96.6]	< 0.001	0.465	
Throat secretions	24.5 [20.4-28.9]	NA	NA	1	396	NA	1	NA	
**RV**									
**Study period**									0.391
Continuous study period	20.4 [16.9-24]	[6.3-39.6]	3.3 [2.8-3.8]	24	6199	90.8 [87.7-93.2]	< 0.001	0.314	
Interrupted time series study	18 [13.7-22.7]	[4.5-37.8]	4.2 [3.5-5.1]	11	5944	94.4 [91.8-96.2]	< 0.001	0.178	
**WHO Region**									< 0.001
America	17.8 [13.6-22.4]	[5.5-34.9]	3.2 [2.5-4.2]	9	4570	90.5 [84.2-94.3]	< 0.001	0.187	
Eastern Mediterranean	18.8 [8.3-32.3]	NA	4.9 [2.9-8.3]	2	1072	95.9 [88.4-98.6]	< 0.001	NA	

	Prevalence [95% CI]	95% Prediction interval	H [95% CI]	N Studies	N Cases	I2 [95% CI]	P-Value Heterogeneity	P-Value Egger test	P-value subgroup difference
Europe	19.3 [15.9-23]	[5.1-39.5]	3.6 [3.1-4.1]	24	6557	92.2 [89.7-94.1]	< 0.001	0.905	
Western Pacific	33.5 [30.2-36.8]	NA	NA	1	768	NA	1	NA	
**Sample type**									0.243
Nasal secretions	12.7 [3.9-25.4]	[0-100]	4.7 [3.1-6.9]	3	808	95.4 [89.9-97.9]	< 0.001	0.762	
Nasopharyngeal secretions	20 [17.3-22.8]	[7.2-37]	3.6 [3.2-4]	33	12159	92.2 [90.1-93.9]	< 0.001	0.441	
**HBoV**									
**Study period**									0.09
Continuous study period	9.4 [6.4-12.9]	[0.1-29.1]	4.5 [4-5.2]	19	6897	95.2 [93.6-96.3]	< 0.001	0.497	
Interrupted time series study	5.1 [2.2-9.1]	[0-30.4]	2.4 [1.5-3.9]	4	985	82.9 [56.4-93.3]	0.001	0.386	
**WHO Region**									< 0.001
America	4.7 [0-15.2]	[0-100]	3.8 [2.4-5.9]	3	525	93.1 [83.1-97.2]	< 0.001	0.11	
Eastern Mediterranean	4 [2.9-5.3]	NA	1	2	1072	0	0.913	NA	
Europe	9 [5.4-13.5]	[0-33.4]	4.9 [4.3-5.7]	16	4947	95.9 [94.5-96.9]	< 0.001	0.884	
Western Pacific	11.6 [10.3-13]	[4.3-21.7]	1 [1-2.9]	3	2162	0 [0-88.3]	0.411	0.47	
**Sample type**									0.545
Nasal secretions	6.6 [2.7-12]	[0-97]	2.6 [1.5-4.5]	3	808	85.6 [57.9-95.1]	0.001	0.344	
Nasopharyngeal secretions	8.4 [5.4-12]	[0-29.4]	5 [4.4-5.7]	20	7502	96 [94.9-96.9]	< 0.001	0.784	
Throat secretions	9.8 [7.1-13]	NA	NA	1	396	NA	1	NA	
**HAdV**									
**Study period**									0.081
Continuous study period	6.6 [4.7-8.8]	[0.3-18.8]	3 [2.5-3.5]	22	5786	88.6 [84.1-91.8]	< 0.001	0.081	
Interrupted time series study	3.7 [1.6-6.5]	[0-20.2]	1.9 [1.1-3.2]	4	948	72 [20.7-90.1]	0.013	0.501	
**WHO Region**									< 0.001
America	1.5 [0.4-3.2]	[0-31]	1.2 [1-3.6]	3	525	24.9 [0-92.2]	0.264	0.454	

	Prevalence [95% CI]	95% Prediction interval	H [95% CI]	N Studies	N Cases	I2 [95% CI]	P-Value Heterogeneity	P-Value Egger test	P-value subgroup difference
Eastern Mediterranean	6.9 [4.6-9.6]	[0-51.6]	1.6 [1-3]	3	1240	61.8 [0-89.1]	0.073	0.177	
Europe	6.3 [4.5-8.4]	[0.7-16.4]	2.3 [1.9-2.9]	18	3931	81.8 [72.2-88]	< 0.001	0.543	
Northern America	5.9 [3.4-9.1]	NA	NA	1	270	NA	1	NA	
Western Pacific	18.1 [15.5-20.9]	NA	NA	1	768	NA	1	NA	
**Sample type**									0.532
Nasal secretions	7.3 [4.6-10.4]	NA	NA	1	316	NA	1	NA	
Nasopharyngeal secretions	6 [4.2-8.1]	[0.1-18.5]	3.1 [2.6-3.6]	25	6418	89.3 [85.5-92.1]	< 0.001	0.092	
**HPIV**									
**Study period**									0.005
Continuous study period	6.7 [4.6-9.1]	[0.1-20.5]	3.3 [2.8-3.9]	20	5687	91 [87.5-93.5]	< 0.001	0.943	
Interrupted time series study	2.8 [1.4-4.6]	[0-10.3]	2.2 [1.6-3.1]	8	2246	79.3 [59.6-89.4]	< 0.001	0.622	
**WHO Region**									< 0.001
America	6 [0.8-15]	[0-67.2]	4.4 [3.1-6.2]	4	980	94.8 [89.8-97.4]	< 0.001	0.166	
Eastern Mediterranean	11.6 [6.8-17.4]	[0-95.4]	2.7 [1.6-4.6]	3	1240	86.4 [60.6-95.3]	0.001	0.424	
Europe	3.9 [2.7-5.3]	[0.3-10.8]	2.1 [1.7-2.7]	19	4675	78.3 [66.7-85.9]	< 0.001	0.283	
Northern America	8.1 [5.2-11.7]	NA	NA	1	270	NA	1	NA	
Western Pacific	13.7 [11.3-16.2]	NA	NA	1	768	NA	1	NA	
**Sample type**									0.086
Nasal secretions	2.8 [0.8-5.9]	[0-76.1]	2.1 [1.2-3.8]	3	808	78.1 [29.5-93.2]	0.01	0.718	
Nasopharyngeal secretions	5.8 [4-7.9]	[0-19]	3.4 [2.9-3.9]	25	7125	91.4 [88.5-93.5]	< 0.001	0.969	
**HMPV**									
**Study period**									0.005
Continuous study period	6.3 [5.2-7.4]	[2.6-11.2]	1.7 [1.3-2.1]	24	7018	64.3 [44.8-76.9]	< 0.001	0.012	
Interrupted time series study	3.1 [1.5-5.1]	[0-11.8]	2.3 [1.6-3.3]	7	2066	81.4 [62.6-90.8]	< 0.001	0.735	

	Prevalence [95% CI]	95% Prediction interval	H [95% CI]	N Studies	N Cases	I2 [95% CI]	P-Value Heterogeneity	P-Value Egger test	P-value subgroup difference
**WHO Region**									0.443
America	5.4 [1.6-11.1]	[0-33.5]	3 [2.1-4.4]	5	1036	89 [77-94.7]	< 0.001	0.117	
Eastern Mediterranean	6.2 [4-8.9]	NA	NA	1	369	NA	1	NA	
Europe	5.7 [4.4-7.2]	[1.2-12.8]	2 [1.7-2.5]	21	5355	76.1 [63.6-84.3]	< 0.001	0.017	
Northern America	3.3 [1.5-5.9]	NA	NA	1	270	NA	1	NA	
South-East Asia	3.5 [1.2-6.9]	NA	NA	1	170	NA	1	NA	
Western Pacific	5.5 [4.5-6.7]	[0-20.3]	1.3 [1-2.3]	3	2708	38.6 [0-80.8]	0.196	0.692	
**Sample type**									0.198
Nasal secretions	3.7 [1.4-7]	[0-25.9]	2.8 [1.8-4.4]	4	1750	87.6 [70.5-94.8]	< 0.001	0.655	
Nasopharyngeal secretions	5.7 [4.6-6.8]	[1.6-11.8]	2 [1.7-2.4]	28	8158	74.7 [63.5-82.5]	< 0.001	0.017	
**Influenza**									
**Study period**									0.014
Continuous study period	4 [2.6-5.8]	[0-12.8]	2.5 [2-3.1]	16	4325	84.4 [76.1-89.9]	< 0.001	0.055	
Interrupted time series study	1.9 [1-3]	[0-5.9]	1.6 [1.1-2.4]	8	2246	62.8 [20-82.7]	0.009	0.313	
**WHO Region**									0.058
America	2.8 [0.5-6.4]	[0-28]	2.5 [1.6-4]	4	980	83.9 [59.4-93.6]	< 0.001	0.426	
Eastern Mediterranean	1.1 [0.2-2.5]	NA	NA	1	369	NA	1	NA	
Europe	3.5 [2.2-5]	[0-11.7]	2.4 [2-3]	18	4454	83.3 [74.8-88.9]	< 0.001	0.034	
Western Pacific	3.3 [2.1-4.6]	NA	NA	1	768	NA	1	NA	
**Sample type**									0.677
Nasal secretions	2.2 [0-9.2]	[0-100]	4.5 [3-6.7]	3	808	95 [88.8-97.8]	< 0.001	0.74	
Nasopharyngeal secretions	3.2 [2.3-4.4]	[0.2-9.1]	2.1 [1.7-2.6]	21	5763	77.3 [65.7-85]	< 0.001	0.006	
**HCoV**									
**Study period**									0.589
Continuous study period	3.1 [2-4.4]	[0-9.8]	2.3 [1.9-2.8]	20	5365	80.9 [71.3-87.2]	< 0.001	0.789	

	**Prevalence [95% CI]**	**95% Prediction interval**	**H [95% CI]**	**N Studies**	**N Cases**	**I2 [95% CI]**	**P-Value Heterogeneity**	**P-Value Egger test**	**P-value subgroup difference**
Interrupted time series study	2.5 [0.8-5.1]	[0-14.6]	3 [2.2-4]	7	2066	88.7 [79.2-93.9]	< 0.001	0.717	
**WHO Region**									< 0.001
America	1.5 [0.8-2.4]	[0.2-3.7]	1 [1-2]	4	980	0 [0-75.5]	0.598	0.625	
Eastern Mediterranean	4.2 [1.4-8.4]	NA	2.7 [1.4-5.4]	2	1072	86.5 [46.7-96.6]	0.006	NA	
Europe	2.6 [1.5-4.1]	[0-10.4]	2.4 [2-3]	18	4223	83 [74.3-88.8]	< 0.001	0.639	
Northern America	10.7 [7.3-14.7]	NA	NA	1	270	NA	1	NA	
Western Pacific	4.4 [2.4-7]	NA	1.3 NA	2	886	37.7 NA	0.205	NA	
**Sample type**									0.071
Nasal secretions	1.1 [0-3.3]	[0-70]	2.1 [1.2-3.8]	3	808	77.7 [27.9-93.1]	0.011	0.79	
Nasopharyngeal secretions	3.2 [2.2-4.4]	[0-10.3]	2.4 [2-2.9]	24	6623	82.8 [75.5-88]	< 0.001	0.969	
**EV**									
**Study period**									0.466
Continuous study period	2.6 [1-4.7]	[0-13.3]	3.1 [2.4-3.9]	11	3254	89.4 [83-93.4]	< 0.001	0.716	
Interrupted time series study	3.6 [2.1-5.5]	[0-12.1]	1.3 [1-2.3]	4	948	44.1 [0-81.3]	0.147	0.304	
**WHO Region**									< 0.001
America	20.8 [12.4-30.6]	NA	NA	1	77	NA	1	NA	
Eastern Mediterranean	1.6 [0.5-3.2]	NA	NA	1	369	NA	1	NA	
Europe	2 [1.2-2.9]	[0.2-5.2]	1.5 [1.1-2.1]	12	2988	55.3 [14.5-76.6]	0.01	0.632	
Western Pacific	7.8 [6-9.8]	NA	NA	1	768	NA	1	NA	
**Sample type**									NA
Nasopharyngeal secretions	2.9 [1.6-4.5]	[0-11.4]	2.7 [2.2-3.3]	15	4202	86.1 [78.6-90.9]	< 0.001	0.886	

CI: confidence interval; RV: Rhinovirus; HCoV: Human Coronavirus; HPIV: Human Parainfluenzavirus; HMPV: Human Metapneumovirus; HRSV: Human Respiratory Syncytial Virus; HAdV: Human Adenovirus; HBoV: Human Bocavirus; EV: Enterovirus; NA: not applicable.

S1 Fig. Global prevalence of Respiratory Viruses in children < 2 years with bronchiolitis

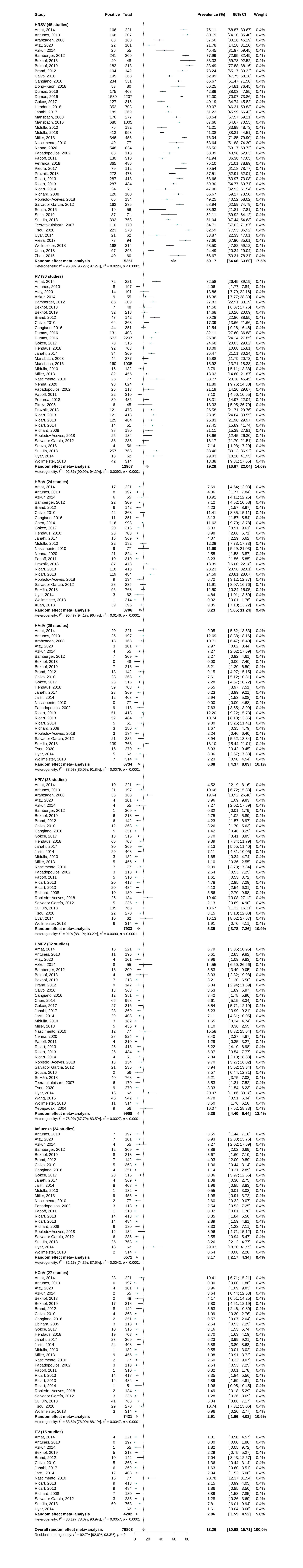

S2 Fig. Codetection rate of viral infections among children < 2 years with bronchiolitis

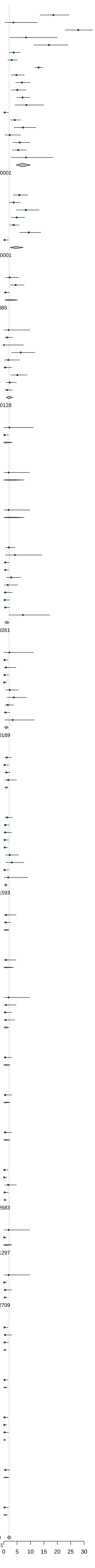

Study	Positive	Total	Prevalence (%)	95% CI	Weight
**HRSV + RV (20 studies)**					
Amat, 2014	41	221	18.55	[13.65; 24.31]	1.0%
Azkur, 2014	2	55	3.64	[0.44; 12.53]	0.8%
Bamberger, 2012	86	309	27.83	[22.91; 33.19]	1.0%
Bekhof, 2013	4	48	8.33	[2.32; 19.98]	0.7%
Brand, 2012	24	142	16.90	[11.14; 24.10]	0.9%
Cangiano, 2016	13	351	3.70	[1.99; 6.25]	1.0%
Dumas, 2016	12	408	2.94	[1.53; 5.08]	1.0%
Dumas, 2016	287	2207	13.00	[11.63; 14.48]	1.1%
Gokce, 2017	15	316	4.75	[2.68; 7.71]	1.0%
Janahi, 2017	25	369	6.78	[4.43; 9.84]	1.0%
Mansbach, 2008	14	277	5.05	[2.79; 8.33]	1.0%
Miller, 2013	32	455	7.03	[4.86; 9.78]	1.0%
Papadopoulos, 2002	10	118	8.47	[4.14; 15.03]	0.9%
Papoff, 2011	1	310	0.32	[0.01; 1.78]	1.0%
Petrarca, 2018	20	486	4.12	[2.53; 6.28]	1.0%
Richard, 2008	13	180	7.22	[3.90; 12.03]	1.0%
Robledo−Aceves, 2018	3	134	2.24	[0.46; 6.40]	0.9%
Salvador García, 2012	14	235	5.96	[3.29; 9.79]	1.0%
Wollmeister, 2018	17	314	5.41	[3.19; 8.53]	1.0%
Zhou, 2015	5	60	8.33	[2.76; 18.39]	0.8%
**Random effect meta−analysis**		**6995**	**7.07**	**[4.64; 9.94]**	**19.4%**
Heterogeneity: I^2 = 93.9% [91.8%; 95.4%], τ^2 = 0.0120, p < 0.0001					
**HRSV + HBoV (7 studies)**					
Bamberger, 2012	18	309	5.83	[3.49; 9.05]	1.0%
Cangiano, 2016	13	351	3.70	[1.99; 6.25]	1.0%
Midulla, 2010	15	182	8.24	[4.69; 13.23]	1.0%
Papoff, 2011	15	310	4.84	[2.73; 7.86]	1.0%
Petrarca, 2018	18	486	3.70	[2.21; 5.79]	1.0%
Salvador García, 2012	22	235	9.36	[5.96; 13.83]	1.0%
Wollmeister, 2018	1	314	0.32	[0.01; 1.76]	1.0%
**Random effect meta−analysis**		**2187**	**4.54**	**[2.43; 7.25]**	**7.1%**
Heterogeneity: I^2 = 86.2% [73.6%; 92.8%], τ^2 = 0.0050, p < 0.0001					
**HRSV + HCoV (3 study)**					
Richard, 2008	4	180	2.22	[0.61; 5.59]	1.0%
Tsou, 2020	12	270	4.44	[2.32; 7.63]	1.0%
Wollmeister, 2018	2	314	0.64	[0.08; 2.28]	1.0%
**Random effect meta−analysis**		**764**	**2.15**	**[0.38; 5.11]**	**3.0%**
Heterogeneity: I^2 = 79% [33.0%; 93.4%], τ^2 = 0.0038, p = 0.0085					
**HRSV + HAdV (9 studies)**					
Azkur, 2014	1	55	1.82	[0.05; 9.72]	0.8%
Bamberger, 2012	4	309	1.29	[0.35; 3.28]	1.0%
Bekhof, 2013	0	48	0.00	[0.00; 7.40]	0.7%
Brand, 2012	9	142	6.34	[2.94; 11.69]	0.9%
Papadopoulos, 2002	2	118	1.69	[0.21; 5.99]	0.9%
Richard, 2008	1	180	0.56	[0.01; 3.06]	1.0%
Salvador García, 2012	12	235	5.11	[2.67; 8.75]	1.0%
Tsou, 2020	6	270	2.22	[0.82; 4.77]	1.0%
Wollmeister, 2018	4	314	1.27	[0.35; 3.23]	1.0%
**Random effect meta−analysis**		**1671**	**1.96**	**[0.93; 3.30]**	**8.4%**
Heterogeneity: I^2 = 58.8% [13.9%; 80.3%], τ^2 = 0.0020, p = 0.0128					
**HCoV + HPIV (2 study)**					
Bekhof, 2013	1	48	2.08	[0.05; 11.07]	0.7%
Wollmeister, 2018	1	314	0.32	[0.01; 1.76]	1.0%
**Random effect meta−analysis**		**362**	**0.50**	**[0.00; 3.22]**	**1.8%**
Heterogeneity: I^2 = 46.4%, τ^2 = 0.0026, p = 0.1721					
**HBoV + HAdV (1 study)**					
Azkur, 2014	1	55	1.82	[0.05; 9.72]	0.8%
**Random effect meta−analysis**		**55**	**1.82**	**[0.00; 7.64]**	**0.8%**
Heterogeneity: not applicable					
**HBoV + Influenza (1 study)**					
Azkur, 2014	1	55	1.82	[0.05; 9.72]	0.8%
**Random effect meta−analysis**		**55**	**1.82**	**[0.00; 7.64]**	**0.8%**
Heterogeneity: not applicable					
**HRSV + HMPV (10 studies)**					
Bamberger, 2012	6	309	1.94	[0.72; 4.18]	1.0%
Bekhof, 2013	2	48	4.17	[0.51; 14.25]	0.7%
Cangiano, 2016	2	351	0.57	[0.07; 2.04]	1.0%
Petrarca, 2018	3	486	0.62	[0.13; 1.79]	1.0%
Richard, 2008	5	180	2.78	[0.91; 6.36]	1.0%
Robledo−Aceves, 2018	2	134	1.49	[0.18; 5.29]	0.9%
Teeratakulpisarn, 2007	1	170	0.59	[0.01; 3.23]	1.0%
Tsou, 2020	1	270	0.37	[0.01; 2.05]	1.0%
Wollmeister, 2018	2	314	0.64	[0.08; 2.28]	1.0%
Xepapadaki, 2004	4	56	7.14	[1.98; 17.29]	0.8%
**Random effect meta−analysis**		**2318**	**1.09**	**[0.46; 1.92]**	**9.5%**
Heterogeneity: I^2 = 52.4% [2.3%; 76.8%], τ^2 = 0.0012, p = 0.0261					
**HRSV + HPIV (10 studies)**					
Bekhof, 2013	1	48	2.08	[0.05; 11.07]	0.7%
Cangiano, 2016	1	351	0.28	[0.01; 1.58]	1.0%
Papadopoulos, 2002	1	118	0.85	[0.02; 4.63]	0.9%
Papoff, 2011	1	310	0.32	[0.01; 1.78]	1.0%
Petrarca, 2018	1	486	0.21	[0.01; 1.14]	1.0%
Richard, 2008	4	180	2.22	[0.61; 5.59]	1.0%
Robledo−Aceves, 2018	5	134	3.73	[1.22; 8.49]	0.9%
Tsou, 2020	4	270	1.48	[0.41; 3.75]	1.0%
Wollmeister, 2018	2	314	0.64	[0.08; 2.28]	1.0%
Zhou, 2015	2	60	3.33	[0.41; 11.53]	0.8%
**Random effect meta−analysis**		**2271**	**0.88**	**[0.30; 1.69]**	**9.5%**
Heterogeneity: I^2 = 54.7% [7.6%; 77.7%], τ^2 = 0.0014, p = 0.0189					
**RV + HMPV (4 studies)**					
Cangiano, 2016	4	351	1.14	[0.31; 2.89]	1.0%
Papoff, 2011	1	310	0.32	[0.01; 1.78]	1.0%
Petrarca, 2018	5	486	1.03	[0.33; 2.38]	1.0%
Richard, 2008	3	180	1.67	[0.35; 4.79]	1.0%
**Random effect meta−analysis**		**1327**	**0.91**	**[0.43; 1.54]**	**4.0%**
Heterogeneity: I^2 = 0% [0.0%; 82.2%], τ^2 = 0, p = 0.4601					
**HRSV + Influenza (9 studies)**					
Bamberger, 2012	4	309	1.29	[0.35; 3.28]	1.0%
Cangiano, 2016	2	351	0.57	[0.07; 2.04]	1.0%
Midulla, 2010	1	182	0.55	[0.01; 3.02]	1.0%
Papoff, 2011	1	310	0.32	[0.01; 1.78]	1.0%
Petrarca, 2018	2	486	0.41	[0.05; 1.48]	1.0%
Richard, 2008	4	180	2.22	[0.61; 5.59]	1.0%
Robledo−Aceves, 2018	4	134	2.99	[0.82; 7.47]	0.9%
Wollmeister, 2018	1	314	0.32	[0.01; 1.76]	1.0%
Zhou, 2015	1	60	1.67	[0.04; 8.94]	0.8%
**Random effect meta−analysis**		**2326**	**0.73**	**[0.31; 1.30]**	**8.8%**
Heterogeneity: I^2 = 32.3% [0.0%; 68.8%], τ^2 = 0.0005, p = 0.1593					
**HCoV + HAdV (2 studies)**					
Papadopoulos, 2002	1	118	0.85	[0.02; 4.63]	0.9%
Tsou, 2020	2	270	0.74	[0.09; 2.65]	1.0%
**Random effect meta−analysis**		**388**	**0.73**	**[0.04; 1.95]**	**1.9%**
Heterogeneity: I^2 = 0%, τ^2 = 0, p = 0.7827					
**RV + HAdV + HCoV (1 study)**					
Papadopoulos, 2002	1	118	0.85	[0.02; 4.63]	0.9%
**Random effect meta−analysis**		**118**	**0.85**	**[0.00; 3.60]**	**0.9%**
Heterogeneity: not applicable					
**RV + HCoV (4 studies)**					
Azkur, 2014	1	55	1.82	[0.05; 9.72]	0.8%
Papadopoulos, 2002	1	118	0.85	[0.02; 4.63]	0.9%
Richard, 2008	1	180	0.56	[0.01; 3.06]	1.0%
Robledo−Aceves, 2018	1	134	0.75	[0.02; 4.09]	0.9%
**Random effect meta−analysis**		**487**	**0.71**	**[0.06; 1.83]**	**3.6%**
Heterogeneity: I^2 = 0% [0.0%; 48.8%], τ^2 = 0, p = 0.8260					
**HRSV + EV (1 study)**					
Richard, 2008	1	180	0.56	[0.01; 3.06]	1.0%
**Random effect meta−analysis**		**180**	**0.56**	**[0.00; 2.37]**	**1.0%**
Heterogeneity: not applicable					
**RV + EV (1 study)**					
Richard, 2008	1	180	0.56	[0.01; 3.06]	1.0%
**Random effect meta−analysis**		**180**	**0.56**	**[0.00; 2.37]**	**1.0%**
Heterogeneity: not applicable					
**Influenza + HPIV (1 study)**					
Richard, 2008	1	180	0.56	[0.01; 3.06]	1.0%
**Random effect meta−analysis**		**180**	**0.56**	**[0.00; 2.37]**	**1.0%**
Heterogeneity: not applicable					
**RV + HPIV (4 studies)**					
Cangiano, 2016	1	351	0.28	[0.01; 1.58]	1.0%
Petrarca, 2018	1	486	0.21	[0.01; 1.14]	1.0%
Richard, 2008	3	180	1.67	[0.35; 4.79]	1.0%
Wollmeister, 2018	1	314	0.32	[0.01; 1.76]	1.0%
**Random effect meta−analysis**		**1331**	**0.39**	**[0.05; 0.94]**	**4.1%**
Heterogeneity: I^2 = 23.8% [0.0%; 88.3%], τ^2 = 0.0002, p = 0.2683					
**RV + HBoV (2 studies)**					
Azkur, 2014	1	55	1.82	[0.05; 9.72]	0.8%
Petrarca, 2018	1	486	0.21	[0.01; 1.14]	1.0%
**Random effect meta−analysis**		**541**	**0.38**	**[0.00; 3.06]**	**1.8%**
Heterogeneity: I^2 = 56.5% [0.0%; 89.5%], τ^2 = 0.0033, p = 0.1297					
**RV + HAdV (3 studies)**					
Azkur, 2014	1	55	1.82	[0.05; 9.72]	0.8%
Petrarca, 2018	1	486	0.21	[0.01; 1.14]	1.0%
Richard, 2008	1	180	0.56	[0.01; 3.06]	1.0%
**Random effect meta−analysis**		**721**	**0.25**	**[0.00; 1.14]**	**2.8%**
Heterogeneity: I^2 = 23.4% [0.0%; 92.0%], τ^2 = 0.0004, p = 0.2709					
**HBoV + HMPV (3 studies)**					
Cangiano, 2016	1	351	0.28	[0.01; 1.58]	1.0%
Midulla, 2010	1	182	0.55	[0.01; 3.02]	1.0%
Papoff, 2011	1	310	0.32	[0.01; 1.78]	1.0%
**Random effect meta−analysis**		**843**	**0.33**	**[0.01; 0.92]**	**3.0%**
Heterogeneity: I^2 = 0% [0.0%; 34.0%], τ^2 = 0, p = 0.8543					
**HBoV + HPIV (1 study)**					
Cangiano, 2016	1	351	0.28	[0.01; 1.58]	1.0%
**Random effect meta−analysis**		**351**	**0.28**	**[0.00; 1.22]**	**1.0%**
Heterogeneity: not applicable					
**HRSV + RV + HMPV (3 studies)**					
Cangiano, 2016	1	351	0.28	[0.01; 1.58]	1.0%
Petrarca, 2018	1	486	0.21	[0.01; 1.14]	1.0%
Wollmeister, 2018	1	314	0.32	[0.01; 1.76]	1.0%
**Random effect meta−analysis**		**1151**	**0.25**	**[0.01; 0.68]**	**3.1%**
Heterogeneity: I^2 = 0% [0.0%; 0.0%], τ^2 = 0, p = 0.9254					
**HRSV + RV + HAdV (1 study)**					
Wollmeister, 2018	2	314	0.64	[0.08; 2.28]	1.0%
**Random effect meta−analysis**		**314**	**0.64**	**[0.01; 1.91]**	**1.0%**
Heterogeneity: not applicable					
**HRSV + RV + HPIV (1 study)**					
Wollmeister, 2018	1	314	0.32	[0.01; 1.76]	1.0%
**Random effect meta−analysis**		**314**	**0.32**	**[0.00; 1.36]**	**1.0%**
Heterogeneity: not applicable					
**Overall random effect meta−analysis**		**27430**	**2.00**	**[1.40; 2.69]**	**100.0%**
Residual heterogeneity: I^2 = 82.4% [78.6%; 85.5%], p < 0.0001					

S3 Fig. Funnel plot for publication for HRSV in people with bronchiolitis

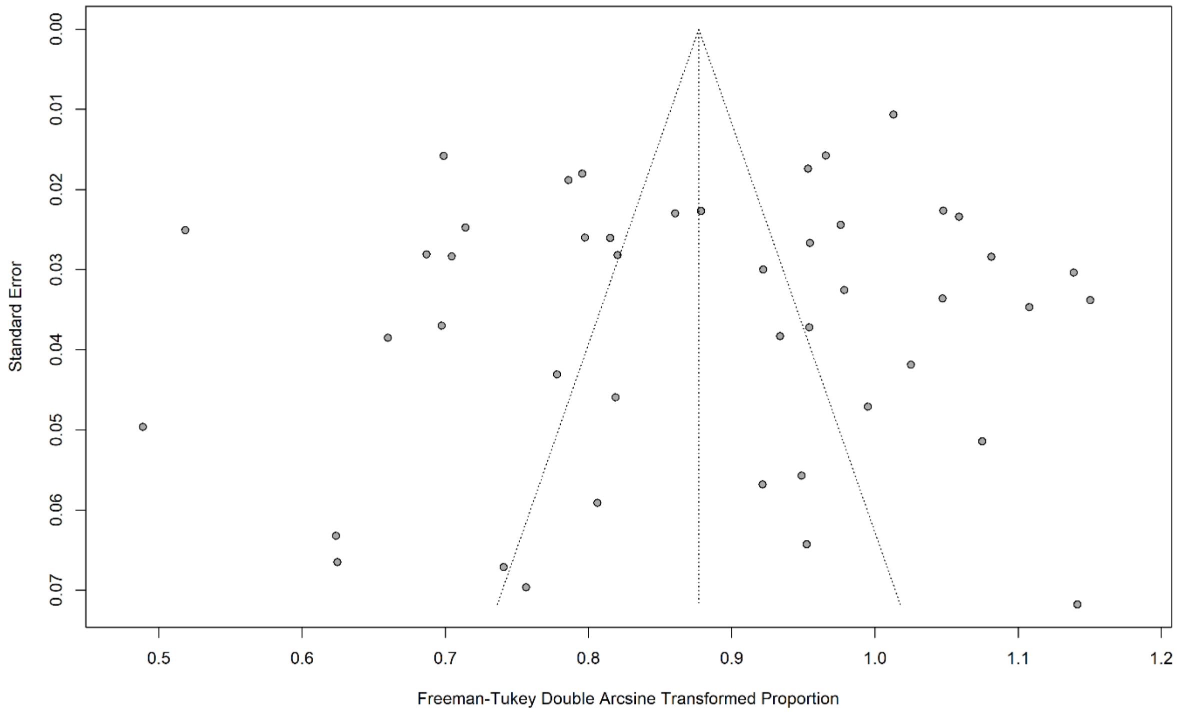

S4 Fig. Funnel plot for publication for RV in people with bronchiolitis

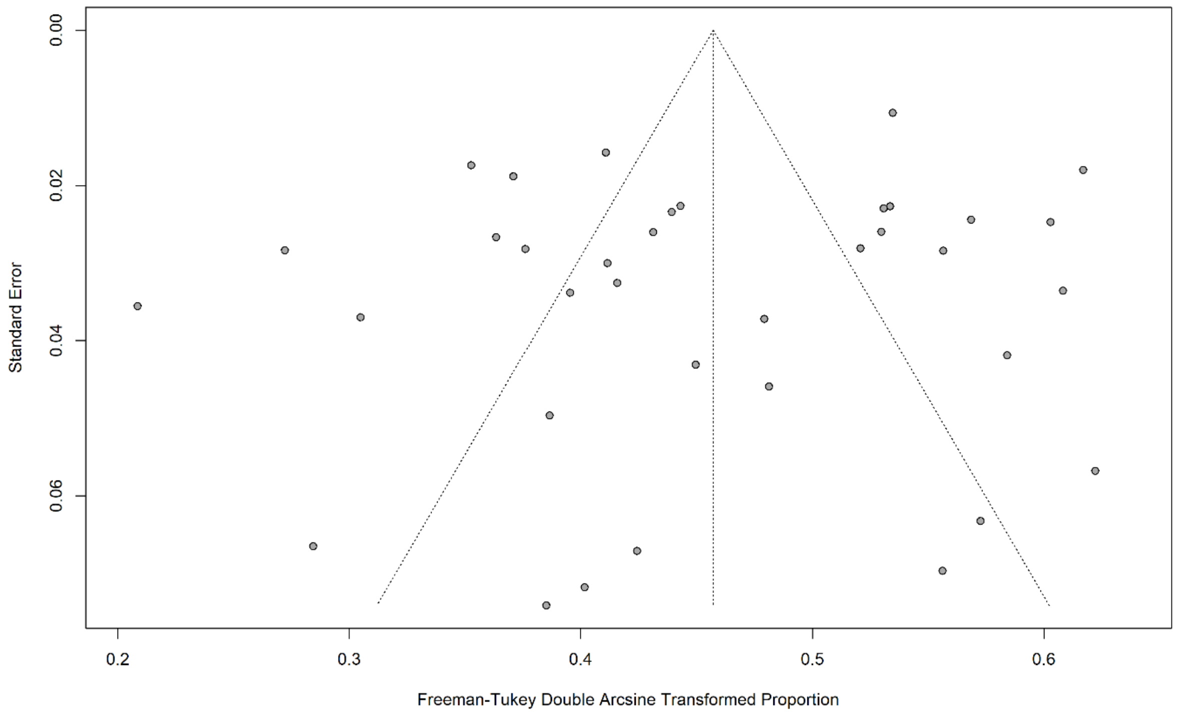

S5 Fig. Funnel plot for publication for HBoV in people with bronchiolitis

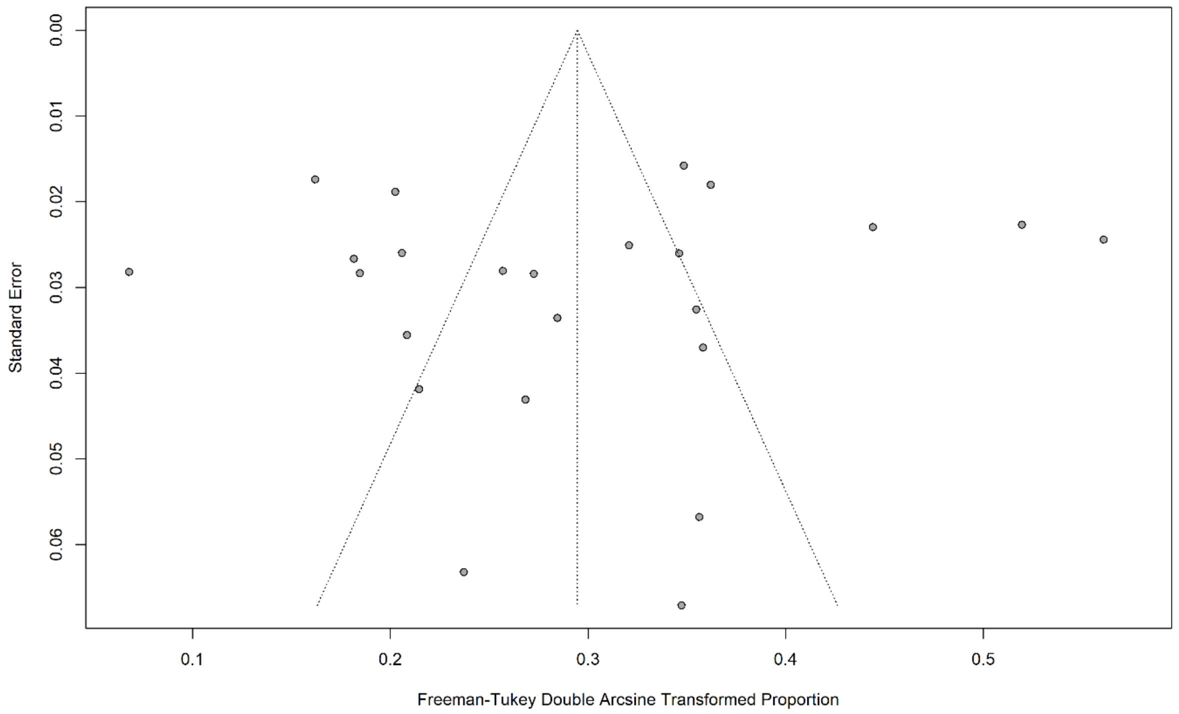

S6 Fig. Funnel plot for publication for HAdV in people with bronchiolitis

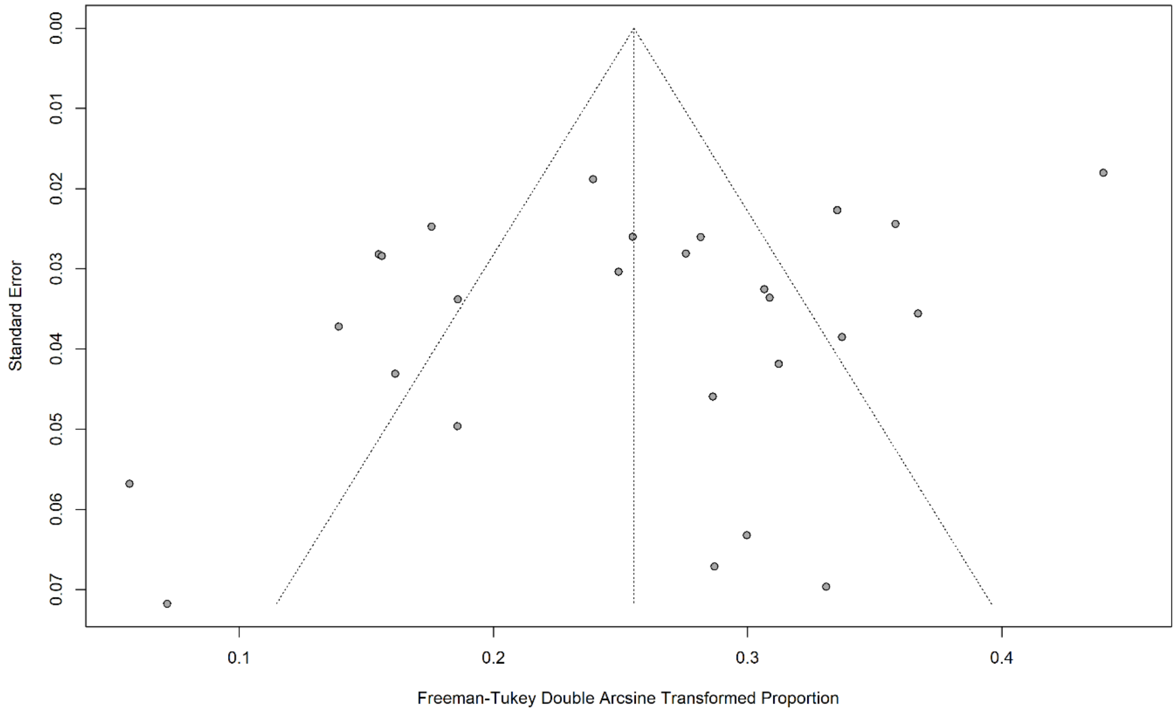

S7 Fig. Funnel plot for publication for HMPV in people with bronchiolitis

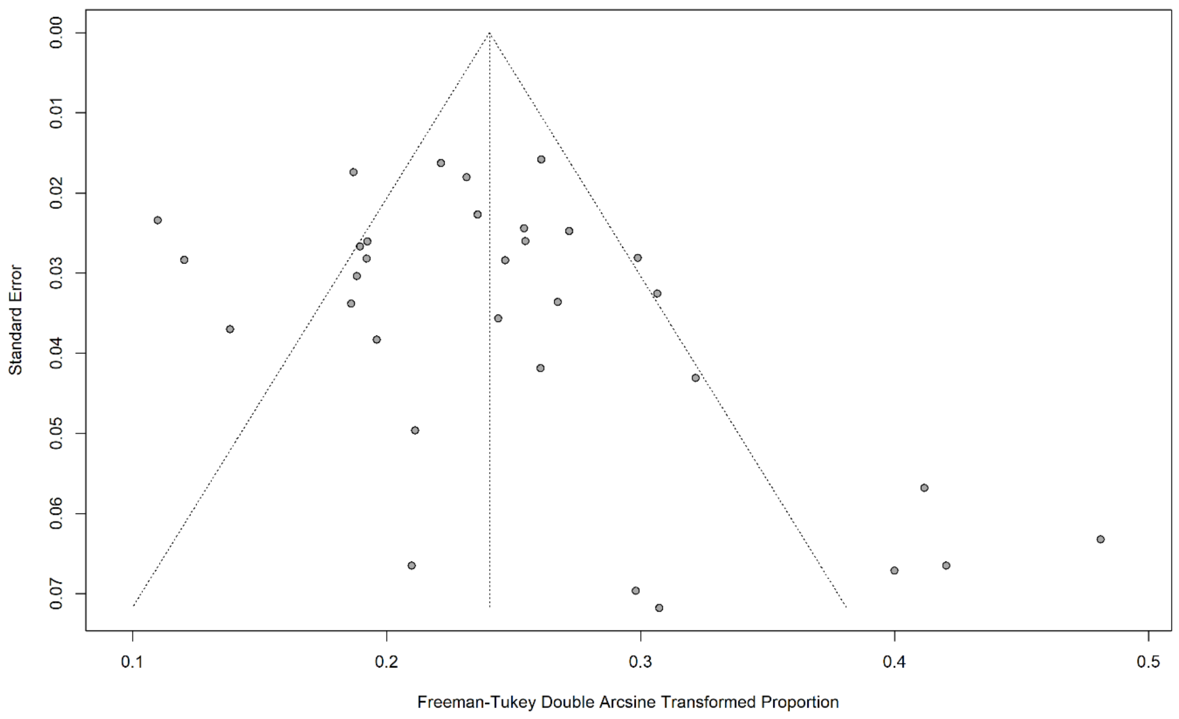

S8 Fig. Funnel plot for publication for HPIV in people with bronchiolitis

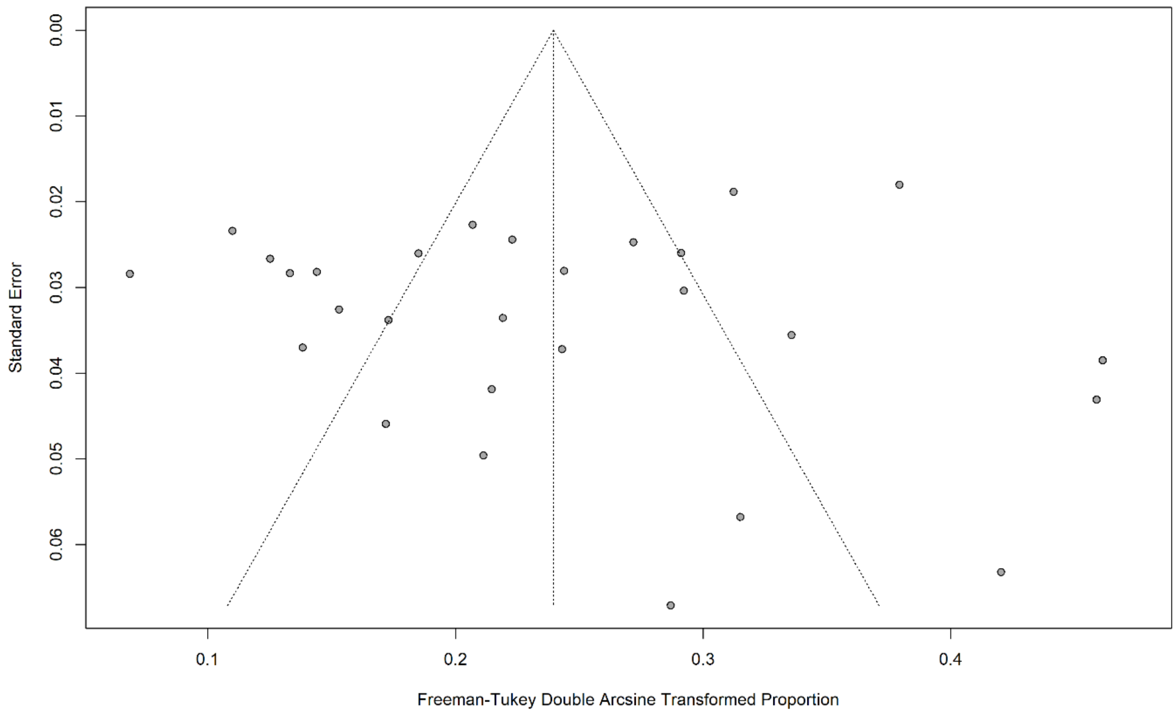

S9 Fig. Funnel plot for publication for Influenza in people with bronchiolitis

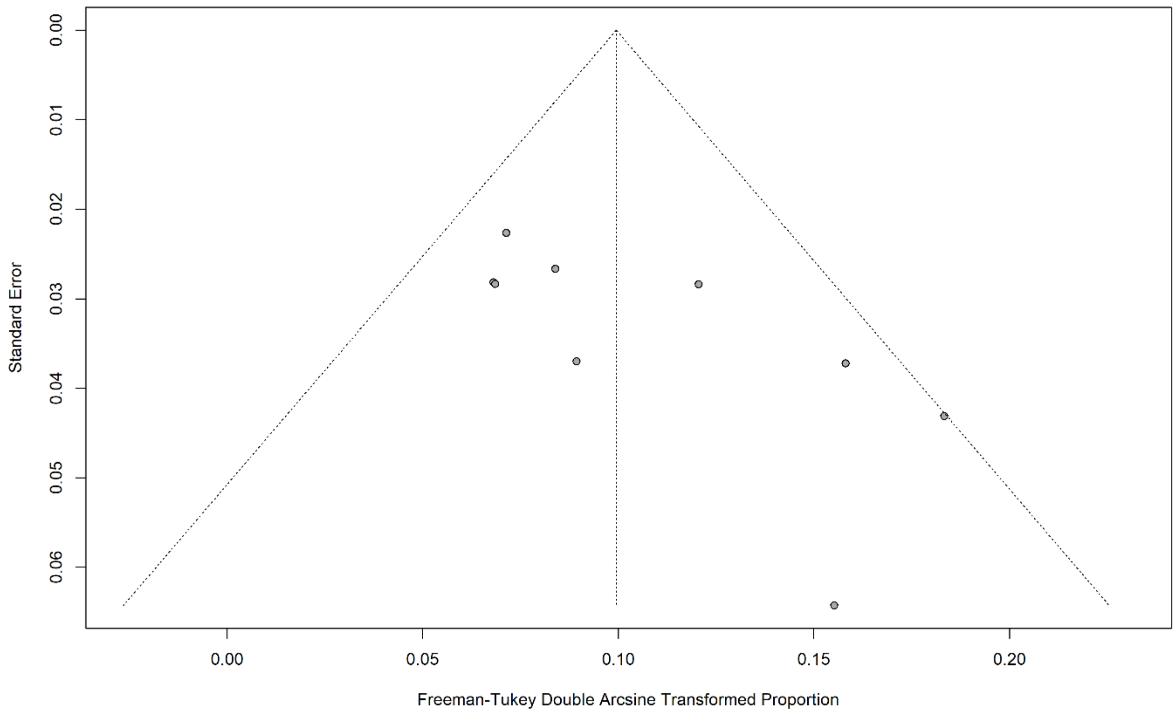

S10 Fig. Funnel plot for publication for EV in people with bronchiolitis

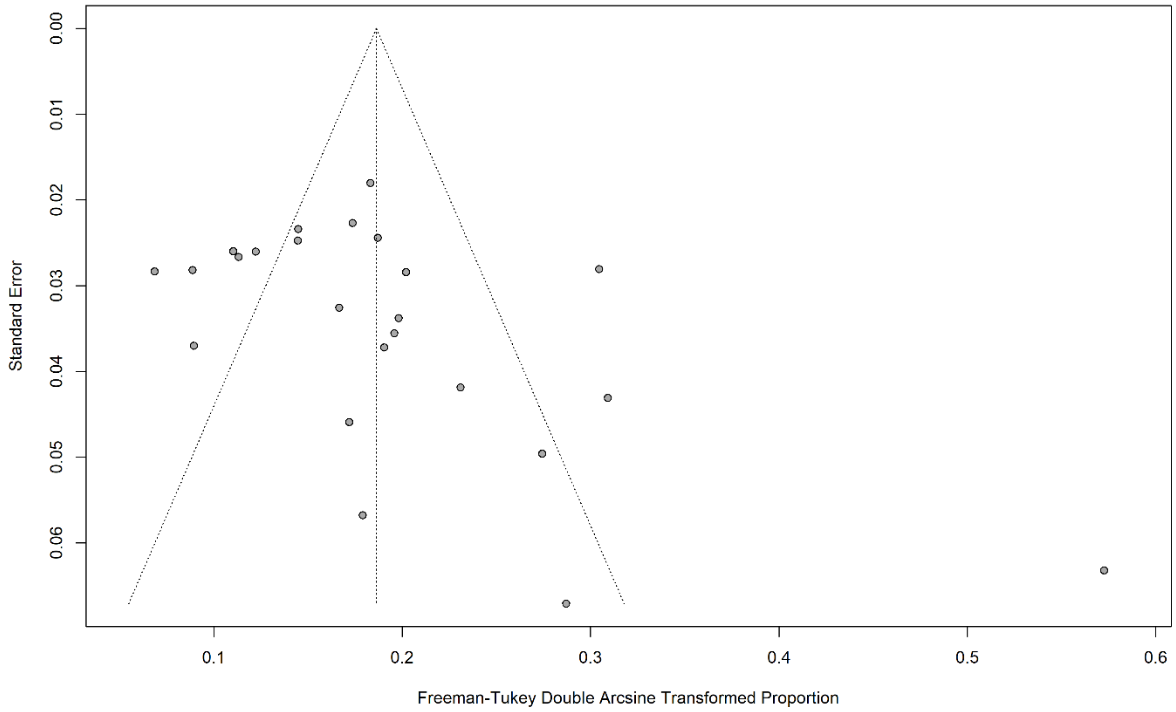

S11 Fig. Funnel plot for publication for HCoV in people with bronchiolitis

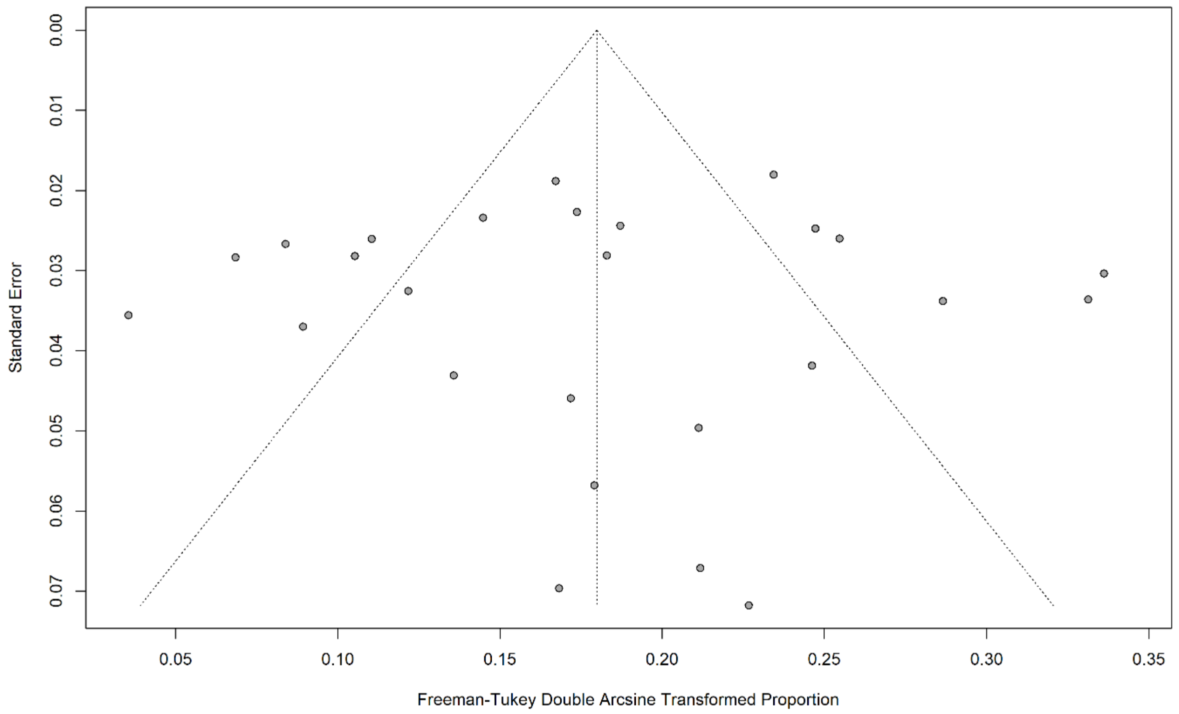

Supplement: S1 File — (ZIP) [file pone.0242302.s002.zip › S1 Table.pdf]
