# Supplementary material for: Systematic review and meta-analysis of the prevalence of common respiratory viruses in children < 2 years with bronchiolitis in the pre-COVID-19 pandemic era
Source: PLoS One. 2020 Nov 12;15(11):e0242302. doi: 10.1371/journal.pone.0242302 (PMC7660462; doi:10.1371/journal.pone.0242302)
Supplement: S1 File — (ZIP) [file pone.0242302.s002.zip › S2 Table.pdf]

S2 Table. Search strategy in Medline (Pubmed)

**Search**	**Virus**
#1	“HRSV” OR “RSV” OR “human respiratory syncytial virus”OR “respiratory syncytial virus” OR “HRSV-A” OR “HRSV-B” OR “HMPV” OR “MPV” OR “human metapneumovirus”OR “metapneumovirus” OR “HMPV-A” OR “HMPV-B” OR “HAdV” OR “AdV” OR “Adenovirus” OR “Adenovirus Infections, Human” OR “Human adenovirus” OR “HADV-A” OR “HADV-B” OR “HADV-C” OR “HADV-D” OR “HADV-E” OR “HADV-F” OR “HADV-G” OR “HBoV” OR “BoV” OR “Bocavirus” OR “Bocavirus Infections, Human” OR “Human Bocavirus” OR “HCoV” OR “CoV” OR “Coronavirus” OR “Coronavirus Infections, Human” OR “Human Coronavirus” OR “229E” OR “OC43” OR “NL63” OR “HKU1” OR “HCoV-229E” OR “HCoV-OC43” OR “HCoV-NL63” OR “HCoV-HKU1” OR “HPIV” OR “PIV” OR “Parainfluenzavirus” OR “Parainfluenzavirus Infections, Human” OR “Human Parainfluenzavirus” OR “PIV-1” OR “PIV-2” OR “PIV-3” OR “PIV-4” OR “HPIV-1” OR “HPIV-2” OR “HPIV-3” OR “HPIV-4” OR “HEV” OR “EV” OR “Enterovirus” OR “Enterovirus Infections, Human” OR “Human Enterovirus” OR “HRV” OR “RV” OR “Rhinovirus” OR “Rhinoviruses” OR “Rhinovirus Infections, Human” OR “Human Rhinovirus” OR “RV-A” OR “RV-B” OR “RV-C” OR “Influenza” OR “Inf” OR “Influenza virus” OR “Influenza, Human” OR “Influenza-A virus” OR “Influenza-B virus” OR “Influenza-C virus”
#2	bronchiolitis
#3	#1 AND #2
