# Supplementary material for: Systematic review and meta-analysis of the prevalence of common respiratory viruses in children < 2 years with bronchiolitis in the pre-COVID-19 pandemic era
Source: PLoS One. 2020 Nov 12;15(11):e0242302. doi: 10.1371/journal.pone.0242302 (PMC7660462; doi:10.1371/journal.pone.0242302)
Supplement: S1 File — (ZIP) [file pone.0242302.s002.zip › S3 Table.pdf]

S3 Table. Items for risk of bias assessment

**Hoy et al. tool for cross sectional studies**	**Yes (1)/No (0)**
**External validity**	
1. Was the study's target population a close representation of the national population in relation to relevant variables?	**1**
2. Was the sampling frame a true or close representation of the bronchiolitis population?	**1**
3. Was some form of random selection used to select the sample, OR was a census undertaken?	**1**
4. Was the likelihood of non response bias minimal?	**1**
**Internal validity**	
5. Were data collected directly from the subjects (as opposed to a proxy)?	**1**
6. Was an acceptable bronchiolitis case definition used in the study?	**1**
7. Was the study viral detection assay shown to have validity and reliability?	**1**
8. Was the same mode type of sample collected for all subjects?	**1**
9. Was the length of the shortest prevalence period for the parameter of interest appropriate?	**1**
10. Were the numerator(s) and denominator(s) for the parameter of interest appropriate?	**1**
Total score	**10**
**Interpretation of the risk of bias tool**<br><br>• 7-10: Low risk of bias<br>• 4-6: Moderate risk of bias<br>• 0-3: High risk of bias	
