# Supplementary material for: Systematic review and meta-analysis of the prevalence of common respiratory viruses in children < 2 years with bronchiolitis in the pre-COVID-19 pandemic era
Source: PLoS One. 2020 Nov 12;15(11):e0242302. doi: 10.1371/journal.pone.0242302 (PMC7660462; doi:10.1371/journal.pone.0242302)
Supplement: S1 File — (ZIP) [file pone.0242302.s002.zip › S4 Table.pdf]

S4 Table. Main reasons of exclusion of eligible studies

N°	Name, Year	Title	Reason of exclusion
1	Akhras, 2010	Human metapneumovirus and respiratory syncytial virus: subtle differences but comparable severity	No molecular assays used
2	Alonso, 2007	Bronchiolitis due to respiratory syncytial virus in hospitalized children: a study of seasonal rhythm.	Only positive samples included
3	Al-Shawwa, 2007	Clinical and therapeutic variables influencing hospitalisation for bronchiolitis in a community-based paediatric group practice	No molecular assays used
4	Al-Shehri, 2005	Bronchiolitis in Abha, Southwest Saudi Arabia: viral etiology and predictors for hospital admission.	No molecular assays used
5	Alvarez, 2013	Epidemiological and genetic characteristics associated with the severity of acute viral bronchiolitis by respiratory syncytial virus.	Systematic review
6	Amér, 2009	[Not only RSV can cause bronchiolitis in small children	Full text not found
7	Bakalovic, 2015	Epidemiological Features of Bronchiolitis in the Pediatric Clinic of Clinical center of Sarajevo University.	Data on detection assay not reported
8	Barr, 2018	Change in viral bronchiolitis management in hospitals in the UK after the publication of NICE guideline.	No data on viral etiology searched
9	Bashir, 2017	Respiratory syncytial virus and influenza are the key viral pathogens in children <2 years hospitalized with bronchiolitis and pneumonia in Islamabad Pakistan.	Not possible to extract data on viral etiology searched
10	Bauert, 2019	Rhinovirus Species in Children With Severe Bronchiolitis: Multicenter Cohort Studies in the United States and Finland	Duplicates
11	Bennett, 2018	Population-based trends and underlying risk factors for infant respiratory syncytial virus and bronchiolitis hospitalizations.	Inappropriate study population
12	Berezin, 2006	Rhinovirus and bronchiolitis.	Comments
13	Bilavsky, 2010	Respiratory syncytial virus-positive bronchiolitis in hospitalized infants is associated with thrombocytosis.	No molecular assays used
14	Bochkov, 2020	A 14-year Prospective Study of Human Coronavirus Infections in Hospitalized Children: Comparison With Other Respiratory Viruses.	Inappropriate study population
15	Bouscambert-Duchamp, 2005	Detection of human metapneumovirus RNA sequences in nasopharyngeal aspirates of young French children with acute bronchiolitis by real-time reverse transcriptase PCR and phylogenetic analysis.	Duplicate of Janahi, 2017
16	Boyce, 2004	Incidence of bronchiolitis-associated hospitalization among children in Olmsted County, Minnesota.	Comments
17	Boyce, 2016	More on Viral Bronchiolitis in Children.	Comments

18	Bradley, 2005	Severity of respiratory syncytial virus bronchiolitis is affected by cigarette smoke exposure and atopy.	Only positive samples included
19	Brini, 2018	Temporal and climate characteristics of respiratory syncytial virus bronchiolitis in neonates and children in Sousse, Tunisia, during a 13-year surveillance	No molecular assays used
20	Brini, 2020	Temporal and climate characteristics of respiratory syncytial virus bronchiolitis in neonates and children in Sousse, Tunisia, during a 13-year surveillance.	Inappropriate detection assay
21	Calvo, 2015	Respiratory Syncytial Virus Coinfections With Rhinovirus and Human Bocavirus in Hospitalized Children	> 2 years
22	Cantani, 1999	Bronchiolitis in infants.	No data on viral etiology searched
23	Causse, 2007	Preliminary evaluation of a multiplex reverse transcription-PCR assay combined with a new DNA chip hybridization assay for detecting respiratory syncytial virus	> 2 years
24	Cavallin, 2013	Infection with multiple viruses is not associated with increased disease severity in children with bronchiolitis.	Comments
25	Centers for Disease Control and Prevention (CDC), 2003	Bronchiolitis-associated outpatient visits and hospitalizations among American Indian and Alaska Native children--United States, 1990-2000.	Report
26	Chan, 2002	Risk factors for hypoxemia and respiratory failure in respiratory syncytial virus bronchiolitis.	Only positive samples included
27	Chee, 2010	Emergency Department Septic Screening in Respiratory Syncytial Virus (RSV) and Non-RSV Bronchiolitis.	No molecular assays used
28	Chen, 2004	[Clinical characteristics of bronchiolitis caused by human metapneumovirus in infants]	Full text not found
29	Chen, 2014	Viral etiology of bronchiolitis among pediatric inpatients in northern Taiwan with emphasis on newly identified respiratory viruses.	Combination of multiple asays for detection
30	Ciarlitto, 2019	Respiratory Syncityal Virus A and B: three bronchiolitis seasons in a third level hospital in Italy.	Inappropriate study population
31	Connors, 2016	Viral Bronchiolitis in Children.	Comments
32	Coskun, 2017	Risk factors for intensive care need in children with bronchiolitis: A case-control study.	Data on detection assay not reported
33	Côté, 2003	Comparative evaluation of real-time PCR assays for detection of the human metapneumovirus	No data on viral etiology searched
34	CRONE, 1964	SEROLOGICAL EVIDENCE OF INFECTION BY RESPIRATORY SYNCYTIAL VIRUS IN OUTBREAK OF ACUTE BRONCHIOLITIS	No molecular assays used
35	Cubie, 1992	Detection of respiratory syncytial virus in acute bronchiolitis in infants	Not possible to extract data on viral etiology searched

36	Dagan, 1993	Hospitalization of Jewish and Bedouin infants in southern Israel for bronchiolitis caused by respiratory syncytial virus	No molecular assays used
37	Davies, 2017	A systematic review of the psychometric properties of bronchiolitis assessment tools.	Systematic review
38	DeVincenzo, 2016	Viral Bronchiolitis in Children.	Comments
39	Dotan, 2013	Hospitalization for respiratory syncytial virus bronchiolitis and disease severity in twins.	Only positive samples included
40	Duttweiler, 2004	Pulmonary and systemic bacterial co-infections in severe RSV bronchiolitis.	Only positive samples included
41	Eidelman, 2009	The burden of respiratory syncytial virus bronchiolitis on a pediatric inpatient service.	No molecular assays used
42	Erculj, 2018	PM10 exposure is associated with increased hospitalizations for respiratory syncytial virus bronchiolitis among infants in Lombardy, Italy.	Only positive samples included
43	Erez, 2012	[Prevalence of H1N1 A influenza virus infection among hospitalized patients with bronchiolitis twelve months old and younger]	Full text not found
44	Eugene-Ruellan, 1998	Detection of respiratory syncytial virus A and B and parainfluenzavirus 3 sequences in respiratory tracts of infants by a single PCR with primers targeted to the L-polymerase gene and differential hybridization	Age range not reported
45	FANDRE, 1964	[EPIDEMIC OF ACUTE BRONCHIOLITIS IN INFANTS. ISOLATION OF A RESPIRATORY SYNEYTIAL VIRUS]	Full text not found
46	Fauroux, 2017	The Burden and Long-term Respiratory Morbidity Associated with Respiratory Syncytial Virus Infection in Early Childhood.	Systematic review
47	Fedele, 2018	Analysis of the immune response in infants hospitalized with viral bronchiolitis shows different Th1/Th2 profiles associated with respiratory syncytial virus and human rhinovirus.	Inappropriate study population
48	Ferronato, 2012	Etiological diagnosis reduces the use of antibiotics in infants with bronchiolitis.	No molecular assays used
49	Fjaerli, 2004	Hospitalisations for respiratory syncytial virus bronchiolitis in Akershus, Norway, 1993-2000: a population-based retrospective study.	No molecular assays used
50	Flaherman, 2010	Respiratory syncytial virus testing during bronchiolitis episodes of care in an integrated health care delivery system: a retrospective cohort study.	No molecular assays used
51	Flamant, 2005	Severe respiratory syncytial virus bronchiolitis in children: from short mechanical ventilation to extracorporeal membrane oxygenation.	No molecular assays used
52	Flores, 2004	Bronchiolitis caused by respiratory syncytial virus in an area of portugal: epidemiology, clinical features, and risk factors.	> 2 years
53	Fodha, 2007	Respiratory syncytial virus infections in hospitalized infants: association between viral load, virus subgroup, and disease severity	Only positive samples included

54	Foley, 2018	Respiratory health inequality starts early: The impact of social determinants on the aetiology and severity of bronchiolitis in infancy.	No molecular assays used
55	Foo, 1991	Severe bronchiolitis in children	Full text not found
56	FORBES, 1961	Epidemic bronchiolitis caused by a respiratory syncytial virus: clinical aspects	Conference abstract
57	Fretzayas, 2017	Etiology and clinical features of viral bronchiolitis in infancy.	Review
58	Freymouth, 2003	Presence of the new human metapneumovirus in French children with bronchiolitis.	Age range not reported
59	Fujiogi, 2020	Association of rhinovirus species with nasopharyngeal metabolome in bronchiolitis infants: A multicenter study.	Duplicates
60	Ganu, 2012	Increase in use of non-invasive ventilation for infants with severe bronchiolitis is associated with decline in intubation rates over a decade	Data on detection assay not reported
61	Garcia, 2010	Risk factors in children hospitalized with RSV bronchiolitis versus non-RSV bronchiolitis.	No molecular assays used
62	Garcia-Marcos, 2014	Pediatricians' attitudes and costs of bronchiolitis in the emergency department: a prospective multicentre study.	No data on viral etiology searched
63	Ghazaly, 2018	Characteristics of children admitted to intensive care with acute bronchiolitis.	Combination of multiple asays for detection
64	Ghazaly, 2018	Characteristics of children admitted to intensive care with acute bronchiolitis.	Inappropriate detection assay
65	Giordano, 2018	Respiratory syncytial virus bronchiolitis and hypertransaminasemia.	Case report
66	Gold, 2006	[Respiratory syncytial virus bronchiolitis: severe respiratory forms in hospitalized infants]	Review
67	González Martínez, 2013	[Clinical impact of introducing ventilation with high flow oxygen in the treatment of bronchiolitis in a paediatric ward]	No molecular assays used
68	Goto-Sugai, 2010	Genotyping and phylogenetic analysis of the major genes in respiratory syncytial virus isolated from infants with bronchiolitis.	Only positive samples included
69	Greensill, 2003	Human metapneumovirus in severe respiratory syncytial virus bronchiolitis.	Age range not reported
70	Grimaldi, 2002	[Prospective regional study of an epidemic of respiratory syncytial virus	Only positive samples included
71	Grimwood, 2008	Risk factors for respiratory syncytial virus bronchiolitis hospital admission in New Zealand.	Combination of multiple asays for detection
72	Grubbauer, 1989	[Respiratory insufficiency in acute bronchiolitis in infancy]	Full text not found
73	Haque, 2012	Bronchiolitis outbreak caused by respiratory syncytial virus in southwest Bangladesh, 2010.	Outbreak
74	Hasegawa, 2014	Multicenter study of viral etiology and relapse in hospitalized children with bronchiolitis.	Duplicate of Dumas, 2016
75	Hasegawa, 2014	Infectious pathogens and bronchiolitis outcomes.	Review
76	Hasegawa, 2015	Risk factors for requiring intensive care among children admitted to ward with bronchiolitis.	Duplicate of Dumas, 2016

77	Hasegawa, 2015	Respiratory syncytial virus genomic load and disease severity among children hospitalized with bronchiolitis: multicenter cohort studies in the United States and Finland.	Duplicate of Dumas, 2016
78	Hasegawa, 2018	Rhinovirus Species in Children with Severe Bronchiolitis: Multicenter Cohort Studies in the US and Finland.	Duplicate of Dumas, 2016
79	Hasegawa, 2019	Respiratory Virus Epidemiology Among US Infants With Severe Bronchiolitis: Analysis of 2 Multicenter, Multiyear Cohort Studies.	Duplicates
80	Hasegawa, 2019	Association of Rhinovirus C Bronchiolitis and Immunoglobulin E Sensitization During Infancy With Development of Recurrent Wheeze.	Duplicates
81	Heinonen, 2018	Transient Tachypnea of the Newborn is Associated with an Increased Risk of Hospitalization Due to RSV Bronchiolitis.	Data on detection assay not reported
82	Hendaus, 2014	Does cesarean section pose a risk of respiratory syncytial virus bronchiolitis in infants and children?	> 2 years
83	Henderson, 1979	The etiologic and epidemiologic spectrum of bronchiolitis in pediatric practice	No molecular assays used
84	Hervas, 2012	Epidemiology of hospitalization for acute bronchiolitis in children: differences between RSV and non-RSV bronchiolitis.	No molecular assays used
85	Houben, 2011	Clinical prediction rule for RSV bronchiolitis in healthy newborns: prognostic birth cohort study	Not bronchiolitis
86	Howidi, 2007	The severity of respiratory syncytial virus bronchiolitis in young infants in the United Arab Emirates.	No molecular assays used
87	Huguenin, 2012	Broad respiratory virus detection in infants hospitalized for bronchiolitis by use of a multiplex RT-PCR DNA microarray system.	Age range not reported
88	Hyvarinen, 2011	Outcome after bronchiolitis depends on disease definition.	Only positive samples included
89	Jacques, 2006	Association of respiratory picornaviruses with acute bronchiolitis in French infants.	Duplicate of Janahi, 2017
90	Jacques, 2008	Human Bocavirus quantitative DNA detection in French children hospitalized for acute bronchiolitis.	> 2 years
91	Jartti, 2015	Rhinovirus-induced bronchiolitis: Lack of association between virus genomic load and short-term outcomes	Duplicate of Dumas, 2016
92	Jelić, 1990	[A bronchiolitis epidemic caused by respiratory syncytial viruses]	Full text not found
93	Jevsnik, 2016	The Role of Human Coronaviruses in Children Hospitalized for Acute Bronchiolitis, Acute Gastroenteritis, and Febrile Seizures: A 2-Year Prospective Study.	> 2 years
94	Jhawar, 2003	Severe bronchiolitis in children.	Review
95	Kabir, 2003	Evaluation of hospitalized infants and young children with bronchiolitis-a multi centre study.	Full text not found
96	Karr, 2009	Infant exposure to fine particulate matter and traffic and risk of hospitalization for RSV	No data on viral etiology searched

		bronchiolitis in a region with lower ambient air pollution.	
97	Kassis, 2009	[The burden and outcomes of acute bronchiolitis among young children hospitalized in Israel]	Full text not found
98	Kemper, 2005	Hospital readmission for bronchiolitis.	No data on viral etiology searched
99	Korppi, 2012	Upper age limit for bronchiolitis: 12 months or 6 months?	Comments
100	Korppi, 2015	Bronchiolitis: the disease of <6-month-old, <12-month-old or <24-month-old infants.	Comments
101	Kua, 2017	Systematic Review and Meta-Analysis of the Efficacy and Safety of Combined Epinephrine and Corticosteroid Therapy for Acute Bronchiolitis in Infants.	Systematic review
102	Laham, 2017	Clinical Profiles of Respiratory Syncytial Virus Subtypes A AND B Among Children Hospitalized with Bronchiolitis.	Duplicate of Dumas, 2016
103	Lanari, 2015	Prenatal tobacco smoke exposure increases hospitalizations for bronchiolitis in infants.	No data on viral etiology searched
104	Lanari, 2015	Risk factors for bronchiolitis hospitalization during the first year of life in a multicenter Italian birth cohort.	No data on viral etiology searched
105	Lanari, 2016	Exposure to vehicular traffic is associated to a higher risk of hospitalization for bronchiolitis during the first year of life.	Full text not found
106	Legg, 2005	Frequency of detection of picornaviruses and seven other respiratory pathogens in infants	Not bronchiolitis
107	Lin, 2009	[Detection of human metapneumovirus and human bocavirus in children with bronchiolitis in east Guangdong area]	Full text not found
108	López-Huertas, 2005	Two RT-PCR based assays to detect human metapneumovirus in nasopharyngeal aspirates	Not bronchiolitis
109	Lowther, 2000	Bronchiolitis-associated hospitalizations among American Indian and Alaska Native children.	No data on viral etiology searched
110	Luo, 2014	A systematic review of predictive modeling for bronchiolitis.	Systematic review
111	Mação, 2011	[Acute bronchiolitis: a prospective study]	Data on detection assay not reported
112	Macfarlane, 2005	RSV testing in bronchiolitis: which nasal sampling method is best?	No molecular assays used
113	Mandal, 2017	Bronchiolitis: Comparative Study between Respiratory Synctial Virus	Comments
114	Mandal, 2017	Bronchiolitis: Comparative Study between Respiratory Synctial Virus (RSV) and Non RSV Aetiology.	Comments
115	Mansbach, 2008	Prospective multicenter study of the viral etiology of bronchiolitis in the emergency department	Not possible to extract data on viral etiology searched
116	Mansbach, 2012	Prospective multicenter study of viral etiology and hospital length of stay in children with severe bronchiolitis	Duplicate of Dumas, 2016
117	Mansbach, 2016	Children Hospitalized with Rhinovirus Bronchiolitis Have Asthma-Like Characteristics.	Duplicate of Dumas, 2016

118	Mansbach, 2016	Respiratory syncytial virus and rhinovirus severe bronchiolitis are associated with distinct nasopharyngeal microbiota.	Comments
119	Mansbach, 2018	Haemophilus-dominant nasopharyngeal microbiota is associated with delayed clearance of respiratory syncytial virus in infants hospitalized for bronchiolitis	Duplicate of Mansbach, 2016
120	Mansbach, 2019	Association between rhinovirus species and nasopharyngeal microbiota in infants with severe bronchiolitis.	Duplicates
121	Mansbach, 2020	Detection of respiratory syncytial virus or rhinovirus weeks after hospitalization for bronchiolitis and the risk of recurrent wheezing.	Duplicates
122	Marguet, 2009	In very young infants severity of acute bronchiolitis depends on carried viruses.	Combination of multiple asays for detection
123	McCuskee, 2014	Bronchiolitis and pneumonia requiring hospitalization in young first nations children in Northern Ontario, Canada.	Not possible to extract data on viral etiology searched
124	McErlean, 2007	Characterisation of a newly identified human rhinovirus, HRV-QPM, discovered in infants with bronchiolitis.	> 2 years
125	McNally, 2014	Vitamin D receptor (VDR) polymorphisms and severe RSV bronchiolitis: a systematic review and meta-analysis.	Systematic review
126	McNamara, 2007	Impact of human metapneumovirus and respiratory syncytial virus co-infection in severe bronchiolitis.	Age range not reported
127	Meissner, 2016	Viral Bronchiolitis in Children.	Comments
128	Meissner, 2016	More on Viral Bronchiolitis in Children.	Comments
129	Meissner, 2016	Viral Bronchiolitis in Children.	Review
130	Mikalsen, 2012	The outcome after severe bronchiolitis is related to gender and virus.	Only positive samples included
131	Miron, 2010	Sole pathogen in acute bronchiolitis: is there a role for other organisms apart from respiratory syncytial virus?	Combination of multiple asays for detection
132	Molinari Such, 2005	Respiratory syncytial virus-related bronchiolitis in Puerto Rico.	Only positive samples included
133	Munoz-Quiles, 2016	Population-based Analysis of Bronchiolitis Epidemiology in Valencia, Spain.	No data on viral etiology searched
134	Murray, 2014	Risk factors for hospital admission with RSV bronchiolitis in England: a population-based birth cohort study.	No data on viral etiology searched
135	Naja, 2019	Bronchiolitis Admissions in a Lebanese Tertiary Medical Center: A 10 Years' Experience.	Inappropriate study population
136	Najioullah, 2020	Seasonality and coinfection of bronchiolitis: epidemiological specificity and consequences in terms of prophylaxis in tropical climate.	Inappropriate detection assay
137	Narbona-Lopez, 2018	Prevention of syncytial respiratory virus infection with palivizumab: descriptive and comparative analysis after 12 years of use.	Full text not found

138	Nenna, 2017	Modifiable risk factors associated with bronchiolitis.	No data on viral etiology searched
139	Neves Barreira, 2001	[Relationship between respiratory syncytial virus subtype and clinical severity in bronchiolitis]	Full text not found
140	Nicolai, 2013	Viral bronchiolitis in children: a common condition with few therapeutic options.	Review
141	O'Connor, 2013	The changing epidemiology of the bronchiolitis epidemic in Tallaght Hospital.	Full text not found
142	Oliveira-Santos, 2016	Influence of meteorological conditions on RSV infection in Portugal	No molecular assays used
143	Ong, 2001	A comparison of nested polymerase chain reaction and immunofluorescence for the diagnosis of respiratory infections in children with bronchiolitis, and the implications for a cohorting strategy	No molecular assays used
144	Ozkaya-Parlakay, 2019	Viral Etiology of Bronchiolitis Among Pediatric Patients.	Inappropriate study design
145	Papadopoulos, 2004	Does respiratory syncytial virus subtype influences the severity of acute bronchiolitis in hospitalized infants?	Only positive samples included
146	Paranhos-Baccalà, 2008	Mixed respiratory virus infections	Review
147	Paul, 2017	Respiratory-syncytial-virus- and rhinovirus-related bronchiolitis in children aged <2 years in an English district general hospital.	Not possible to extract data on viral etiology searched
148	Perrin, 1986	[Predictive indicators of the severity of bronchiolitis caused by respiratory syncytial virus in infants]	Full text not found
149	Pichler, 2000	Severe adenovirus bronchiolitis in children.	Case report
150	Pickles, 2015	Respiratory syncytial virus (RSV) and its propensity for causing bronchiolitis.	Review
151	Piedimonte, 2014	Respiratory syncytial virus infection and bronchiolitis.	Review
152	Prais, 2003	Admission to the intensive care unit for respiratory syncytial virus bronchiolitis: a national survey before palivizumab use.	No molecular assays used
153	Pruikkonen, 2014	Infants under 6 months with bronchiolitis are most likely to need major medical interventions in the 5 days after onset.	No molecular assays used
154	Rahbarimanesh, 2018	Viral Aetiology of Bronchiolitis in Hospitalised Children in a Tertiary Center in Tehran.	No molecular assays used
155	Rahbarimanesh, 2018	Viral Aetiology of Bronchiolitis in Hospitalised Children in a Tertiary Center in Tehran.	Inappropriate detection assay
156	Ralston, 2009	Incidence of apnea in infants hospitalized with respiratory syncytial virus bronchiolitis: a systematic review.	Systematic review
157	Ramagopal, 2016	Demographic, Clinical and Hematological Profile of Children with Bronchiolitis: A Comparative Study between Respiratory Synctial Virus [RSV] and [Non RSV] Groups.	> 2 years
158	Rivera-Sepulveda, 2017	Epidemiology of bronchiolitis: a description of emergency department visits and hospitalizations in Puerto Rico, 2010-2014.	No data on viral etiology searched

159	Rodl, 2012	Prospective evaluation of clinical scoring systems in infants with bronchiolitis admitted to the intensive care unit.	No data on viral etiology searched
160	Rodriguez-Fernandez, 2017	Respiratory Syncytial Virus Genotypes, Host Immune Profiles, and Disease Severity in Young Children Hospitalized With Bronchiolitis.	Only positive samples included
161	Rodriguez-Martinez, 2018	Predictors of prolonged length of hospital stay for infants with bronchiolitis.	No molecular assays used
162	Rossi, 2016	Viral Bronchiolitis in Children.	Comments
163	Ryu, 2015	Etiology and Outcome of Diffuse Acute Infectious Bronchiolitis in Adults.	Combination of multiple asays for detection
164	Saijo, 1994	The role of respiratory syncytial virus in acute bronchiolitis in small children in northern Japan	No molecular assays used
165	Sala, 2015	Factors associated with disease severity in children with bronchiolitis.	No data on viral etiology searched
166	Sanchez-Luna, 2016	Trends in respiratory syncytial virus bronchiolitis hospitalizations in children less than 1 year: 2004-2012.	No data on viral etiology searched
167	Sarkar, 2018	Comparative Study between Noninvasive Continuous Positive Airway Pressure and Hot Humidified High-flow Nasal Cannulae as a Mode of Respiratory Support in Infants with Acute Bronchiolitis in Pediatric Intensive Care Unit of a Tertiary Care Hospital.	No data on viral etiology searched
168	Scagnolari, 2012	Evaluation of viral load in infants hospitalized with bronchiolitis caused by respiratory syncytial virus.	Only positive samples included
169	Schaller, 2017	Bronchiolitis in Infants and Children.	Review
170	Semple, 2005	Dual infection of infants by human metapneumovirus and human respiratory syncytial virus is strongly associated with severe bronchiolitis.	Age range not reported
171	Semple, 2011	Household tobacco smoke and admission weight predict severe bronchiolitis in infants independent of deprivation: prospective cohort study.	No molecular assays used
172	Shadman, 2012	50 years ago in The Journal of Pediatrics: Observations on the etiology of acute bronchiolitis in infants.	Review
173	Shang, 2014	Elective cesarean delivery as a predisposing factor of respiratory syncytial virus bronchiolitis in children	> 2 years
174	Shay, 2001	Bronchiolitis-associated mortality and estimates of respiratory syncytial virus-associated deaths among US children, 1979-1997.	No data on viral etiology searched
175	Shmueli, 2017	Real-life comparison of three general paediatric wards showed similar outcomes for children with bronchiolitis despite different treatment regimens.	No molecular assays used
176	Simon, 2007	Detection of bocavirus DNA in nasopharyngeal aspirates of a child with bronchiolitis.	Case report
177	Sloan, 2013	Spatiotemporal patterns of infant bronchiolitis in a Tennessee Medicaid population.	No data on viral etiology searched
178	Smyth, 2002	Respiratory syncytial virus bronchiolitis: disease severity, interleukin-8, and virus genotype.	Only positive samples included

179	Soo, 2017	Pulmonary hemorrhage as a complication of Respiratory Syncyntial Virus (RSV) bronchiolitis.	Case report
180	Stempel, 2009	Multiple viral respiratory pathogens in children with bronchiolitis.	No data on viral etiology searched
181	Stevenson, 2016	Prenatal Versus Postnatal Tobacco Smoke Exposure and Intensive Care Use in Children Hospitalized With Bronchiolitis.	Duplicate of Dumas, 2016
182	Stewart, 2019	Association of respiratory viruses with serum metabolome in infants with severe bronchiolitis.	Duplicates
183	Stollar, 2014	Virologic testing in bronchiolitis: does it change management decisions and predict outcomes?	Combination of multiple assays for detection
184	Tecu, 2006	The viral bronchiolites diagnosis in children by PCR multiplex	Full text not found
185	Tecu, 2012	The adenoviral infections in children admitted to hospital with pneumonia, acute bronchiolitis or respiratory viral infections.	Full text not found
186	Toivonen, 2019	Association between rhinovirus species and nasopharyngeal microbiota in infants with severe bronchiolitis	Duplicate of Mansbach, 2016
187	Tortora, 2015	Adenovirus species C detection in children under four years of age with acute bronchiolitis or recurrent wheezing.	> 2 years
188	Toyoshima, 2011	Bronchiolitis caused by pandemic influenza A (H1N1) 2009.	Case report
189	Tsolia, 2003	Epidemiology of respiratory syncytial virus bronchiolitis in hospitalized infants in Greece.	No molecular assays used
190	Tumba, 2020	Temporal trend of hospitalizations for acute bronchiolitis in infants under one year of age in brazil between 2008 and 2015.	No data on respiratory viruses prevalence
191	Valdivia, 1997	Analysis of respiratory syncytial virus in clinical samples by reverse transcriptase-polymerase chain reaction restriction mapping	Age range not reported
192	Van Rostenberghe, 2006	RSV and bronchiolitis.	Comments
193	van Woensel, 2002	Bronchiolitis hospitalisations in the Netherlands from 1991 to 1999.	No data on viral etiology searched
194	Vicente, 1978	[Etiologic importance of the respiratory syncytial virus in bronchiolitis]	Full text not found
195	Wall, 2016	Viral Bronchiolitis in Children.	Comments
196	Werno, 2004	Human metapneumovirus in children with bronchiolitis or pneumonia in New Zealand.	> 2 years
197	Willson, 2003	Complications in infants hospitalized for bronchiolitis or respiratory syncytial virus pneumonia.	Data on detection assay not reported
198	Wolfler, 2018	The infant with severe bronchiolitis: from HFNC to CPAP and mechanical ventilation.	Review
199	Wright, 2002	Illness severity, viral shedding, and antibody responses in infants hospitalized with bronchiolitis caused by respiratory syncytial virus.	Only positive samples included
200	Yitshak-Sade, 2017	Air Pollution and Hospitalization for Bronchiolitis among Young Children.	No data on viral etiology searched

201	Yi-Wei, 2018	A molecular epidemiological study of respiratory syncytial virus circulating in southern Zhejiang Province, China, from 2009 to 2014.	Inappropriate detection assay
202	Yorita, 2007	Severe bronchiolitis and respiratory syncytial virus among young children in Hawaii.	Data on detection assay not reported
203	Zhang, 2017	[Molecular biological and clinical characteristics of respiratory syncytial virus in children with bronchiolitis]	Full text not found
