# Supplementary material for: Systematic review and meta-analysis of the prevalence of common respiratory viruses in children < 2 years with bronchiolitis in the pre-COVID-19 pandemic era
Source: PLoS One. 2020 Nov 12;15(11):e0242302. doi: 10.1371/journal.pone.0242302 (PMC7660462; doi:10.1371/journal.pone.0242302)
Supplement: S1 File — (ZIP) [file pone.0242302.s002.zip › S5 Table.pdf]

S5 Table. Individual characteristics of included studies

Name, Year	Male (%)	Age	Age_Range	Period	Design	Sampling	Timing	Country	Language	Bias	Setting	Case definition	Sample_type	Virus_Searched	Viral_diagnostic_method
Amat, 2014	59	4.05 months ± 2.79	< 1 Year	Oct/2011-May/2012	Cross sectional	Consecutive	Prospective	France	English	Moderate risk	Outpatients	Bronchiolitis was diagnosed by the presence of a recent history of an upper respiratory tract infection followed by onset of respiratory distress with cough, tachypnea, retraction and diffuse crackles, or wheezing on auscultation, in accordance with the international recommendations on the diagnosis and management of acute bronchiolitis [Subcommittee on diagnosis and management of bronchiolitis, 2006].	Nasopharyngeal secretions	HRSV; HMPV; RV; HAdV; HBoV; HPIV; HCoV; EV	Real-time PCR
Antunes, 2010	Not reported	Not reported	< 2 Years	Nov/2007-Apr/2008	Cross sectional	Consecutive	Prospective	Portugal	English	Moderate risk	Hospitalized	The same AAP definition was adopted by all hospitals.	Nasopharyngeal secretions	HRSV; HMPV; Influenza; RV; HAdV; HBoV; HPIV; HCoV; EV	Real-time PCR
Arabzadeh, 2008	Not reported	Not reported	< 2 Years	2006	Cross sectional	Consecutive	Prospective	Iran	Non english	Moderate risk	Not reported	Not reported	Nasopharyngeal secretions	HRSV; HAdV; HPIV	PCR
Atay, 2020	59.4	9±2.4 months	< 2 Years	Sep/2011-Apr/2012	Cross sectional	Consecutive sampling	Prospectively	Turkey	English	Moderate risk of bias	Hospitalized/Outpatients	The diagnosis of bronchiolitis was based primarily on each patient's history of preceding viral upper respiratory tract infections, restlessness, cough and/or rhinorrhea with signs of a respiratory illness including tachypnoea, intercostal and/or subcostal retractions, accessory muscle use, nasal flaring, grunting, color changes or apnea, wheezing or crackles, and lower oxygen saturations [7].	Nasopharyngeal secretions	HAdV; HCoV; HMPV; HPIV; HRSV; Influenza; RV	Multiplex PCR
Azkur, 2014	63.6	6.98 ± 5.53 months	< 2 Years	Nov/2011-May/2012	Cross sectional	Consecutive	Prospective	Turkey	English	Low risk	Hospitalized	Bronchiolitis is defined as a constellation of clinical symptoms and signs including a viral upper respiratory prodrome followed by increased respiratory effort and wheezing in children less than 2 years of age4.	Nasopharyngeal secretions	HRSV; HMPV; Influenza; RV; HAdV; HBoV; HPIV; HCoV; EV	Real-time multiplex PCR
Bamberger, 2012	62	17.5 ± 16.6 weeks	< 2 Years	Dec/2005-Mar/2006	Cross sectional	Consecutive	Prospective	Israel	English	Low risk	Hospitalized	Acute AB was diagnosed clinically in those subjects with upper respiratory tract infections (URTIs) with cough and by at least two of the following signs: chest retractions, tachypnea, wheezing, or rales on auscultation.	Nasopharyngeal secretions	HRSV; HMPV; Influenza; RV; HAdV; HBoV; HPIV	PCR
Bekhof, 2013	54,2	3.2 months	< 2 Years	Dec/2011-Mar/2012	Cross sectional	Consecutive	Prospective	Netherlands	English	Low risk	Hospitalized	Bronchiolitis was defined as acute respiratory disease, accompanied by coryza, cough, inspiratory crackles and/or expiratory wheezing on auscultation.	Nasopharyngeal secretions	HRSV; HMPV; RV; HAdV; HCoV	PCR
Bekhof, 2019	59	4.9 (1.6–7.0) mo	< 2 Years	Dec-Mar; 2012-2016	Cohort (Baseline data)	Consecutive sampling	Prospectively	Netherlands	English	Moderate risk of bias	Hospitalized	Bronchiolitis was defined as acute respiratory disease accompanied by coryza, cough, and inspiratory crackles and/or expiratory wheezing on auscultation.	Nasopharyngeal secretions	EV; HAdV; HCoV; HMPV; HPIV; HRSV; Influenza; RV	Real-time PCR
Brand, 2012	61	4.5 ± 0.39 (months)	< 2 Years	2006-2009 (Nov-Apr)	Cross sectional	Consecutive	Prospective	Pays-Bas	English	Low risk	Hospitalized/Outpatients	Bronchiolitis was defined as an acute infection of the lower airways, characterized by increased respiratory effort (tachypnea and/or use of accessory respiratory muscles) and expiratory wheezing and/or crackles and/or apnea.	Nasopharyngeal secretions	HRSV; HMPV; Influenza; RV; HAdV; HBoV; HPIV; HCoV; EV	PCR
Calvo, 2010	55	5.6 ± 5.7 months	< 2 Years	Sept/2005-Aug/2008	Cross sectional	Consecutive	Prospective	Spain	English	Low risk	Hospitalized	Acute bronchiolitis, was diagnosed as the first episode of acute onset expiratory dyspnea with previous signs of viral respiratory infection – whether or not this was associated to respiratory distress or pneumonia in children under 2 years old.	Nasopharyngeal secretions	HRSV; HMPV; Influenza; RV; HAdV; HBoV; HPIV; HCoV; EV	PCR
Cangiano, 2016	54.6	Median age 2.13 months	< 1 Year	2004-2014 (Oct-May)	Cross sectional	Consecutive	Prospective	Italy	English	Low risk	Hospitalized	Acute bronchiolitis was clinically defined as the first episode of acute lower respiratory tract infection in infants less than 12 months, characterized by the acute onset of cough, tachypnoea, retraction, and diffuse crackles on chest auscultation.9	Nasopharyngeal secretions	HRSV; HMPV; Influenza; RV; HBoV; HPIV; HCoV	PCR
Chen, 2014	67.3	Mean 255.6 days; 95% CI 240.4-270.9 days	< 2 Years	Jan/2009-Dec/2010	Cross sectional	Consecutive	Prospective	China	English	Low risk	Hospitalized	Acute bronchiolitis was defined as the first episode of cough, rhinorrhea, wheezing, or rales, with chest radiographic findings of over-aeration and peribronchial infiltration with or without atelectasis in children younger than 2 years.	Nasopharyngeal secretions	HMPV; HBoV	PCR
Dong-Keon, 2018	Unclear/Not repor	2.8 ± 2.4 months	< 1 Year	Nov/2016-Feb/2017	Cross sectional	Consecutive sampling	Retrospectively	North Korea	Non english	Moderate risk of bias	Hospitalized	Unclear/Not reported	Nasopharyngeal secretions	HRSV	Real-time PCR
Dumas, 2016	61	8.1 months (SD: 6.7)	< 2 Years	2007-2010 (Nov-Mar)	Cross sectional	Consecutive	Prospective	Finlande	English	Low risk	Hospitalized	Bronchiolitis (as defined by the American Academy of Pediatrics:28 acute respiratory illness with some combination of rhinitis, cough, tachypnoea, wheezing, crackles and retractions), and were younger than 2 years.	Nasopharyngeal secretions	HRSV; RV	PCR
Dumas, 2016	59	5.8 months (SD: 5.2)	< 2 Years	2007-2010 (Nov-Mar)	Cross sectional	Consecutive	Prospective	USA	English	Low risk	Hospitalized	Bronchiolitis (as defined by the American Academy of Pediatrics:28 acute respiratory illness with some combination of rhinitis, cough, tachypnoea, wheezing, crackles and retractions), and were younger than 2 years.	Nasopharyngeal secretions	HRSV; RV	PCR
Ebihara, 2005	Not reported	Not reported	< 2 Years	Oct/2002-Sep/2003	Cross sectional	Consecutive	Prospective	Japan	English	Moderate risk	Hospitalized	Not reported	Nasopharyngeal secretions	HCoV	PCR
Gokce, 2017	63.6	7 ± 6.5 months	< 2 Years	Oct/2013-Oct/2016	Cross sectional	Consecutive	Prospective	Turkey	English	Low risk	Hospitalized	The diagnosis of AB was based on at least 2 of the following signs: chest retractions, tachypnea, and wheezing or rales on auscultation following viral upper respiratory tract infection in children less than 24 months of age for the first time.11	Nasal secretions	HRSV; HMPV; Influenza; RV; HAdV; HBoV; HPIV; HCoV	RT-PCR
Hendaus, 2018	60.8	3.61 ± 3.56 months	< 2 Years	Jan/2010-Dec/2012	Cross sectional	Consecutive	Retrospective	Qatar	English	Moderate risk	Hospitalized	The diagnosis of bronchiolitis was based on the definition suggested by the American Academy of pediatrics, subcommittee on diagnosis and management of bronchiolitis.1	Nasopharyngeal secretions	HRSV; RV; HAdV; HBoV; HPIV; HCoV	Real-time PCR
Janahi, 2017	67	Not reported	< 2 Years	2010-2011	Cross sectional	Consecutive	Retrospective	Qatar	English	Low risk	Hospitalized	Acute bronchiolitis defined according to International Classification of Disease (ICD) code 466.1 in the 9th revision of the ICD. Based on recorded clinical diagnosis, an episode of acute bronchiolitis was determined by a constellation of clinical signs and symptoms including fever, rhinitis, tachypnoea, cough, wheezing, crackles, use of accessory muscles and possible chest X ray findings of hyperinflation of the lungs, peri-bronchial thickening, collapsed segment or a lobe of the lung and increase interstitial markings.	Nasopharyngeal secretions	HRSV; HMPV; Influenza; RV; HAdV; HBoV; HPIV; HCoV; EV	Real-time PCR
Jartti, 2014	61	8.1 months (SD: 6.7)	< 2 Years	2007-2010 (Nov-Mar)	Cross sectional	Consecutive	Prospective	Finlande	English	Low risk	Hospitalized	Bronchiolitis (as defined by the American Academy of Pediatrics:28 acute respiratory illness with some combination of rhinitis, cough, tachypnoea, wheezing, crackles and retractions), and were younger than 2 years.	Nasopharyngeal secretions	HMPV; Influenza; RV; HAdV; HPIV; HCoV; EV	PCR
Mansbach, 2008	61	Median age of 6.3 months (IQR 3.1 to 10.2)	< 2 Years	Dec/2005-Mar/2006	Cohort	Consecutive	Prospective	USA	English	Moderate risk	Outpatients	The diagnosis of bronchiolitis was considered for children younger than 2 years with acute tachypnea, retractions, and abnormal breath sounds who did not meet criteria for a diagnosis of pneumonia or another primary diagnosis. As defined by the AAP in its 2006 position statement, children with bronchiolitis typically have ''rhinitis, tachypnea, wheezing, cough, crackles, use of accessory muscles, and ? or nasal flaring.''12	Nasopharyngeal secretions	HRSV; RV	RT-PCR
Mansbach, 2016	Not reported	Not reported	< 1 Year	2011-2014 (Nov-Apr)	Cohort	Consecutive	Prospective	USA	English	Low risk	Hospitalized	Bronchiolitis defined by the Academy of Pediatrics: acute respiratory illness with some combination of rhinitis, cough, tachypnea, wheezing, crackles, and retractions;1	Nasopharyngeal secretions	HRSV; RV	PCR
Midulla, 2010	57	2.5 ± 2.1 months	< 1 Year	2004-2007 (Oct-May)	Cross sectional	Consecutive	Prospective	Italy	English	Low risk	Hospitalized	Bronchiolitis was diagnosed according to the presence of a history of upper respiratory tract infection followed by acute onset of respiratory distress with cough, tachypnea, retraction and diffuse crackles on auscultation (having wheezing alone was not considered sufficient for inclusion in the study).18	Nasal secretions	HRSV; HMPV; Influenza; RV; HBoV; HPIV; HCoV	PCR
Midulla, 2018	Not reported	Not reported	< 1 Year	2005-2017 (Sep-May)	Cross sectional	Consecutive	Prospective	Italy	English	Low risk	Hospitalized	Bronchiolitis was defined as the first episode of acute lower respiratory tract infection in infants less than 1 year old, characterized by the acute onset of cough, tachypnoea, retraction, and diffuse crackles (wheezing alone was insufficient for inclusion) [28].	Nasopharyngeal secretions	HRSV	PCR
Miller, 2013	Not reported	Not reported	< 1 Year	2004-2008 (Sept-May)	Cohort	Consecutive	Prospective	USA	English	Low risk	Hospitalized/Outpatients	Bronchiolitis was defined as lower respiratory infection in infants presenting with wheezing, rales, and respiratory distress.	Nasopharyngeal secretions	HRSV; HMPV; Influenza; RV; HPIV; HCoV	PCR
Nascimento, 2010	65	Median age was 6 ± 4 months	< 2 Years	Mar/2006-Jul/2007	Cross sectional	Consecutive	Prospective	Brazil	English	Low risk	Outpatients	Bronchiolitis was defined as the first episode of wheezing associated with signs of an upper respiratory tract infection.	Nasopharyngeal secretions	HRSV; HMPV; Influenza; RV; HAdV; HBoV; HPIV; HCoV; EV	PCR, RT-PCR, Real-time RT-PCR
Nenna, 2020	Unclear/Not repor	Unclear/Not reported	< 1 Year	Unclear/Not reported	Cross sectional	Consecutive sampling	Prospectively	Italy	English	Moderate risk of bias	Hospitalized	Bronchiolitis was defined as the first episode of acute lower airway infection, characterized by a history of upper respiratory tract infection followed by acute onset of respiratory distress with cough, tachypnoea, retraction, and diffuse crackles on auscultation.	Nasopharyngeal secretions	HBoV; HMPV; HRSV; RV	Classical PCR
Nenna, 2020	Unclear/Not repor	Unclear/Not reported	< 1 Year	Unclear/Not reported	Cross sectional	Consecutive sampling	Prospectively	Italy	English	Moderate risk of bias	Hospitalized	Bronchiolitis was defined as the first episode of acute lower airway infection, characterized by a history of upper respiratory tract infection followed by acute onset of respiratory distress with cough, tachypnoea, retraction, and diffuse crackles on auscultation.	Nasopharyngeal secretions	HBoV; HMPV; HRSV; RV	Classical RT-PCR
Papadopoulos, 2002	61.3	3.9 months	< 1.5 years	Oct/1999-Sep/2000	Cross sectional	Consecutive	Prospective	Greece	English	Low risk	Hospitalized	Bronchiolitis was defined as an acute infection of the lower airway characterized by increased respiratory effort (tachypnea [more than 50 respirations/minute] and/or use of accessory respiratory muscles) and expiratory wheezing and/or crackles.	Nasopharyngeal secretions	HRSV; Influenza; RV; HAdV; HPIV; HCoV	PCR
Papoff, 2011	50.5	Median (interquartile. I.Q.) were 61 days (I.Q. 33–99 days)	< 1 Year	2004-2009 (Oct-May)	Cross sectional	Consecutive	Prospective	Italy	English	Low risk	Hospitalized	Bronchiolitis was diagnosed clinically according to the presence of a history of upper respiratory tract infection followed by acute onset of respiratory distress with cough, tachypnea, retractions and bilateral crackles on auscultation having wheezing alone was not considered sufficient for inclusion in the study (11).	Nasal secretions	HRSV; HMPV; Influenza; RV; HBoV; HPIV; HCoV	PCR
Petrarca, 2018	54.1	Median age 2.03 months. range: 0.23-11.17	< 1 Year	2004-2016 (Oct-May)	Cross sectional	Consecutive	Retrospective	Italy	English	Low risk	Hospitalized	Bronchiolitiswas clinically defined as the first episode of acute lower respiratory tract infection, characterized by the acute onset of cough, tachypnea, retraction, and diffuse crackles on chest auscultation in infants younger than 12 months.13	Nasopharyngeal secretions	HRSV; RV	PCR
Piedra, 2017	60.8	6.7±6.3 months	< 2 Years	Oct/2010-Apr/2011	Cross sectional	Consecutive	Prospective	USA	English	Low risk	Outpatients	Bronchiolitis was defined as a physician diagnosis in children less than 24 months of age with wheezing and/or rales who had a history of preceding upper airway illness.	Nasopharyngeal secretions	HRSV	Real-time PCR
Pitrez, 2005	56	Median 2 (1.2-3.6) months	< 0.5 year	May/2012-Sep/2012	Cohort	Consecutive	Prospective	Brazil	Non english	Moderate risk	Hospitalized	BA was defined as the first episode of wheezing, associated with signs and symptoms of acute viral infection (coryza and cough).	Nasopharyngeal secretions	RV	RT-PCR
Praznik, 2018	61,5	Not reported	< 2 Years	May/2014-Oct/2008	Cross sectional	Consecutive	Retrospective	Slovenia	English	Moderate risk	Hospitalized/Outpatients	Patients were identified through a query of the electronic medical record based on a bronchiolitis diagnosis (ICD-10 code J21.0-9).	Nasopharyngeal secretions	HRSV; RV; HBoV	RT-PCR
Ricart, 2013	Not reported	Not reported	< 1 Year	Sep/2007-Oct/2008	Cross sectional	Consecutive	Prospective	Spain	English	Low risk	Hospitalized	Patients were admitted if one of the following criteria was present: pulse oximetry <92% on room air, moderate bronchiolitis (according to bronchiolitis clinical score),5 poor feeding with risk of dehydration, apnea, significant congenital heart disease or pulmonary bronchodysplasia, or infant aged <4 weeks.	Nasopharyngeal secretions	HRSV; HMPV; Influenza; RV; HAdV; HBoV; HPIV; HCoV; EV	PCR
Ricart, 2013	Not reported	Not reported	< 1 Year	Oct/2007-Oct/2008	Cross sectional	Consecutive	Prospective	Spain	English	Low risk	Hospitalized	Bronchiolitis was diagnosed according to the presence of the first acute respiratory tract infection characterized by respiratory distress (tachypnea, use of accessory muscles), cough, widespread crackles, wheezing or both, associated with signs of viral infection (coryza).2	Nasopharyngeal secretions	HRSV; HMPV; Influenza; RV; HAdV; HBoV; HPIV; HCoV; EV	RT-PCR, Real-time RT-PCR
Ricart, 2014	60.8	41.7 (13-70.3) (Mean (95% confidence interval) days)	< 1 Year	Jan/2006-Oct/2008	Cross sectional	Consecutive	Prospective	Spain	English	Low risk	Hospitalized	Bronchiolitis was diagnosed according to the presence of the first acute respiratory tract infection characterized by respiratory distress, cough, widespread crackles or wheezing or both, associated with signs of viral infection (coryza).	Nasopharyngeal secretions	HRSV; HMPV; RV; HAdV; HCoV	PCR
Richard, 2008	Not reported	Not reported	< 1 Year	2003-2005 (Sep-Apr)	Cross sectional	Consecutive	Retrospective	France	English	Moderate risk	Hospitalized	Not reported	Nasopharyngeal secretions	HRSV; Influenza; RV; HAdV; HPIV; EV	PCR ou NASBA
Robledo-Aceves, 2018	60.5	6.60 ± 5.7 months	< 2 Years	Sep/2012-Jan/2015	Case-control	Consecutive	Retrospective	Mexico	English	Low risk	Hospitalized	Severe bronchiolitis was defined as rhinorrhea, cough, tachypnea, wheezing, rales, and increased respiratory effort (e.g., grunting, nasal flaring, and intercostal and/or subcostal retraction), with symptoms of severity (e.g., increased respiratory rate, retractions, and oxygen saturation at 90% or lower).[25]	Nasopharyngeal secretions	HRSV; HMPV; Influenza; RV; HBoV; HPIV; HCoV	RT-PCR, PCR
Salvador García, 2012	53.6	3.4 (± 3.3) months	< 1.5 years	Dec/2008-Apr-2009	Cross sectional	Consecutive	Prospective	Spain	Non english	Low risk	Hospitalized	The diagnosis of bronchiolitis was established according to the McConnochie criteria7, defined as the first acute episode of respiratory distress with wheezing associated with catarrhal pathology in children under 2 years of age.	Nasopharyngeal secretions	HRSV; HMPV; Influenza; RV; HAdV; HBoV; HPIV; HCoV; EV	RT-PCR
Souza, 2016	Not reported	Not reported	< 1 Year	Sep/2010-Aug/2011	Cross sectional	Consecutive	Prospective	Brazil	English	Low risk	Hospitalized	Acute bronchiolitis (based on prodromal symptoms, with wheezing, crackles, and tachypnea); having been recruited within the first 48 h of hospitalization; and having a ? 72-h history of clinical manifestations of lower respiratory tract infection (wheeze or respiratory distress).	Nasopharyngeal secretions	HRSV; HMPV; RV	Real-time PCR
Stein, 2019	53.5	4 months	< 2 Years	2016–2018	Cross sectional	Consecutive sampling	Prospectively	Greece	English	Moderate risk of bias	Hospitalized	Unclear/Not reported	Nasopharyngeal secretions	HRSV	Classical RT-PCR
Su-Jin, 2018	62.6	Unclear/Not reported	< 2 Years	Nov/2007-May/2015	Cross sectional	Consecutive sampling	Prospectively	South Korea	Non english	Low risk of bias	Hospitalized	Bronchiolitis was defined as a clinical syndrome accompanied by symptoms of upper respiratory tract infection and lower respiratory tract infection such as wheezing.	Nasopharyngeal secretions	EV; HAdV; HBoV; HCoV; HMPV; HPIV; HRSV; Influenza; RV	Real-time PCR
Teeratakulpisarn, 2007	Not reported	Not reported	< 2 Years	Apr/2002-Aug/2004	Cross sectional	Consecutive	Prospective	Thailand	English	Low risk	Hospitalized	Bronchiolitis was defined as the first epi-sode of wheezing associated with tachypnea, in-creased respiratory effort and an upper respiratory tract infection.	Nasopharyngeal secretions	HRSV; HMPV	RT-PCR
Tsou, 2020	57.4	Unclear/Not reported	< 1 Year	Jan/2015-Dec/2017	Case control	Simple random sampling	Prospectively	United States of America	English	Low risk of bias	Hospitalized	The study sample was chosen based on the ICD code for bronchiolitis during the study period.	Nasopharyngeal secretions	HAdV; HCoV; HMPV; HPIV; HRSV	Real-time PCR
Uyar, 2014	72.5	7.18 ± 5.75 months	< 2 Years	Jan/2010-Jun/2010	Cross sectional	Consecutive	Prospective	Turkey	Non english	Low risk	Outpatients	Diagnosis of acute bronchiolitis; upper respiratory tract infect nasal discharge, cough, mild fever, agitation difficulty eating, wheezing, increased respiratory rate, chest weaning was performed according to the criteria of expiration time and wheezing.	Nasopharyngeal secretions	HRSV; HMPV; Influenza; RV; HAdV; HBoV; HPIV; EV	Multiplex PCR
Vieira, 2017	48	2.4 months (SD = 1.6)	< 0.5 year	2013-2015	Cross sectional	Consecutive	Prospective	Brazil	English	Moderate risk	Hospitalized	Diagnosis of bronchiolitis was defined as the first wheezing crisis, beginning no more than 3 days before hospital admission.	Nasal secretions	HRSV	Real-time PCR
Wang, 2015	68,4	Not reported	< 2 Years	Jan/2010-Dec/2013	Cross sectional	Consecutive	Prospective	China	English	Low risk	Hospitalized	The bronchiolitis was diagnosed based on the guidelines described by Zhu Futang Practical Pediatrics, 7th Edition (12).	Nasal secretions	HMPV	RT-PCR, Real-time RT-PCR
Wollmeister, 2018	Unclear/Not repor	Unclear/Not reported	< 1 Year	2004; 2014	Cross sectional	Consecutive sampling	Prospectively	Brazil	English	Moderate risk of bias	Hospitalized	Diagnosis was based on clinical data, which defines AVB as being the first episode of acute respiratory distress with wheezing, preceded by upper airway symptoms such as rhinorrhea and cough, with or without fever [1,5]. The criterion for severe bronchiolitis was oxygen saturation lower than 92%, which demanded oxygen therapy [1].	Nasopharyngeal secretions	HAdV; HBoV; HCoV; HMPV; HPIV; HRSV; Influenza; RV	Classical PCR
Xepapadaki, 2004	Not reported	Not reported	< 1.5 years	Oct/1999-Sep/2000	Cross sectional	Consecutive	Prospective	Greece	English	Low risk	Hospitalized	Bronchiolitis, which was defined as an acute infection of the lower airway, characterized by increased respiratory effort (tachypnea of >50 respirations/min and/or use of accessory respiratory muscles), and expiratory wheezing and/or crackles.	Nasopharyngeal secretions	HMPV	PCR
Xuan, 2018	Unclear/Not repor	Unclear/Not reported	Unclear/Not reported	Jan/2015-Dec/2016	Cross sectional	Consecutive sampling	Prospectively	China	Non english	Moderate risk of bias	Unclear/Not reported	Unclear/Not reported	Throat secretions	HBoV; HRSV	Classical PCR
Zhou, 2015	Not reported	Not reported	< 2 Years	2012-2014 (winter)	Cross sectional	Consecutive	Prospective	China	English	Low risk	Hospitalized	The diagnosis of bronchiolitis is according to the Zhu Futang textbook of Pediatrics [Zhang, 2002]. Bronchiolitis was diagnosed from the presence of a history of upper respiratory tract infection followed by acute onset of respiratory distress with cough, tachypnea, retraction, wheezing, and diffuse crackles on auscultation.	Nasopharyngeal secretions	HRSV	PCR
