# Supplementary material for: Systematic review and meta-analysis of the prevalence of common respiratory viruses in children < 2 years with bronchiolitis in the pre-COVID-19 pandemic era
Source: PLoS One. 2020 Nov 12;15(11):e0242302. doi: 10.1371/journal.pone.0242302 (PMC7660462; doi:10.1371/journal.pone.0242302)
Supplement: S1 File — (ZIP) [file pone.0242302.s002.zip › S6 Table.pdf]

S6 Table. Global prevalence and sensitivity analyses of respiratory viral infections in children < 2 years with bronchiolitis

	**Prevalence [95% CI]**	**95% Prediction interval**	**N Studies**	**N Cases**	**H [95% CI]**	**I2 [95% CI]**	**P-Value Heterogeneity**	**P-Value Egger test**
**HRSV**								
Overall	59.2 [54.7-63.6]	[29.1-85.9]	45	15351	5.5 [5.1-6]	96.8 [96.2-97.2]	< 0.001	0.415
< 1 Year	62.3 [55.7-68.6]	[32.4-87.8]	18	6779	5.4 [4.7-6.1]	96.5 [95.5-97.3]	< 0.001	0.878
Hospitalized	60.6 [55.9-65.2]	[32.8-85.2]	34	12867	5.3 [4.8-5.8]	96.4 [95.7-97]	< 0.001	0.601
Bronchiolitis definition given	60.4 [56-64.8]	[32.4-85.2]	40	14456	5.3 [4.8-5.7]	96.4 [95.7-97]	< 0.001	0.473
Cross sectional design	57.2 [52.3-62]	[27.2-84.5]	39	12992	5.4 [5-5.9]	96.6 [96-97.2]	< 0.001	0.395
Low risk of bias	59.4 [54.1-64.5]	[30.4-85.2]	30	11024	5.4 [4.9-5.9]	96.5 [95.8-97.2]	< 0.001	0.325
**RV**								
Overall	19.3 [16.7-22]	[6.3-37]	36	12967	3.7 [3.3-4.2]	92.8 [90.9-94.2]	< 0.001	0.318
< 1 Year	16.9 [13.4-20.7]	[4.8-34]	15	5382	3.4 [2.8-4.1]	91.5 [87.6-94.1]	< 0.001	0.856
Hospitalized	18 [15.1-21.1]	[5-36.4]	28	11159	4 [3.5-4.5]	93.8 [92-95.1]	< 0.001	0.142
Bronchiolitis definition given	19.2 [16.6-22]	[6.2-37.2]	35	12787	3.8 [3.4-4.2]	93 [91.2-94.4]	< 0.001	0.315
Cross sectional design	19.9 [16.9-23.2]	[5.9-39.4]	30	10833	4 [3.5-4.5]	93.6 [91.9-95]	< 0.001	0.315
Low risk of bias	21 [18-24.2]	[8-37.9]	25	9414	3.5 [3-4]	91.6 [88.9-93.7]	< 0.001	0.304
**HBoV**								
Overall	8.2 [5.6-11.2]	[0-27.3]	24	8706	4.7 [4.1-5.3]	95.4 [94.1-96.4]	< 0.001	0.748
< 1 Year	8 [2.4-16.6]	[0-49]	8	3104	7.3 [6.2-8.6]	98.1 [97.4-98.7]	< 0.001	0.864

	Prevalence [95% CI]	95% Prediction interval	N Studies	N Cases	H [95% CI]	I2 [95% CI]	P-Value Heterogeneity	P-Value Egger test
Hospitalized	8 [5-11.6]	[0-28.6]	18	7335	5.2 [4.5-5.9]	96.2 [95.1-97.1]	< 0.001	0.853
Bronchiolitis definition given	8.2 [5.5-11.3]	[0-27.8]	23	8310	4.8 [4.2-5.4]	95.6 [94.4-96.6]	< 0.001	0.753
Cross sectional design	8.3 [5.6-11.4]	[0-27.6]	23	8572	4.8 [4.2-5.4]	95.6 [94.4-96.6]	< 0.001	0.777
Low risk of bias	9.5 [6.5-13.1]	[0.3-28.2]	17	5578	4.1 [3.5-4.8]	94 [91.8-95.6]	< 0.001	0.331
**HAdV**								
Overall	6.1 [4.4-8]	[0.2-18.1]	26	6734	3 [2.6-3.5]	88.9 [85-91.8]	< 0.001	0.085
< 1 Year	6.7 [3.6-10.6]	[0-23.1]	7	1938	3 [2.2-4]	88.6 [78.9-93.8]	< 0.001	0.538
Hospitalized	6.1 [4.2-8.4]	[0.1-18.9]	20	5963	3.3 [2.8-3.9]	90.6 [87-93.3]	< 0.001	0.121
Bronchiolitis definition given	6.2 [4.4-8.2]	[0.2-18.2]	24	6386	3 [2.6-3.5]	89 [84.9-92]	< 0.001	0.097
Cross sectional design	6.4 [4.5-8.6]	[0.2-19.2]	23	6112	3.1 [2.6-3.6]	89.5 [85.6-92.4]	< 0.001	0.149
Low risk of bias	6.4 [4.2-8.9]	[0.1-20]	18	4632	3.1 [2.6-3.7]	89.7 [85.2-92.8]	< 0.001	0.052
**HPIV**								
Overall	5.4 [3.8-7.3]	[0-17.9]	28	7933	3.3 [2.9-3.8]	91 [88.1-93.2]	< 0.001	0.932
< 1 Year	3.1 [1.9-4.6]	[0.1-9.3]	10	3185	2.1 [1.6-2.9]	78.1 [60.1-88]	< 0.001	0.531
Hospitalized	5 [3.3-6.9]	[0-16.7]	21	6707	3.4 [2.9-3.9]	91.1 [87.8-93.5]	< 0.001	0.238
Bronchiolitis definition given	5 [3.4-6.8]	[0-16.7]	26	7585	3.3 [2.8-3.8]	90.6 [87.4-92.9]	< 0.001	0.682
Cross sectional design	5.3 [3.6-7.2]	[0-17.2]	24	6856	3.2 [2.8-3.7]	90.3 [86.8-92.8]	< 0.001	0.528

	Prevalence [95% CI]	95% Prediction interval	N Studies	N Cases	H [95% CI]	I2 [95% CI]	P-Value Heterogeneity	P-Value Egger test
Low risk of bias	5 [3.1-7.2]	[0-18]	20	5831	3.4 [2.9-4]	91.5 [88.3-93.9]	< 0.001	0.983
**HMPV**								
Overall	5.4 [4.4-6.4]	[1.4-11.5]	32	9908	2.1 [1.8-2.5]	76.9 [67.7-83.5]	< 0.001	0.043
< 1 Year	3.4 [2.4-4.7]	[0.5-8.4]	12	3936	1.9 [1.4-2.5]	71.4 [48.6-84.1]	< 0.001	0.633
Hospitalized	5.1 [4.2-6.1]	[1.9-9.7]	26	8850	1.8 [1.5-2.2]	68.8 [53.2-79.1]	< 0.001	0.238
Bronchiolitis definition given	5.4 [4.4-6.4]	[1.4-11.5]	32	9908	2.1 [1.8-2.5]	76.9 [67.7-83.5]	< 0.001	0.043
Cross sectional design	5.6 [4.6-6.7]	[1.9-11.1]	28	8831	1.9 [1.6-2.3]	72.7 [60.3-81.2]	< 0.001	0.04
Low risk of bias	5.7 [4.5-7]	[1.3-12.8]	26	8034	2.2 [1.9-2.7]	79.6 [70.8-85.8]	< 0.001	0.07
**Influenza**								
Overall	3.2 [2.2-4.3]	[0-10]	24	6571	2.4 [2-2.8]	82.1 [74.3-87.5]	< 0.001	0.032
< 1 Year	1.6 [0.8-2.6]	[0-5.2]	8	2694	1.7 [1.2-2.5]	65.4 [26.3-83.7]	0.005	0.422
Hospitalized	2.6 [1.8-3.6]	[0.1-7.8]	19	5734	2.1 [1.7-2.6]	77.4 [65.1-85.4]	< 0.001	0.407
Bronchiolitis definition given	3.2 [2.1-4.4]	[0-10.2]	23	6391	2.4 [2-2.9]	82.8 [75.3-88.1]	< 0.001	0.037
Cross sectional design	3 [2-4.3]	[0-10.1]	21	5764	2.4 [2-2.9]	82.8 [74.8-88.3]	< 0.001	0.083
Low risk of bias	3.2 [2.1-4.6]	[0-10.6]	19	5561	2.5 [2.1-3.1]	84.2 [76.7-89.4]	< 0.001	0.08
**HCoV**								
Overall	2.9 [2-4]	[0-9.9]	27	7431	2.5 [2.1-2.9]	83.5 [76.9-88.1]	< 0.001	0.918
< 1 Year	2.6 [1-4.8]	[0-13.3]	10	3056	3.1 [2.4-4]	89.7 [83.2-93.7]	< 0.001	0.744

	Prevalence [95% CI]	95% Prediction interval	N Studies	N Cases	H [95% CI]	I2 [95% CI]	P-Value Heterogeneity	P-Value Egger test
Hospitalized	2.6 [1.6-3.8]	[0-9.4]	22	6435	2.5 [2.1-3]	84 [76.9-88.9]	< 0.001	0.556
Bronchiolitis definition given	2.9 [2-4.1]	[0-10]	26	7313	2.5 [2.1-3]	84.1 [77.7-88.6]	< 0.001	0.931
Cross sectional design	2.6 [1.7-3.7]	[0-8.8]	23	6354	2.3 [1.9-2.8]	81.1 [72.5-87]	< 0.001	0.744
Low risk of bias	2.8 [1.8-4]	[0-9.4]	20	5559	2.3 [1.9-2.8]	81.5 [72.3-87.6]	< 0.001	0.701
**EV**								
Overall	2.9 [1.6-4.5]	[0-11.4]	15	4202	2.7 [2.2-3.3]	86.1 [78.6-90.9]	< 0.001	0.886
< 1 Year	2.2 [1.4-3]	[0.7-4.3]	4	1303	1 [1-2.3]	0 [0-80.5]	0.502	0.292
Hospitalized	2.2 [1-3.6]	[0-9.1]	11	3700	2.6 [2-3.3]	84.7 [74.3-90.9]	< 0.001	0.115
Bronchiolitis definition given	2.8 [1.4-4.6]	[0-11.7]	14	4022	2.8 [2.2-3.5]	87 [79.9-91.6]	< 0.001	0.849
Cross sectional design	2.9 [1.5-4.7]	[0-12]	14	3984	2.8 [2.2-3.5]	87 [79.9-91.6]	< 0.001	0.911
Low risk of bias	3.4 [1.7-5.6]	[0-13.5]	11	3386	2.8 [2.2-3.6]	87.7 [79.9-92.5]	< 0.001	0.863

CI: confidence interval; RV: Rhinovirus; HCoV: Human Coronavirus; HPIV: Human Parainfluenzavirus; HMPV: Human Metapneumovirus; HRSV: Human Respiratory Syncytial Virus; HAdV: Human Adenovirus; HBoV: Human Bocavirus; EV: Enterovirus; NA: not applicable.
