# Supplementary material for: Systematic review and meta-analysis of the prevalence of common respiratory viruses in children < 2 years with bronchiolitis in the pre-COVID-19 pandemic era
Source: PLoS One. 2020 Nov 12;15(11):e0242302. doi: 10.1371/journal.pone.0242302 (PMC7660462; doi:10.1371/journal.pone.0242302)
Supplement: S1 File — (ZIP) [file pone.0242302.s002.zip › S7 Table.pdf]

S7 Table. Subgroup prevalence of respiratory viral infections in children with acute bronchiolitis

	**Prevalence [95% CI]**	**95% Prediction interval**	**H [95% CI]**	**N Studies**	**N Cases**	**I2 [95% CI]**	**P-Value Heterogeneity**	**P-Value Egger test**	**P-value subgroup difference**
**HRSV**									
**Study period**									0.304
Continuous study period	57.3 [51.6-62.9]	[26.1-85.6]	4.9 [4.4-5.4]	30	7454	95.8 [94.9-96.6]	< 0.001	0.647	
Interrupted time series study	62.4 [54.3-70.2]	[28.7-90.5]	6.6 [5.8-7.5]	14	7073	97.7 [97-98.2]	< 0.001	0.622	
**WHO Region**									< 0.001
America	64 [58.2-69.6]	[42.8-82.7]	3.5 [2.8-4.4]	10	4731	92 [87.4-94.9]	< 0.001	0.104	
Eastern Mediterranean	46.9 [40.2-53.7]	[0-100]	2.2 [1.3-4]	3	1240	80 [36.8-93.7]	0.007	0.386	
Europe	59 [52.9-64.9]	[27.4-86.9]	5.3 [4.7-5.9]	26	7636	96.4 [95.6-97.1]	< 0.001	0.815	
Northern America	82.6 [77.8-86.9]	NA	NA	1	270	NA	1	NA	
South-East Asia	64.7 [57.3-71.7]	NA	NA	1	170	NA	1	NA	
Western Pacific	51.3 [32.1-70.3]	[0-100]	6.1 [4.6-8.1]	4	1304	97.3 [95.3-98.5]	< 0.001	0.705	
**Sample type**									< 0.001
Nasal secretions	50 [36.5-63.5]	[1.6-98.4]	4 [2.8-5.8]	4	902	93.9 [87.4-97]	< 0.001	0.116	
Nasopharyngeal secretions	61 [56.8-65.2]	[34-84.9]	5 [4.6-5.5]	40	14053	96 [95.2-96.6]	< 0.001	0.465	
Throat secretions	24.5 [20.4-28.9]	NA	NA	1	396	NA	1	NA	
**RV**									
**Study period**									0.391
Continuous study period	20.4 [16.9-24]	[6.3-39.6]	3.3 [2.8-3.8]	24	6199	90.8 [87.7-93.2]	< 0.001	0.314	
Interrupted time series study	18 [13.7-22.7]	[4.5-37.8]	4.2 [3.5-5.1]	11	5944	94.4 [91.8-96.2]	< 0.001	0.178	
**WHO Region**									< 0.001
America	17.8 [13.6-22.4]	[5.5-34.9]	3.2 [2.5-4.2]	9	4570	90.5 [84.2-94.3]	< 0.001	0.187	
Eastern Mediterranean	18.8 [8.3-32.3]	NA	4.9 [2.9-8.3]	2	1072	95.9 [88.4-98.6]	< 0.001	NA	

	Prevalence [95% CI]	95% Prediction interval	H [95% CI]	N Studies	N Cases	I2 [95% CI]	P-Value Heterogeneity	P-Value Egger test	P-value subgroup difference
Europe	19.3 [15.9-23]	[5.1-39.5]	3.6 [3.1-4.1]	24	6557	92.2 [89.7-94.1]	< 0.001	0.905	
Western Pacific	33.5 [30.2-36.8]	NA	NA	1	768	NA	1	NA	
**Sample type**									0.243
Nasal secretions	12.7 [3.9-25.4]	[0-100]	4.7 [3.1-6.9]	3	808	95.4 [89.9-97.9]	< 0.001	0.762	
Nasopharyngeal secretions	20 [17.3-22.8]	[7.2-37]	3.6 [3.2-4]	33	12159	92.2 [90.1-93.9]	< 0.001	0.441	
**HBoV**									
**Study period**									0.09
Continuous study period	9.4 [6.4-12.9]	[0.1-29.1]	4.5 [4-5.2]	19	6897	95.2 [93.6-96.3]	< 0.001	0.497	
Interrupted time series study	5.1 [2.2-9.1]	[0-30.4]	2.4 [1.5-3.9]	4	985	82.9 [56.4-93.3]	0.001	0.386	
**WHO Region**									< 0.001
America	4.7 [0-15.2]	[0-100]	3.8 [2.4-5.9]	3	525	93.1 [83.1-97.2]	< 0.001	0.11	
Eastern Mediterranean	4 [2.9-5.3]	NA	1	2	1072	0	0.913	NA	
Europe	9 [5.4-13.5]	[0-33.4]	4.9 [4.3-5.7]	16	4947	95.9 [94.5-96.9]	< 0.001	0.884	
Western Pacific	11.6 [10.3-13]	[4.3-21.7]	1 [1-2.9]	3	2162	0 [0-88.3]	0.411	0.47	
**Sample type**									0.545
Nasal secretions	6.6 [2.7-12]	[0-97]	2.6 [1.5-4.5]	3	808	85.6 [57.9-95.1]	0.001	0.344	
Nasopharyngeal secretions	8.4 [5.4-12]	[0-29.4]	5 [4.4-5.7]	20	7502	96 [94.9-96.9]	< 0.001	0.784	
Throat secretions	9.8 [7.1-13]	NA	NA	1	396	NA	1	NA	
**HAdV**									
**Study period**									0.081
Continuous study period	6.6 [4.7-8.8]	[0.3-18.8]	3 [2.5-3.5]	22	5786	88.6 [84.1-91.8]	< 0.001	0.081	
Interrupted time series study	3.7 [1.6-6.5]	[0-20.2]	1.9 [1.1-3.2]	4	948	72 [20.7-90.1]	0.013	0.501	
**WHO Region**									< 0.001
America	1.5 [0.4-3.2]	[0-31]	1.2 [1-3.6]	3	525	24.9 [0-92.2]	0.264	0.454	

	Prevalence [95% CI]	95% Prediction interval	H [95% CI]	N Studies	N Cases	I2 [95% CI]	P-Value Heterogeneity	P-Value Egger test	P-value subgroup difference
Eastern Mediterranean	6.9 [4.6-9.6]	[0-51.6]	1.6 [1-3]	3	1240	61.8 [0-89.1]	0.073	0.177	
Europe	6.3 [4.5-8.4]	[0.7-16.4]	2.3 [1.9-2.9]	18	3931	81.8 [72.2-88]	< 0.001	0.543	
Northern America	5.9 [3.4-9.1]	NA	NA	1	270	NA	1	NA	
Western Pacific	18.1 [15.5-20.9]	NA	NA	1	768	NA	1	NA	
**Sample type**									0.532
Nasal secretions	7.3 [4.6-10.4]	NA	NA	1	316	NA	1	NA	
Nasopharyngeal secretions	6 [4.2-8.1]	[0.1-18.5]	3.1 [2.6-3.6]	25	6418	89.3 [85.5-92.1]	< 0.001	0.092	
**HPIV**									
**Study period**									0.005
Continuous study period	6.7 [4.6-9.1]	[0.1-20.5]	3.3 [2.8-3.9]	20	5687	91 [87.5-93.5]	< 0.001	0.943	
Interrupted time series study	2.8 [1.4-4.6]	[0-10.3]	2.2 [1.6-3.1]	8	2246	79.3 [59.6-89.4]	< 0.001	0.622	
**WHO Region**									< 0.001
America	6 [0.8-15]	[0-67.2]	4.4 [3.1-6.2]	4	980	94.8 [89.8-97.4]	< 0.001	0.166	
Eastern Mediterranean	11.6 [6.8-17.4]	[0-95.4]	2.7 [1.6-4.6]	3	1240	86.4 [60.6-95.3]	0.001	0.424	
Europe	3.9 [2.7-5.3]	[0.3-10.8]	2.1 [1.7-2.7]	19	4675	78.3 [66.7-85.9]	< 0.001	0.283	
Northern America	8.1 [5.2-11.7]	NA	NA	1	270	NA	1	NA	
Western Pacific	13.7 [11.3-16.2]	NA	NA	1	768	NA	1	NA	
**Sample type**									0.086
Nasal secretions	2.8 [0.8-5.9]	[0-76.1]	2.1 [1.2-3.8]	3	808	78.1 [29.5-93.2]	0.01	0.718	
Nasopharyngeal secretions	5.8 [4-7.9]	[0-19]	3.4 [2.9-3.9]	25	7125	91.4 [88.5-93.5]	< 0.001	0.969	
**HMPV**									
**Study period**									0.005
Continuous study period	6.3 [5.2-7.4]	[2.6-11.2]	1.7 [1.3-2.1]	24	7018	64.3 [44.8-76.9]	< 0.001	0.012	
Interrupted time series study	3.1 [1.5-5.1]	[0-11.8]	2.3 [1.6-3.3]	7	2066	81.4 [62.6-90.8]	< 0.001	0.735	

	Prevalence [95% CI]	95% Prediction interval	H [95% CI]	N Studies	N Cases	I2 [95% CI]	P-Value Heterogeneity	P-Value Egger test	P-value subgroup difference
**WHO Region**									0.443
America	5.4 [1.6-11.1]	[0-33.5]	3 [2.1-4.4]	5	1036	89 [77-94.7]	< 0.001	0.117	
Eastern Mediterranean	6.2 [4-8.9]	NA	NA	1	369	NA	1	NA	
Europe	5.7 [4.4-7.2]	[1.2-12.8]	2 [1.7-2.5]	21	5355	76.1 [63.6-84.3]	< 0.001	0.017	
Northern America	3.3 [1.5-5.9]	NA	NA	1	270	NA	1	NA	
South-East Asia	3.5 [1.2-6.9]	NA	NA	1	170	NA	1	NA	
Western Pacific	5.5 [4.5-6.7]	[0-20.3]	1.3 [1-2.3]	3	2708	38.6 [0-80.8]	0.196	0.692	
**Sample type**									0.198
Nasal secretions	3.7 [1.4-7]	[0-25.9]	2.8 [1.8-4.4]	4	1750	87.6 [70.5-94.8]	< 0.001	0.655	
Nasopharyngeal secretions	5.7 [4.6-6.8]	[1.6-11.8]	2 [1.7-2.4]	28	8158	74.7 [63.5-82.5]	< 0.001	0.017	
**Influenza**									
**Study period**									0.014
Continuous study period	4 [2.6-5.8]	[0-12.8]	2.5 [2-3.1]	16	4325	84.4 [76.1-89.9]	< 0.001	0.055	
Interrupted time series study	1.9 [1-3]	[0-5.9]	1.6 [1.1-2.4]	8	2246	62.8 [20-82.7]	0.009	0.313	
**WHO Region**									0.058
America	2.8 [0.5-6.4]	[0-28]	2.5 [1.6-4]	4	980	83.9 [59.4-93.6]	< 0.001	0.426	
Eastern Mediterranean	1.1 [0.2-2.5]	NA	NA	1	369	NA	1	NA	
Europe	3.5 [2.2-5]	[0-11.7]	2.4 [2-3]	18	4454	83.3 [74.8-88.9]	< 0.001	0.034	
Western Pacific	3.3 [2.1-4.6]	NA	NA	1	768	NA	1	NA	
**Sample type**									0.677
Nasal secretions	2.2 [0-9.2]	[0-100]	4.5 [3-6.7]	3	808	95 [88.8-97.8]	< 0.001	0.74	
Nasopharyngeal secretions	3.2 [2.3-4.4]	[0.2-9.1]	2.1 [1.7-2.6]	21	5763	77.3 [65.7-85]	< 0.001	0.006	
**HCoV**									
**Study period**									0.589
Continuous study period	3.1 [2-4.4]	[0-9.8]	2.3 [1.9-2.8]	20	5365	80.9 [71.3-87.2]	< 0.001	0.789	

	Prevalence [95% CI]	95% Prediction interval	H [95% CI]	N Studies	N Cases	I2 [95% CI]	P-Value Heterogeneity	P-Value Egger test	P-value subgroup difference
Interrupted time series study	2.5 [0.8-5.1]	[0-14.6]	3 [2.2-4]	7	2066	88.7 [79.2-93.9]	< 0.001	0.717	
**WHO Region**									< 0.001
America	1.5 [0.8-2.4]	[0.2-3.7]	1 [1-2]	4	980	0 [0-75.5]	0.598	0.625	
Eastern Mediterranean	4.2 [1.4-8.4]	NA	2.7 [1.4-5.4]	2	1072	86.5 [46.7-96.6]	0.006	NA	
Europe	2.6 [1.5-4.1]	[0-10.4]	2.4 [2-3]	18	4223	83 [74.3-88.8]	< 0.001	0.639	
Northern America	10.7 [7.3-14.7]	NA	NA	1	270	NA	1	NA	
Western Pacific	4.4 [2.4-7]	NA	1.3 NA	2	886	37.7 NA	0.205	NA	
**Sample type**									0.071
Nasal secretions	1.1 [0-3.3]	[0-70]	2.1 [1.2-3.8]	3	808	77.7 [27.9-93.1]	0.011	0.79	
Nasopharyngeal secretions	3.2 [2.2-4.4]	[0-10.3]	2.4 [2-2.9]	24	6623	82.8 [75.5-88]	< 0.001	0.969	
**EV**									
**Study period**									0.466
Continuous study period	2.6 [1-4.7]	[0-13.3]	3.1 [2.4-3.9]	11	3254	89.4 [83-93.4]	< 0.001	0.716	
Interrupted time series study	3.6 [2.1-5.5]	[0-12.1]	1.3 [1-2.3]	4	948	44.1 [0-81.3]	0.147	0.304	
**WHO Region**									< 0.001
America	20.8 [12.4-30.6]	NA	NA	1	77	NA	1	NA	
Eastern Mediterranean	1.6 [0.5-3.2]	NA	NA	1	369	NA	1	NA	
Europe	2 [1.2-2.9]	[0.2-5.2]	1.5 [1.1-2.1]	12	2988	55.3 [14.5-76.6]	0.01	0.632	
Western Pacific	7.8 [6-9.8]	NA	NA	1	768	NA	1	NA	
**Sample type**									NA
Nasopharyngeal secretions	2.9 [1.6-4.5]	[0-11.4]	2.7 [2.2-3.3]	15	4202	86.1 [78.6-90.9]	< 0.001	0.886	

CI: confidence interval; RV: Rhinovirus; HCoV: Human Coronavirus; HPIV: Human Parainfluenzavirus; HMPV: Human Metapneumovirus; HRSV: Human Respiratory Syncytial Virus; HAdV: Human Adenovirus; HBoV: Human Bocavirus; EV: Enterovirus; NA: not applicable.
