# Supplementary figures and images for: Systematic review and meta-analysis of the prevalence of common respiratory viruses in children < 2 years with bronchiolitis in the pre-COVID-19 pandemic era
Source: PLoS One. 2020 Nov 12;15(11):e0242302. doi: 10.1371/journal.pone.0242302 (PMC7660462; doi:10.1371/journal.pone.0242302)

S1 Fig. Global prevalence of Respiratory Viruses in children < 2 years with bronchiolitis

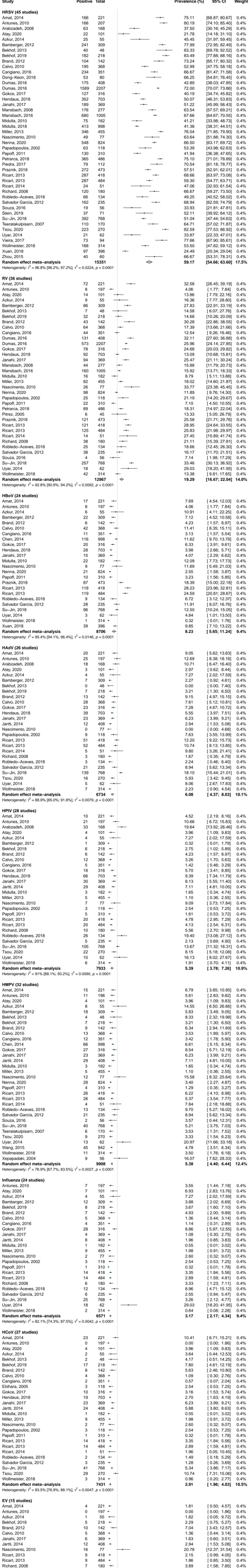

Supplement: S1 File — (ZIP) [file pone.0242302.s002.zip › S1 Fig.pdf]

S10 Fig. Funnel plot for publication for EV in people with bronchiolitis

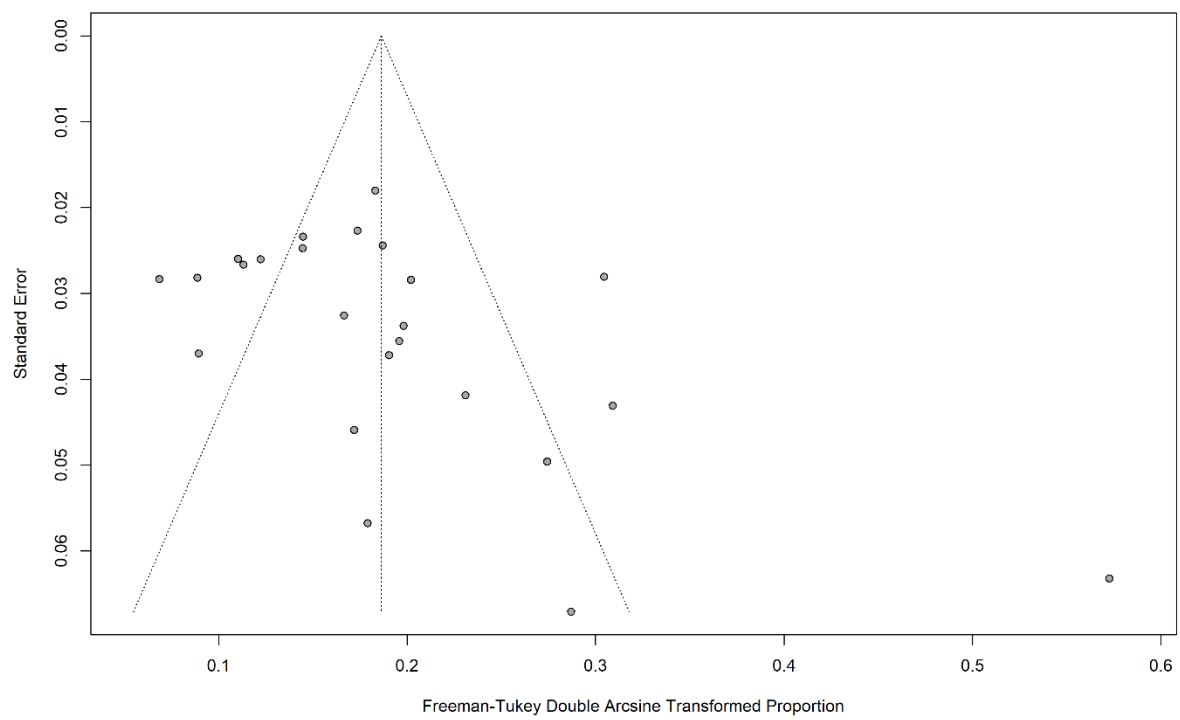

Supplement: S1 File — (ZIP) [file pone.0242302.s002.zip › S10 Fig.pdf]

S11 Fig. Funnel plot for publication for HCoV in people with bronchiolitis

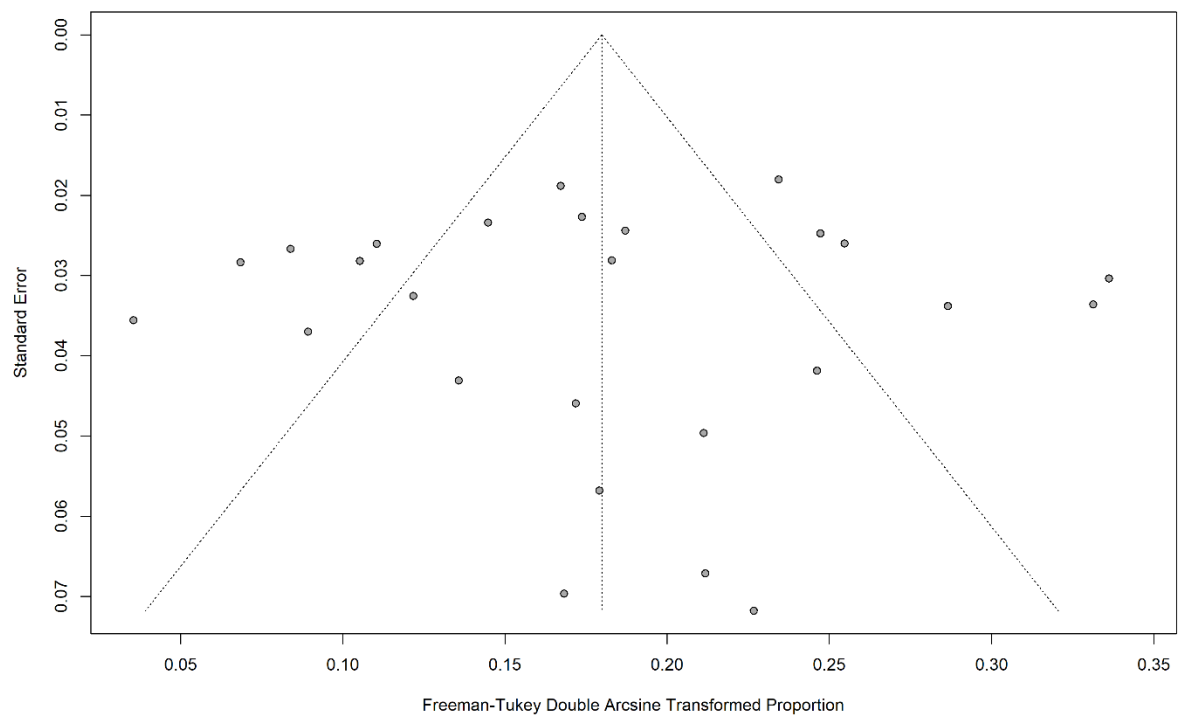

Supplement: S1 File — (ZIP) [file pone.0242302.s002.zip › S11 Fig.pdf]

S2 Fig. Codetection rate of viral infections among children < 2 years with bronchiolitis

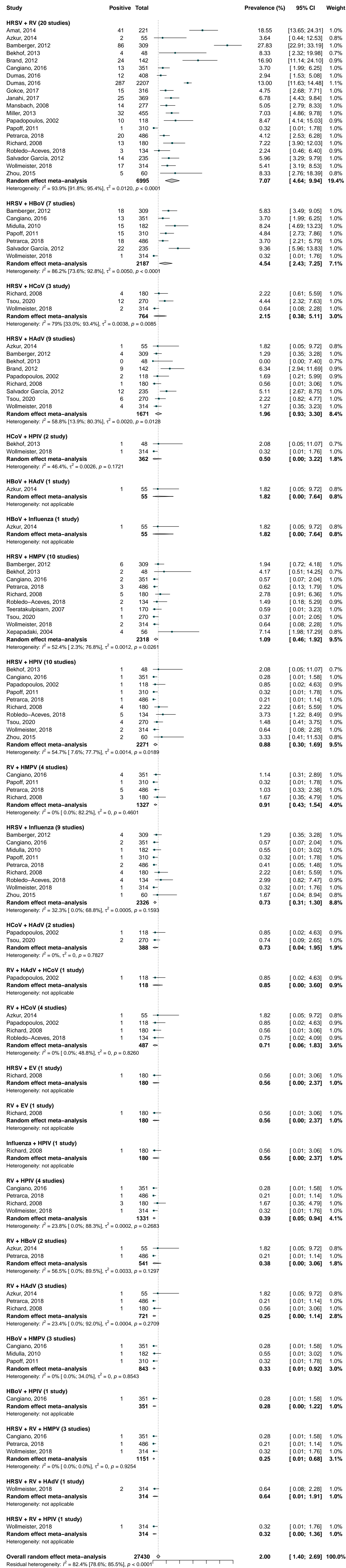

Supplement: S1 File — (ZIP) [file pone.0242302.s002.zip › S2 Fig.pdf]

S3 Fig. Funnel plot for publication for HRSV in people with bronchiolitis

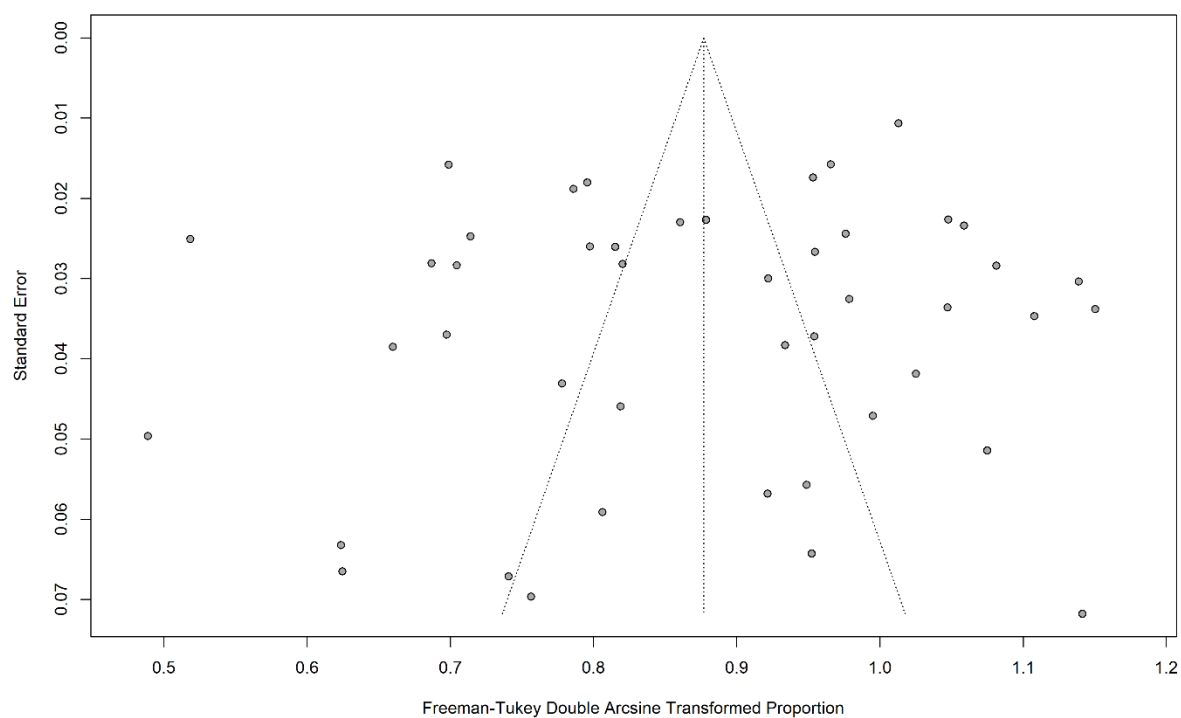

Supplement: S1 File — (ZIP) [file pone.0242302.s002.zip › S3 Fig.pdf]

S4 Fig. Funnel plot for publication for RV in people with bronchiolitis

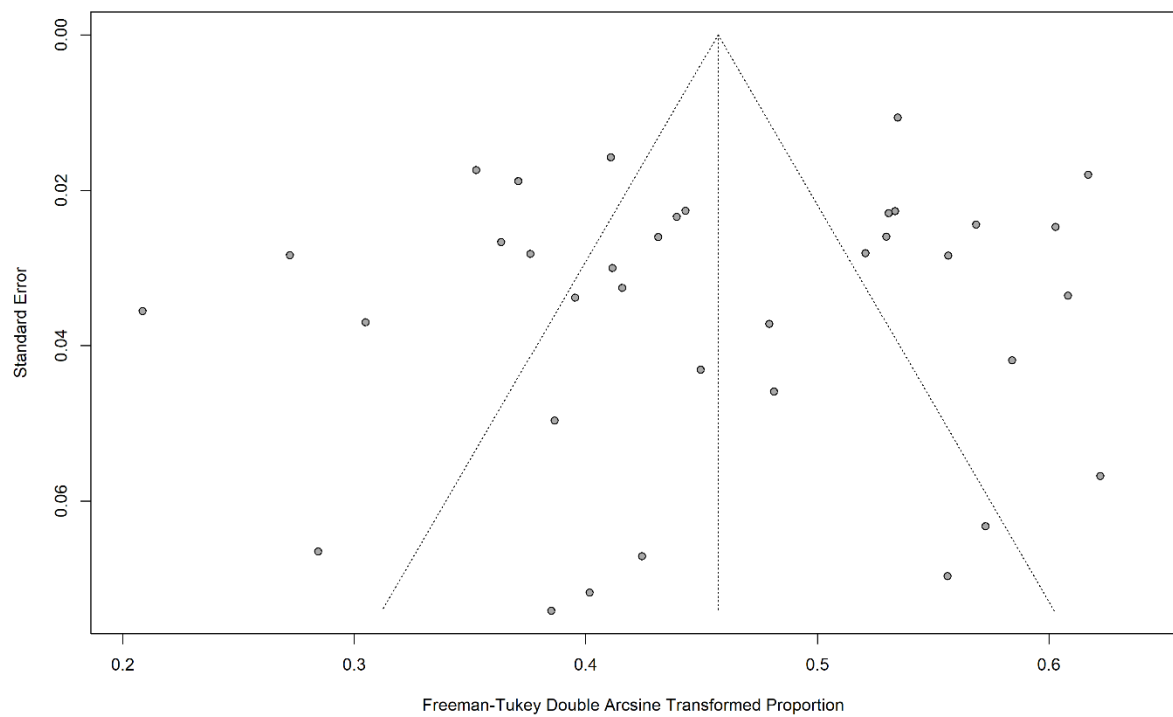

Supplement: S1 File — (ZIP) [file pone.0242302.s002.zip › S4 Fig.pdf]

S5 Fig. Funnel plot for publication for HBoV in people with bronchiolitis

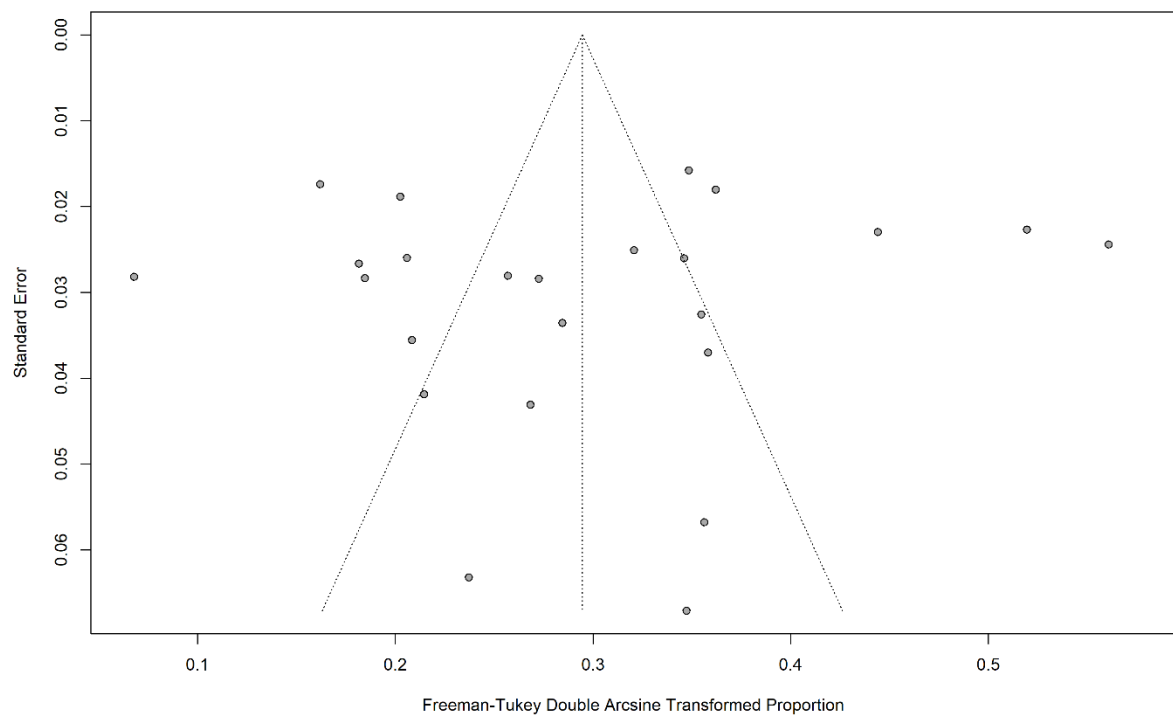

Supplement: S1 File — (ZIP) [file pone.0242302.s002.zip › S5 Fig.pdf]

S6 Fig. Funnel plot for publication for HAdV in people with bronchiolitis

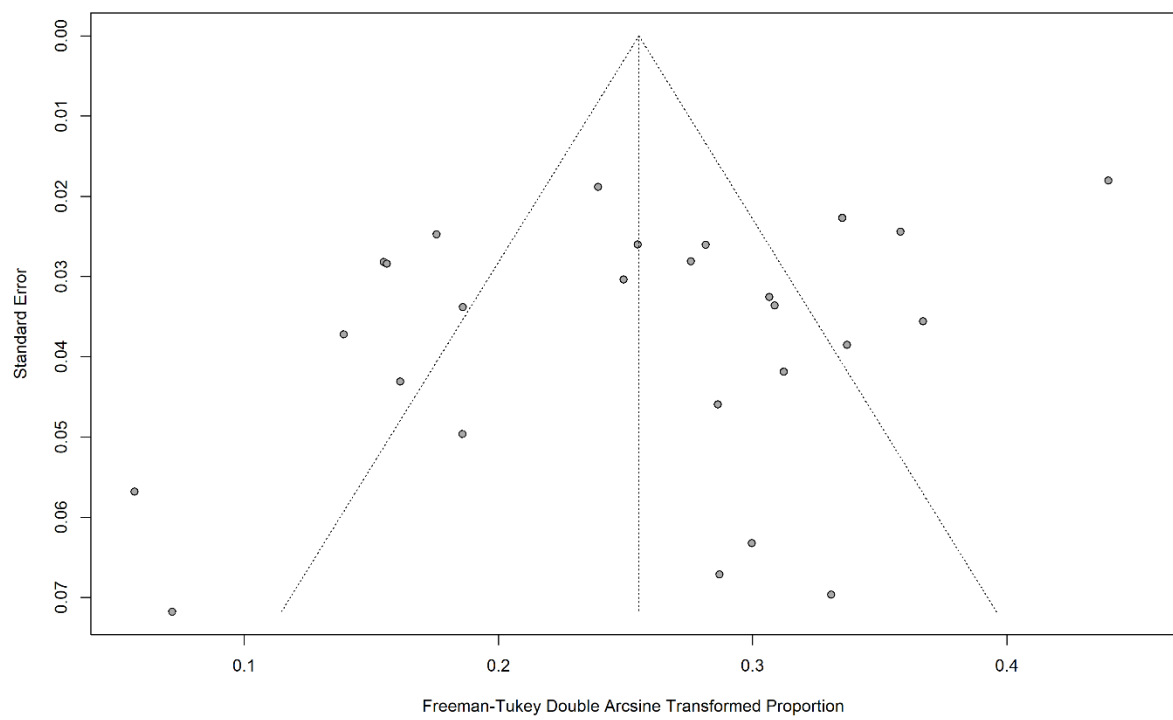

Supplement: S1 File — (ZIP) [file pone.0242302.s002.zip › S6 Fig.pdf]

S7 Fig. Funnel plot for publication for HMPV in people with bronchiolitis

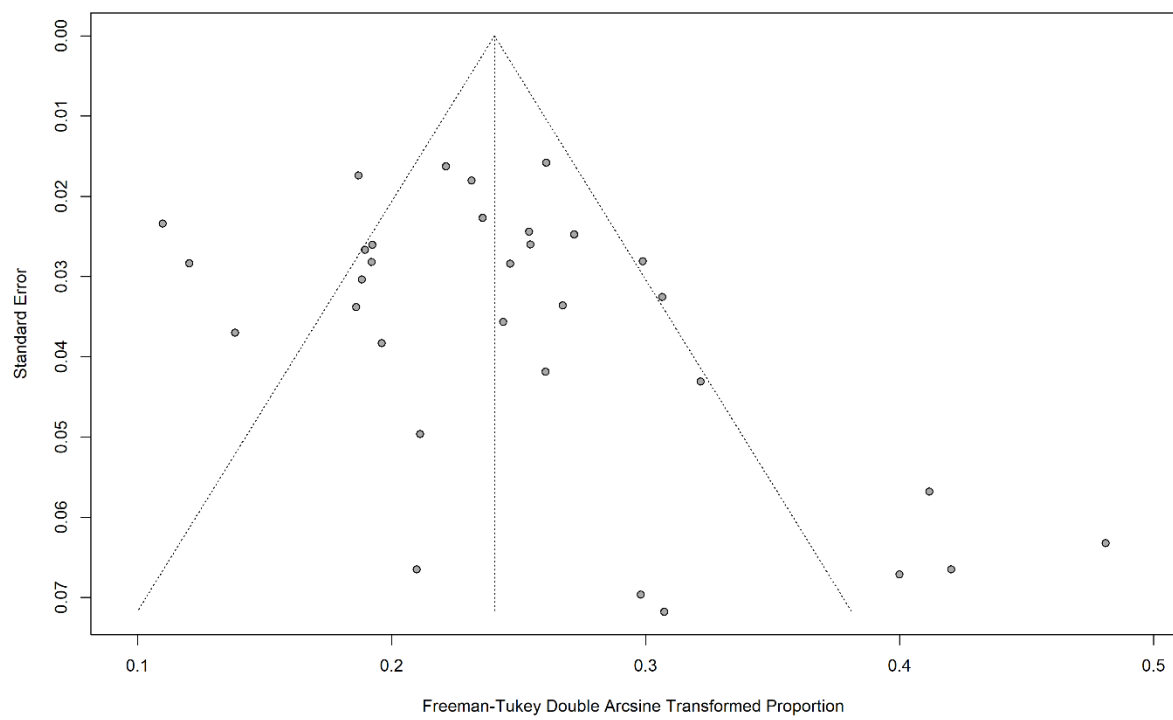

Supplement: S1 File — (ZIP) [file pone.0242302.s002.zip › S7 Fig.pdf]

S8 Fig. Funnel plot for publication for HPIV in people with bronchiolitis

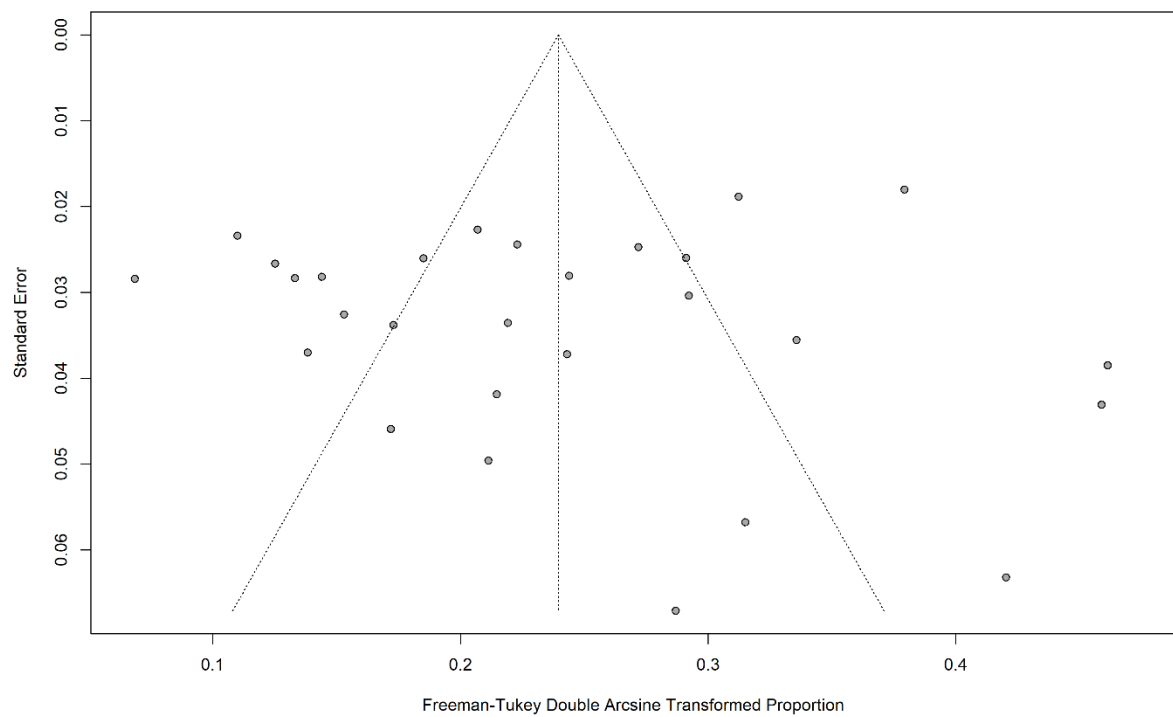

Supplement: S1 File — (ZIP) [file pone.0242302.s002.zip › S8 Fig.pdf]

S9 Fig. Funnel plot for publication for Influenza in people with bronchiolitis

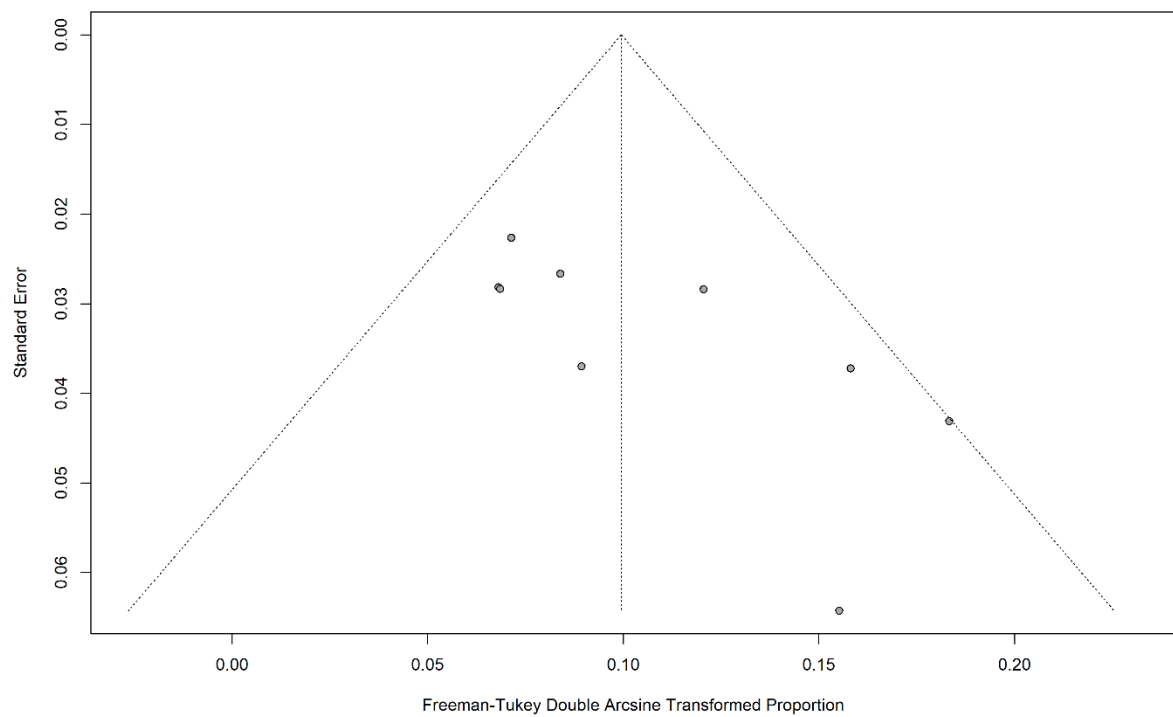

Supplement: S1 File — (ZIP) [file pone.0242302.s002.zip › S9 Fig.pdf]
