# Supplementary material for: Systematic review and meta-analysis of the prevalence of common respiratory viruses in children < 2 years with bronchiolitis in the pre-COVID-19 pandemic era
Source: PLoS One. 2020 Nov 12;15(11):e0242302. doi: 10.1371/journal.pone.0242302 (PMC7660462; doi:10.1371/journal.pone.0242302)
Supplement: Attachment — Submitted filename: Response to Reviewers PONE-D-20-26375.doc [file pone.0242302.s003.doc]

**Review Comments to the Author**

Editor: The revised manuscript has adequately addressed the majority of the comments of the reviewers. However, I do not agree with your response to the comment of reviewer 3, which was reiterated in my decision letter. The COVID-19 outbreak was declared a pandemic in March 2020, followed by several months of lockdowns and high pressure on global health research infrastructures. Therefore, the argument that the search for articles was continued until August 2020 does not justify interpretation of the results of this systematic review in the context of the COVID-19 pandemic. All available reports were based on pre-pandemic data.

PLOS ONE explicitly considers systematic review papers for publication, as described in the submission guidelines (https://journals.plos.org/plosone/s/submission-guidelines#loc-systematic-reviews-and-meta-analyses). In my opinion, this section is of crucial importance: “A systematic review paper, as defined by The Cochrane Collaboration, is a review of a clearly formulated question that uses explicit, systematic methods to identify, select, and critically appraise relevant research, and to collect and analyze data from the studies that are included in the review.” Based on this, I cannot accept your objective, as stated in the abstract: “This systematic review and meta-analysis was initiated to clarify the prevalence of respiratory viruses in children with bronchiolitis in the coronavirus disease 2019 pandemic context.” It will be impossible to perform a systematic review with that aim until well into next year.

I therefore ask you to revise the manuscript and remove all reference to the COVID-19 pandemic from the title, abstract, introduction and results section of your manuscript. I believe that it would be justified to add a paragraph at the end of the discussion in which you discuss the potential impact of the COVID-19 pandemic on bronchiolitis, and the necessity to collect data on this topic.

Authors: We thank the Editor for these suggestions, the text is now modified accordingly.

Moreover, please make sure that PRISMA flow diagram is included as Fig 1 and the PRISMA checklist as supporting information, as specified in the instructions for authors.

Authors: We thank the Editor for this suggestion, Fig 1 is now shown in the S1 table.

Finally, make sure that your figure TIF files are correctly uploaded to the system, as the current PDF file reports file errors and does not display your figures.

Authors: New figures in TIFF formats have been uploaded to the system, thank you.
